# Cost-effectiveness of community-based repurposed therapies for COVID-19 in the PRINCIPLE trial

**DOI:** 10.1186/s12962-026-00809-x

**Published:** 2026-08-01

**Authors:** May Ee Png, Victoria Harris, F. D. Richard Hobbs, Paul Little, Ly-Mee Yu, Christopher C. Butler, Stavros Petrou

**Affiliations:** 1 Nuffield Department of Primary Care Health Sciences, University of Oxford, Oxford, UK; 2 Nuffield Department of Orthopaedics, Rheumatology and Musculoskeletal Sciences, University of Oxford, Oxford, UK; 3 Primary Care Research Centre, University of Southampton, Southampton, UK

**Keywords:** COVID-19, Cost-effective, Repurposed drug

## Abstract

**Background:**

During the early phases of the COVID-19 pandemic, health systems faced urgent decisions about allocating resources to repurposed community treatments in the absence of definitive evidence. While large platform trials reported clinical outcomes, their implications for cost-effective resource allocation under uncertainty remain unclear. Using the Platform Randomised trIal of treatmeNts in the Community for epIdemic and Pandemic iLlnEsses (PRINCIPLE) trial as a case study, we evaluated the cost-effectiveness of six repurposed drugs compared with usual care.

**Methods:**

Trial-based economic analyses assessed National Health Service (NHS) costs and clinical outcomes over 28 days post-randomisation using multiply imputed data. Incremental cost-effectiveness ratios (ICERs) were estimated as incremental cost per day saved until recovery and per hospitalisation or death avoided. Sensitivity analyses explored decision uncertainty.

**Results:**

A total of 8825 participants randomised between April 3, 2020 and July 1, 2022 were included. Inhaled budesonide showed a favourable balance across outcomes, with ICERs of £6,386 per hospitalisation or death avoided and -£77 per day saved until recovery, indicating lower costs and faster recovery than usual care. Colchicine had a low ICER for hospitalisation or death avoided (£2,858), but limited recovery benefit. Azithromycin and doxycycline were cost-saving for recovery but had high ICERs for hospitalisation or death avoided (£27,490 and £21,278, respectively). Favipiravir was associated with lower costs and faster recovery but higher cost for a modest reduction in hospitalisation or death avoided than usual care. Ivermectin was associated with higher costs for both outcomes and had a low probability of cost-effectiveness. Sensitivity analyses largely supported these findings.

**Conclusions:**

Trial-based economic evidence from large platform trials can inform efficient resource allocation during public health emergencies, including identifying interventions that offer limited or no economic value. These findings highlight the importance of integrating economic evaluation into rapid trial platforms to support timely funding and disinvestment decisions under uncertainty.

**Trial registration:**

ISRCTN registry, ISRCTN86534580, registered 23 March 2020.

**Supplementary Information:**

The online version contains supplementary material available at https://doi.org/10.1186/s12962-026-00809-x.

## Introduction

Public health emergencies require rapid allocation of limited health care resources in the presence of substantial uncertainty. During the early phases of the COVID-19 pandemic, multiple existing medicines were repurposed for community use in higher-risk patients, often before robust evidence on clinical effectiveness or value for money was available. Understanding the economic consequences of such decisions is essential for improving future pandemic preparedness and resource allocation.

The Platform Randomised trIal of treatmeNts in the Community for epIdemic and Pandemic iLlnEsses (PRINCIPLE) trial (ISRCTN86534580) was an open-label, multi-arm, prospective, platform, adaptive, randomised controlled clinical trial designed to evaluate repurposed treatments for acute COVID-19 among higher-risk individuals (i.e., individuals aged 18 years and above with comorbidities or aged 65 years and above without comorbidities) in the community [1]. This trial received approval from the UK Medicines and Healthcare products Regulatory Agency and the South Central-Berkshire Research Ethics Committee (Ref:20/SC/0158). The trial was conducted largely before the COVID vaccination programme started on December 8, 2020, and during periods of different variants predominance [2, 3]. It provided rapid evidence on clinical outcomes across multiple interventions tested sequentially within a single infrastructure. While most evaluated treatments, including azithromycin, doxycycline, colchicine, favipiravir, and ivermectin, did not demonstrate meaningful clinical benefit, inhaled budesonide was associated with faster recovery [4–9]. 

Beyond clinical effectiveness, such platform trials offer a unique opportunity to examine the economic implications of adopting, continuing, or discontinuing interventions during a public health emergency. Economic evaluation in this context can identify interventions that represent poor value for money, quantify opportunity costs, and support timely disinvestment decisions, all of which are critical for efficient resource allocation under constrained conditions.

This study uses data from the PRINCIPLE trial to evaluate the cost-effectiveness of six repurposed community treatments compared with usual care from the perspective of the UK National Health Service (NHS). Rather than informing current COVID-19 treatment decisions, it provides a retrospective economic evaluation within a large adaptive platform trial and illustrates the value of embedding economic evaluation alongside clinical effectiveness assessments in trials conducted during public health emergencies. Reporting follows the 2022 Consolidated Health Economic Evaluation Reporting Standards (CHEERS) checklist [10]. 

## Methods

### Treatment arms

PRINCIPLE was an adaptive platform randomised trial with response adaptive randomisation, with additional interventions added over time. The treatment arms included were usual care or usual care plus either hydroxychloroquine; (azithromycin arm); (doxycycline arm); inhaled budesonide; (colchicine arm); (favipiravir arm); or ivermectin. The candidate therapies were selected on the basis of evidence indicating they might be ‘repurposed’ to treat COVID-19 patients. The hydroxychloroquine arm was excluded as it was stopped prematurely by the UK regulator.

Usual care, the comparator arm, was defined as the currently recommended treatment delivered by responsible clinicians and followed current National Health Service (NHS) care provision. The other treatment arms were the intervention arms, as described in the Supplementary Material - Additional Methodological Details.

### Trial population

Participants were recruited through general practices, health service helplines, COVID-19 ‘hot hubs’ and directly through the trial website. The inclusion and exclusion criteria for each intervention have been described previously [4–9]. Briefly, all participants were not hospitalised and had ongoing symptoms that had started within the previous 14 days from suspected COVID-19 (in accordance with the UK NHS definition of high temperature and/or new, continuous cough and/or change in sense of smell/ taste) or that had polymerase chain reaction (PCR)-confirmed COVID-19. Exclusion criteria included prior randomisation within the PRINCIPLE trial, current use of the intervention, previous adverse reaction to the intervention, pregnancy, or breastfeeding.

No separate sample size calculation was performed for the economic evaluation; analyses were conducted on the available PRINCIPLE trial population, with sample sizes determined by the adaptive clinical trial design and prespecified stopping rules rather than economic endpoints.

### Measurement of resource use

The frequency of use of primary care and secondary care resources were documented in the 28-day diary and trial questionnaires at 3, 6, and 12 months, complemented with general practitioner (GP) notes review through the Oxford Royal College of General Practitioners Research Surveillance Centre (RCGP RSC) Oxford RCGP Clinical Informatics Digital Hub (ORCHID) or Admitted Patient Care and Critical Care data from NHS Digital. The data sources were considered in hierarchy: (1) participant reported (daily diaries, calls, hospitalisation case report form), (2) ORCHID notes review, and (3) data from NHS Digital, as per the data hierarchy stated in the Master Statistical Analysis Plan (MSAP) [11]. Time off work due to COVID-19 symptoms was also captured in the trial questionnaires.

### Valuation of resource use

Resource inputs were valued in monetary terms using the latest UK unit costs or participant valuations at the time of analysis (Supplementary Table S1), adjusted for inflation using the Personal Social Services Research Unit (PSSRU) Hospital and Community Health Services Index [12] to 2022/23£ prices. Unit costs of trial treatments and medications were obtained from the Prescription Cost Analysis (PCA) [13] database or literature if they were not available in the database. The costs of dispensing the trial medications were excluded because these were considered to be approximately equal between the comparator and interventions arms. NHS reference costs [14] were employed to value hospital resource inputs while the PSSRU Unit Costs of Health and Social Care [12] compendium was used to value primary care services. Daily gender-specific median wages, obtained from the Office for National Statistics, were used in the computation of economic values for time taken off work by participants due to COVID-19 [15].

### Measurement of outcomes

The trial had two co-primary clinical outcomes recorded: time taken to the first instance of self-reported feeling recovered (from daily diaries for 28 days), and hospitalisations (overnight non-elective stays) or all-cause death avoided (from daily diaries and trial questionnaires at six and 12 months).

### Data cleaning

Records of resource use across different time-points were cross-checked to ensure that there was no duplication. Corrections made were documented in the statistical code.

### Missing data

Following Faria et al. [16] data was treated as missing at random (MAR) and the multiple imputation method for missing baseline covariates, costs and clinical outcomes used chained regression equations [17] using Amelia II in R [18]. This included key independent variables (treatment allocation and baseline covariates including sex, ethnicity, vaccination status, smoking status, presence of comorbidities, age category, duration of symptoms, severity of symptoms at baseline, and use of an inhaled corticosteroid at randomisation or during 28 days of follow-up). The imputation was run 10 times. Pooled estimates were then computed using Rubin’s rules [16] to obtain overall mean estimates of the costs and clinical outcomes per participant.

### Data analysis

The base case (‘primary analysis population’) included all eligible SARS-CoV-2-positive participants randomised to usual care or an intervention, analysed according to the intention-to-treat principle. Each intervention was evaluated in a pairwise comparison with usual care, consistent with the study objective of estimating the cost-effectiveness of each intervention relative to usual care rather than identifying the most cost-effective intervention across all treatment arms. Analyses used imputed cost and outcome data over 28 days (base case) and 12 months (sensitivity). The economic evaluation followed a prespecified Health Economic Analysis Plan (HEAP; supplementary material).

Resource use was summarised by trial arm and follow-up period. Differences were tested using t-tests for continuous variables and Pearson chi-squared (χ^2^) test for categorical variables. Mean differences in costs and outcomes were estimated using t-tests and 95% confidence intervals from 1000 bootstrap replications. No discounting was applied given the 12-month horizon, and statistical significance was set at *p* < 0.05.

Regression models were fitted to imputed data: linear regression for total costs, Cox proportional hazards for time-to-recovery, and seemingly unrelated regression to jointly estimate NHS costs and hospitalisation/death, allowing for correlation between outcomes. Models were adjusted for vaccination status, subgroup covariates (age, comorbidity, symptom duration), and baseline costs (antibiotics, inhaled drugs, primary care, NHS111, emergency and outpatient services) as appropriate.

Incremental cost-effectiveness ratios (ICERs) were calculated as incremental cost per day of recovery gained and incremental cost per hospitalisation or death avoided. Time to recovery was analysed as the number of days to first reported recovery. Mean time to recovery was estimated for each group, and differences were calculated as usual care minus intervention, such that positive differences indicate faster recovery (fewer days), and negative differences indicate slower recovery. Hospitalisation or death was modelled as a binary outcome. Differences therefore represent differences in the proportion of participants experiencing the event and were calculated as intervention minus usual care, such that negative differences indicate fewer events (i.e. better outcomes). All analyses were carried out using R version 4.4 [19]. 

### Uncertainty analyses

A nonparametric bootstrapping approach was used to determine the level of sampling uncertainty surrounding the mean incremental cost-effectiveness ratios (ICERs) by generating 10,000 estimates of incremental costs and outcomes. Decision uncertainty was characterised by estimating the probability that each treatment option was cost-effective at different cost-effectiveness thresholds and displayed graphically using cost-effectiveness acceptability curves (CEACs). There was no published literature on stated or revealed preferences for the primary clinical outcomes (i.e. avoiding COVID-19 hospitalisations or deaths and time-to-recovery from COVID-19) so we could not apply external cost-effectiveness thresholds that inform decision uncertainty.

### Sensitivity analyses

Four sensitivity analyses were undertaken to explore uncertainties surrounding key parameters of the economic evaluation. First, the study perspective was extended to a societal perspective that included indirect costs (e.g. valuation of time off work). Second, complete case analysis was used to assess the impact of missing data on the ICERs. Third, ICERs expressed in terms of incremental cost per hospitalisation or death avoided were computed over a 12-month follow-up period. Fourth, a concurrent randomisation analysis consisting of all eligible SARS-CoV-2-positive participants was conducted based on participants who were randomised to the usual care arm during the same time frame when the intervention was actively randomised and who were eligible for randomisation to the intervention.

### Subgroup analyses

Subgroup analyses were pre-specified in the master statistical and health economic analysis plans to explore heterogeneity in incremental cost-effectiveness by age, baseline comorbidity, symptom duration, symptom severity, and use of inhaled corticosteroids at randomisation or during 28 days of follow-up. Age categories reflected intervention-specific eligibility criteria: 50 to 64 years and 65 years and above for azithromycin, doxycycline, and budesonide, and below 65 years and 65 years and above for ivermectin, favipiravir, and colchicine. Inhaled corticosteroid use was based on participant report, supplemented by notes review or NHS Digital data where required.

### Patient and public involvement

We intend to disseminate the main results to trial participants and the public and will seek the PRINCIPLE trial’s patient and public involvement members in the development of an appropriate method of dissemination.

## Results

### Population

A total of 8825 participants were included in the economic evaluation, with 186 allocated to azithromycin, 435 to doxycycline, 787 to budesonide, 156 to colchicine, 1829 to favipiravir, 2157 to ivermectin, and 3275 to usual care. Baseline characteristics of the population is detailed in Supplementary Table S2. The population was largely unvaccinated until colchicine, favipiravir and ivermectin were introduced. The numbers in the concurrent randomisation analysis differed from the clinical papers [4–9] as only SARS-CoV-2-positive participants were included.

### Completion of cost and outcomes

There was an almost 100% completion rate for costs and outcomes at 28-days (Supplementary Table S3), but low resource completion rates at 3 and 6 months. Those who did not complete the questionnaire at one time-point commonly completed the questionnaire at another time-point.

### Resource utilisation and work absence

Across treatment comparisons, mean health and social care resource use and duration of time off work were broadly similar to usual care. Differences were mainly observed in medication use and hospital-based care during the first 28 days, with some sporadic differences at later follow-up periods (29 days-3 months, 3–6 months and 6–12 months). Full numerical details by resource use and follow-up period are provided in Supplementary Tables S4-S9.

### Costs

There were no meaningful differences in total mean costs from the NHS or societal perspectives for azithromycin, doxycycline or budesonide, across all follow-up periods, with estimates showing substantial uncertainty (Supplementary Tables S10-S12).

At 28 days, colchicine, favipiravir and ivermectin were associated with lower mean NHS costs compared with usual care (colchicine: £561 [SE 104] vs. £851 [SE 56], *p* = 0.015; favipiravir: £379 [SE 19] vs. £541 [SE 22], *p* < 0.001; ivermectin: £355 [SE 14] vs. £541 [SE 22], *p* < 0.001) (Supplementary Tables S13-S15).

At later timepoints, cost differences were less consistent. Favipiravir showed lower mean NHS (£49 [SE 10] vs. £78 [SE 11], *p* = 0.045) and societal (£341 [SE 39] vs. £449 [SE 36], *p* = 0.041) costs at 3–6 months. Ivermectin showed lower mean NHS costs at 3 months (£41 [SE 7] vs. £68 [SE 10], *p* = 0.032) and at 12 months (£68 [SE 10] vs. £110 [SE 15], *p* = 0.019), as well as lower societal costs at 12 months (£586 [SE 63] vs. £778 [SE 61], *p* = 0.028) (Supplementary Tables S13–S15).

### Outcomes

For time-to-recovery, assessed over the first 28 days, compared with usual care, azithromycin and budesonide were associated with shorter recovery times (7.57 [SE 0.51] vs. 9.62 [SE 0.53] days, *p* = 0.006, 95% CI -3.5 to -0.6; and 9.14 [SE 0.28] vs. 10.01 [SE 0.28] days, *p* = 0.029, 95% CI -1.7 to -0.1, respectively) (Supplementary Tables S16 and S18). Doxycycline showed no meaningful difference (Supplementary Table S17). Colchicine was associated with longer time to recovery (12.09 [SE 0.77] vs. 9.90 [SE 0.27] days, *p* = 0.009, 95% CI 0.6 to 3.8) (Supplementary Table S19). Favipiravir and ivermectin showed no meaningful differences in recovery time (Supplementary Tables S20 and S21).

For hospitalisation or death, assessed at 28 days and 12 months, compared with usual care, azithromycin, doxycycline and budesonide showed no differences at both time points (Supplementary Tables S16-S18). In contrast, colchicine was associated with fewer events at 28 days (mean difference − 0.065, *p* < 0.001, 95% CI − 0.098 to − 0.028) and 12 months (mean difference − 0.061, *p* = 0.003, 95% CI -0.098 to -0.019) (Supplementary Table S19). Favipiravir showed similar reductions at 28 days (mean difference − 0.029, *p* < 0.001, 95% CI -0.038 to -0.020) and 12 months (mean difference − 0.032, *p* < 0.001, 95% CI -0.042 to -0.021) (Supplementary Table S20). Similarly, ivermectin reduced hospitalisations or deaths at both timepoints (Supplementary Table S21).

### Cost-effectiveness results

**Fig. 1:**
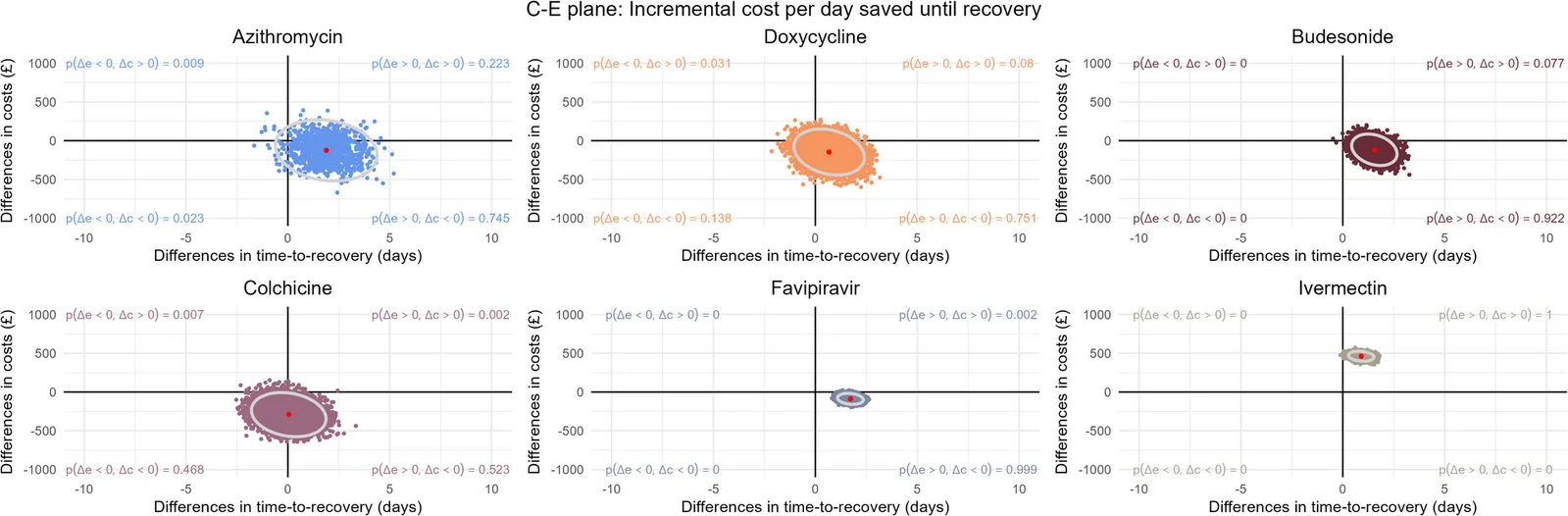
Base case cost-effectiveness analysis of interventions versus usual care in terms of incremental cost per day saved until recover

**Fig. 2:**
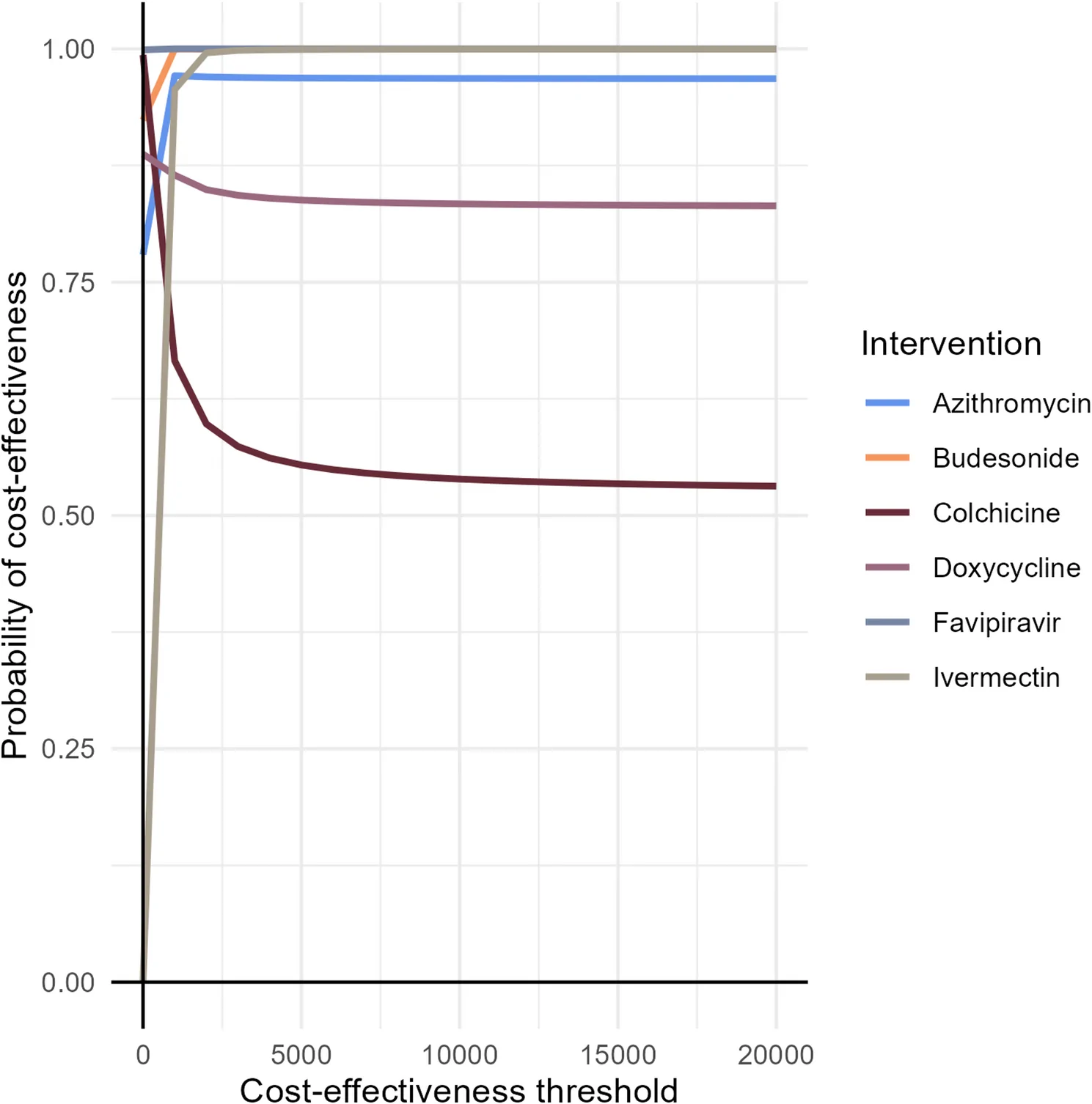
Cost-effectiveness acceptability curves for interventions versus usual care in terms of incremental cost per day saved until recovery. Cost-effectiveness threshold in £/day saved until recovery

The base case cost-effectiveness analysis results expressed as incremental cost per day saved until recovery (Table 1), showed that azithromycin, budesonide, doxycycline, and favipiravir were associated with lower costs and faster recovery than usual care (ICERs -£66, -£77, -£218, and -£51 per day saved, respectively), although uncertainty around both costs and outcomes means that cost-effectiveness depends on the cost-effectiveness threshold for an additional day saved until recovery. Colchicine was associated with lower costs and marginally faster recovery than usual care. Ivermectin was more costly and faster recovery than usual care, with an ICER of £509 per day saved. On the cost-effectiveness plane (Fig. 1), all treatment arms except ivermectin clustered predominantly in the south-east quadrant, while ivermectin clustered in the north-east quadrant. The CEACs showed high probabilities of cost-effectiveness for ivermectin, favipiravir, azithromycin, and budesonide across cost-effectiveness thresholds ≤£20,000 per day saved, while doxycycline and colchicine showed lower and more uncertain probabilities of cost-effectiveness (Fig. 2).

**Table 1:** Base case cost-effectiveness analysis results in terms of incremental cost per day saved until recovery

	Number		Cost							Time-to-recovery								ICER
	Intervention	Usual care	Intervention		Usual care		Mean difference	Lower 95%CI	Upper 95%CI		Intervention		Usual care		Mean difference	Lower 95%CI	Upper 95%CI	
			Mean	SE	Mean	SE					Mean	SE	Mean	SE				
Azithromycin	186	236	656.48	193.84	781.04	106.75	-124.56	-441.51	192.40		13.14	1.22	15.02	0.67	1.88	-0.11	3.88	-66
Doxycycline	435	336	747.13	72.29	894.02	97.77	-146.90	-384.49	90.70		14.78	0.48	15.45	0.53	0.67	-0.71	2.06	-218
Budesonide	787	1069	766.31	58.44	884.93	58.45	-118.61	-281.21	43.99		14.49	0.35	16.04	0.30	1.55	0.66	2.44	-77
Colchicine	156	1145	561.83	102.30	850.66	55.72	-288.84	-517.00	-60.67		16.06	0.70	16.11	0.30	0.04	-1.42	1.51	-6473
Favipiravir	1829	3256	452.76	18.94	541.43	21.80	-88.66	-145.29	-32.04		14.94	0.21	16.66	0.17	1.72	1.22	2.23	-51
Ivermectin	2157	3256	1003.06	14.82	541.43	21.97	461.73	409.59	513.68		15.74	0.20	16.65	0.17	0.91	0.41	1.41	509

All interventions except azithromycin and doxycycline were adjusted for age category, comorbidity, duration of illness, and vaccination status. Azithromycin and doxycycline were adjusted for age category, comorbidity, and duration of illness. For time-to-recovery, the mean difference was calculated as (usual care - intervention), such that positive values indicate fewer days to recovery with the intervention. Costs were additionally adjusted for baseline costs. Cost estimates are derived from separate seemingly unrelated regression (SUR) models for each outcome; therefore, cost values may differ slightly between tables. The adjusted mean costs and recovery times reported in the economic evaluation were estimated from the economic models and may therefore differ from the estimates reported in the unadjusted analyses. CI: confidence interval, ICER: incremental cost-effectiveness ratio, SE: standard error

**Fig. 3:**
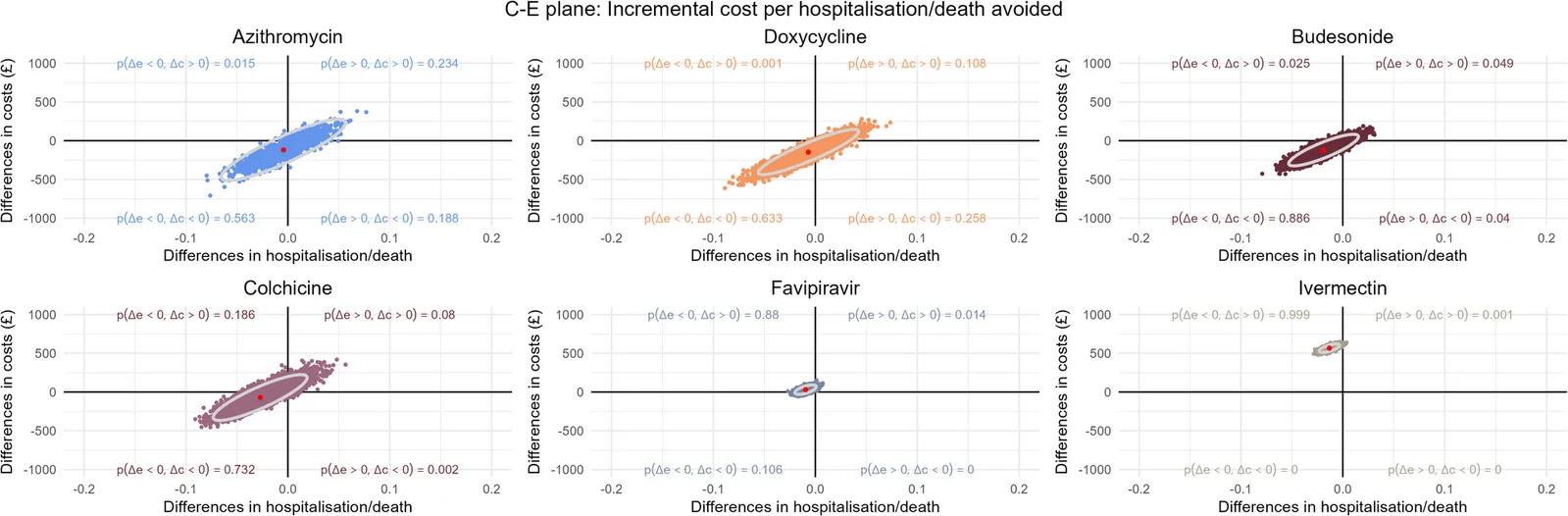
Base case cost-effectiveness analysis of interventions versus usual care in terms of incremental cost per hospitalisation or death avoided

**Fig. 4:**
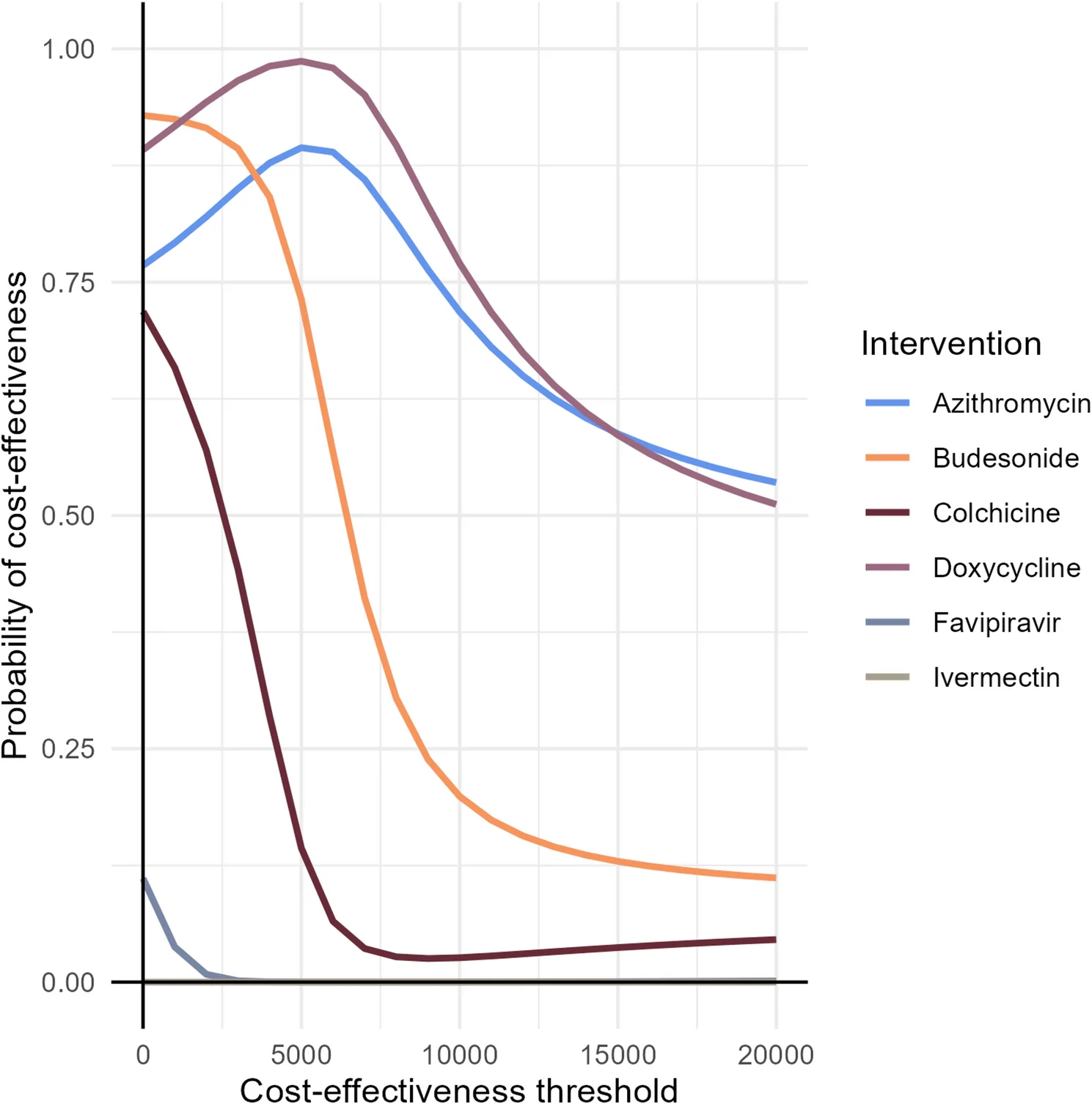
Cost-effectiveness acceptability curves for interventions versus usual care in terms of incremental cost per hospitalisation or death avoided. Cost-effectiveness threshold in £/ hospitalisation or death avoided

When outcomes were expressed as incremental cost per hospitalisation or death avoided (Table 2), favipiravir and ivermectin were associated with higher cost and fewer hospitalisations or death than usual care, with data points clustering predominantly in the north-west quadrant (Fig. 3) and probabilities of cost-effectiveness near 0% (Fig. 4). Colchicine (£2,585), budesonide (£6,396), doxycycline (£21,278), and azithromycin (£27,490) had favourable ICERs in comparison, with most estimates in the south-west quadrant (Fig. 3). Probabilities of cost-effectiveness exceeded 50% for azithromycin, doxycycline, budesonide, and colchicine at thresholds ≤£2,500 per hospitalisation or death avoided, but only doxycycline and azithromycin exceeded 50% at higher thresholds (£20,000) (Fig. 4).

**Table 2:** Base case cost-effectiveness analysis results in terms of incremental cost per hospitalisation or death avoided

	Number		Cost							Hospitalisation or death							ICER
	Intervention	Usual care	Intervention		Usual care		Mean difference	Lower 95%CI	Upper 95%CI	Intervention		Usual care		Mean difference	Lower 95%CI	Upper 95%CI	
			Mean	SE	Mean	SE				Mean	SE	Mean	SE				
Azithromycin	186	236	662.64	193.57	781.04	106.13	-118.40	-435.54	198.73	1.064	0.030	1.068	0.016	-0.004	-0.053	0.045	27,490
Doxycycline	435	336	746.61	12.26	894.02	12.26	-147.42	-381.24	86.41	1.082	0.002	1.089	0.002	-0.007	-0.046	0.032	21,278
Budesonide	787	1069	763.66	20.95	884.93	11.98	-121.27	-285.13	42.59	1.088	0.003	1.107	0.002	-0.019	-0.046	0.009	6,386
Colchicine	156	1145	780.72	19.61	850.66	11.39	-69.94	-306.86	166.98	1.076	0.003	1.103	0.002	-0.027	-0.064	0.010	2,585
Favipiravir	1829	3256	571.70	6.32	541.43	5.55	30.27	-18.35	78.89	1.035	0.001	1.045	0.001	-0.010	-0.018	-0.0012	-3,157
Ivermectin	2157	3256	1107.34	7.55	541.41	5.27	565.93	521.82	610.05	1.032	0.001	1.045	0.001	-0.013	-0.021	-0.005	-43,104

All interventions except azithromycin and doxycycline were adjusted for age category, comorbidity, duration of illness, and vaccination status. Azithromycin and doxycycline were adjusted for age category, comorbidity, and duration of illness. Costs were additionally adjusted for baseline costs. Cost estimates are derived from separate seemingly unrelated regression (SUR) models for each outcome; therefore, cost values may differ slightly between tables. The adjusted mean costs and recovery times reported in the economic evaluation were estimated from the economic models and may therefore differ from the estimates reported in the unadjusted analyses. CI: confidence interval, ICER: incremental cost-effectiveness ratio, SE: standard error

### Sensitivity and subgroup analyses

The findings from the sensitivity and subgroup analyses were directionally consistent with the base case analyses (Supplementary Tables S22-S33). For hospitalisation or death avoided over 28 days, cost-effectiveness varied by age, baseline risk, symptom duration, and inhaled corticosteroid use. In the concurrent randomisation analysis, azithromycin was less costly and more effective than usual care (dominant; ICER -£44,433) but was more costly and more effective among participants aged 50 to 64 years (ICER £853). Doxycycline was more costly and more effective among participants aged 50 to 64 years (ICER £2,411) and inhaled corticosteroid users (ICER £3,993) but was less costly and more effective among participants without comorbidity (ICER -£33,977), with fewer than 7 days of symptoms (ICER -£1,169), and without major symptoms (ICER -£35,020). Budesonide was more costly and more effective among participants aged 50 to 64 years (ICER £1,555) and those with shorter symptom duration (ICER £2,698) but was more costly and less effective among participants without major symptoms. In the concurrent randomisation analysis, colchicine was more costly and more effective than usual care (ICER £39,892), with higher ICERs among inhaled corticosteroid users (ICER £183,501). Favipiravir was more costly and more effective in the concurrent randomisation analysis (ICER £28,798) but was less costly and less effective in sensitivity analyses using a 12-month time horizon (ICER £12,762) or a societal perspective (ICER £59,984), and among participants aged 65 years and above (ICER £3,513) and inhaled corticosteroid users (ICER £245). Ivermectin was less costly but less effective under a societal perspective (ICER £11,929).

For time to recovery within 28 days, azithromycin was generally associated with lower costs and faster recovery across most subgroups, although among participants without major symptoms, it was associated with lower costs but slower recovery (ICER £482 per day saved). Doxycycline was more costly and more effective among participants aged 50 to 64 years (ICER £28 per day saved), but less costly and less effective among participants with at least one major symptom (ICER £881 per day saved). Budesonide was more costly and more effective among participants aged 50 to 64 years (ICER £15 per day saved), those with symptoms for fewer than 7 days before randomisation (ICER £105 per day saved), and those without major symptoms at baseline (ICER £0.04 per day saved). Colchicine was less costly and more effective in the complete case analysis (dominant; ICER -£812), but more costly and more effective in the concurrent randomisation analysis (ICER £1,331) and more costly and less effective among inhaled corticosteroid users (dominated). Favipiravir was generally associated with lower costs and faster recovery across most subgroups, although it was more costly and more effective in the concurrent randomisation analysis (ICER £38 per day saved) and among participants aged 18 to 64 years (ICER £7 per day saved). Ivermectin was consistently more costly and slower to recovery than usual care across all subgroup analyses.

## Discussion

This study evaluated the cost-effectiveness of six repurposed community-based treatments tested within a large adaptive platform trial. By embedding economic evaluation alongside the clinical evaluation, it provides complementary evidence on the costs and consequences of interventions in addition to their clinical effectiveness. Substantial variation in cost-effectiveness was observed across interventions and outcomes, highlighting the importance of economic evaluation alongside clinical evidence when making resource allocation decisions under uncertainty.

This study contributes to the literature by providing one of the first comprehensive economic evaluations of multiple repurposed treatments tested sequentially within a single adaptive community-based platform trial. More broadly, it demonstrates the feasibility of embedding economic evaluation within an adaptive platform trial, enabling cost-effectiveness evidence to be generated alongside clinical effectiveness across multiple interventions using a common analytical framework. Although conducted retrospectively, this study illustrates the additional insights that trial-based economic evaluation can provide by complementing clinical findings with evidence on the costs and consequences of interventions. Previous economic studies of community COVID-19 treatments have focused primarily on novel antivirals or monoclonal antibodies [20–28], while this analysis examines the broader economic implications of repurposing existing medicines under conditions of uncertainty. The findings therefore extend beyond COVID-19 and are relevant to future pandemic preparedness, where rapid decisions about funding and disinvestment must often be made before definitive evidence is available.

The use of trial-based data from a large, randomised platform trial is a key strength, enabling consistent comparison of multiple interventions using a common design and analytic framework. This demonstrates the practicality of incorporating economic evaluation within adaptive platform trials, even when analyses are completed after trial follow-up. Evaluating cost-effectiveness across more than one outcome further illustrates how conclusions may differ depending on decision objectives, such as accelerating recovery versus preventing severe events. Inclusion of indirect costs also provides a more comprehensive assessment of resource use than is typically reported in emergency evaluations.

Several limitations should be acknowledged. The trial was conducted largely before widespread vaccination and during periods of different variant predominance, which limits direct transferability of results to later stages of the pandemic. However, this context reflects the conditions under which many emergency funding decisions are made and therefore strengthens the relevance of the findings for understanding resource allocation under uncertainty. Longer-term analyses were constrained by lower response rates to follow-up resource use questionnaires, and health-related quality of life was not measured using EQ-5D as recommended by NICE [29], precluding estimation of cost per QALY. As a result, cost-effectiveness was assessed using alternative outcomes, and associated cost-effectiveness thresholds remain uncertain. In addition, inpatient care was a major cost driver and formed part of the composite effectiveness outcome, meaning ICERs may represent a lower bound and should be interpreted cautiously. Finally, conducting economic evaluations during a pandemic presents methodological challenges, including rapidly evolving standards of care, changing disease epidemiology, and the need to generate timely evidence while follow-up and resource-use data remain incomplete. These factors should be considered when interpreting economic evidence generated during public health emergencies.

## Conclusion

This study demonstrates how economic evaluation embedded within large adaptive platform trials can inform efficient allocation of health care resources during public health emergencies. By quantifying both favourable and unfavourable cost-effectiveness profiles, including for interventions that ultimately proved clinically ineffective, the analysis highlights the value of economic evidence for supporting timely disinvestment and reducing opportunity costs. These findings support the integration of prospective economic data collection into rapid trial platforms to strengthen decision-making and preparedness for future pandemics.

## Supplementary Information

Below is the link to the electronic supplementary material.

Supplementary Material 1

## Data Availability

Data can be shared with qualifying researchers who submit a proposal with a valuable research question as assessed by a committee formed from the Trial Management Group including senior statistical and clinical representation. A contract should be signed. Data sharing requests should be sent to the corresponding author.
