# Supplementary material for: Cost-effectiveness of community-based repurposed therapies for COVID-19 in the PRINCIPLE trial
Source: Cost Eff Resour Alloc. 2026 Aug 1;24:133. doi: 10.1186/s12962-026-00809-x (PMC13721731; doi:10.1186/s12962-026-00809-x)
Supplement: Supplementary file 1 [file 12962_2026_809_MOESM1_ESM.docx]

**Cost-Effectiveness of Community-Based Repurposed Therapies for COVID-19 in the PRINCIPLE Trial**

**Supplementary Material**

**Contents**

[ADDITIONAL METHODOLOGICAL DETAILS	3](#_Toc235176038)

[TABLES	4](#_Toc235176039)

[Table S1. Unit cost of resource items, in 2022/23£	4](#_Toc235176040)

[Table S2. Baseline characteristics of participants	7](#_Toc235176041)

[Table S3. Completion rate of resource use and outcomes in the primary analysis population	10](#_Toc235176042)

[Table S4. Mean (SE) resource use consumption per participant by treatment arm at each time-point (available case): Usual care vs. Azithromycin	11](#_Toc235176043)

[Table S5. Mean (SE) resource use consumption per participant by treatment arm at each time-point (available case): Usual care vs. Doxycycline	13](#_Toc235176044)

[Table S6. Mean (SE) resource use consumption per participant by treatment arm at each time-point (available case): Usual care vs. Budesonide	15](#_Toc235176045)

[Table S7. Mean (SE) resource use consumption per participant by treatment arm at each time-point (available case): Usual care vs. Colchicine	17](#_Toc235176046)

[Table S8. Mean (SE) resource use consumption per participant by treatment arm at each time-point (available case): Usual care vs. Favipiravir	19](#_Toc235176047)

[Table S9. Mean (SE) resource use consumption per participant by treatment arm at each time-point (available case): Usual care vs. Ivermectin	21](#_Toc235176048)

[Table S10. Mean cost of resource use consumption per participant by treatment arm at each time-point (available case), in 2022/23£: Usual care vs. Azithromycin	23](#_Toc235176049)

[Table S11. Mean cost of resource use consumption per participant by treatment arm at each time-point (available case), in 2022/23£: Usual care vs. Doxycycline	25](#_Toc235176050)

[Table S12. Mean cost of resource use consumption per participant by treatment arm at each time-point (available case), in 2022/23£: Usual care vs. Budesonide	27](#_Toc235176051)

[Table S13. Mean cost of resource use consumption per participant by treatment arm at each time-point (available case), in 2022/23£: Usual care vs. Colchicine	29](#_Toc235176052)

[Table S14. Mean cost of resource use consumption per participant by treatment arm at each time-point (available case), in 2022/23£: Usual care vs. Favipiravir	31](#_Toc235176053)

[Table S15. Mean cost of resource use consumption per participant by treatment arm at each time-point (available case), in 2022/23£: Usual care vs. Ivermectin	33](#_Toc235176054)

[Table S16. Mean outcome by treatment arm at each time-point (available case): Usual care vs. Azithromycin	35](#_Toc235176055)

[Table S17. Mean outcome by treatment arm at each time-point (available case): Usual care vs. Doxycycline	35](#_Toc235176056)

[Table S18. Mean outcome by treatment arm at each time-point (available case): Usual care vs. Budesonide	35](#_Toc235176057)

[Table S19. Mean outcome by treatment arm at each time-point (available case): Usual care vs. Colchicine	35](#_Toc235176058)

[Table S20. Mean outcome by treatment arm at each time-point (available case): Usual care vs. Favipiravir	36](#_Toc235176059)

[Table S21. Mean outcome by treatment arm at each time-point (available case): Usual care vs. Ivermectin	36](#_Toc235176060)

[Table S22. Sensitivity and subgroup analyses of the cost-effectiveness analysis in terms of incremental cost per hospitalisation or death avoided: Usual care vs. Azithromycin	37](#_Toc235176061)

[Table S23. Sensitivity and subgroup analyses of the cost-effectiveness analysis in terms of incremental cost per hospitalisation or death avoided: Usual care vs. Doxycycline	38](#_Toc235176062)

[Table S24. Sensitivity and subgroup analyses of the cost-effectiveness analysis in terms of incremental cost per hospitalisation or death avoided: Usual care vs. Budesonide	40](#_Toc235176063)

[Table S25. Sensitivity and subgroup analyses of the cost-effectiveness analysis in terms of incremental cost per hospitalisation or death avoided: Usual care vs. Colchicine	41](#_Toc235176064)

[Table S26. Sensitivity and subgroup analyses of the cost-effectiveness analysis in terms of incremental cost per hospitalisation or death avoided: Usual care vs. Favipiravir	42](#_Toc235176065)

[Table S27. Sensitivity and subgroup analyses of the cost-effectiveness analysis in terms of incremental cost per hospitalisation or death avoided: Usual care vs. Ivermectin	44](#_Toc235176066)

[Table S28. Sensitivity and subgroup analyses of the cost-effectiveness analysis in terms of incremental cost per day saved until recovery: Usual care vs. Azithromycin	45](#_Toc235176067)

[Table S29. Sensitivity and subgroup analyses of the cost-effectiveness analysis in terms of incremental cost per day saved until recovery: Usual care vs. Doxycycline	46](#_Toc235176068)

[Table S30. Sensitivity and subgroup analyses of the cost-effectiveness analysis in terms of incremental cost per day saved until recovery: Usual care vs. Budesonide	47](#_Toc235176069)

[Table S31. Sensitivity and subgroup analyses of the cost-effectiveness analysis in terms of incremental cost per day saved until recovery: Usual care vs. Colchicine	48](#_Toc235176070)

[Table S32. Sensitivity and subgroup analyses of the cost-effectiveness analysis in terms of incremental cost per day saved until recovery: Usual care vs. Favipiravir	49](#_Toc235176071)

[Table S33. Sensitivity and subgroup analyses of the cost-effectiveness analysis in terms of incremental cost per day saved until recovery: Usual care vs. Ivermectin	50](#_Toc235176072)

[HEALTH ECONOMIC ANALYSIS PLAN	52](#_Toc235176073)

# ADDITIONAL METHODOLOGICAL DETAILS

**Description of intervention arms**

Participants were randomised to the following intervention arms before the Alpha variant became dominant in the UK (from the week ending December 18, 2020 to the week ending May 15, 2021):

- Hydroxychloroquine between April 2, 2020 and November 30, 2020. Participants took 200 mg of hydroxychloroquine twice daily for three days. They are excluded from the analysis as it was stopped prematurely by the UK regulator.
- Azithromycin between May 23, 2020 and November 30, 2020. Participants took 500 mg of azithromycin once daily for three days.
- Doxycycline between July 24, 2020 and December 14, 2020. Participants took 200 mg doxycycline on the first day followed by 100 mg of doxycycline daily for another six days.

Participants were randomised to the following intervention arms when the Alpha variant became dominant in the UK (from the week ending December 18, 2020 to the week ending May 15, 2021):

- Inhaled budesonide between December 1, 2020 and March 31, 2021. Participants took 400 micrograms of budesonide daily (as two puffs bd) for 14 days.
- Colchicine between March 4, 2021 and May 26, 2021. Participants took 500 micrograms of colchicine once daily for 14 days.

Participants were randomised to the following intervention arms when the Delta variant and Omicron variant became dominant in the UK (from the week ending May, 22 2021 to the week ending December 19, 2021 and from the week ending December 23, 2021 to the week ending September, 5 2022 respectively):

- Favipiravir between April 8, 2021 and July 1, 2022. Participants took 18000 mg of favipiravir twice a day on the first day and 800 mg of favipiravir twice a day for another four days.
- Ivermectin between June 23, 2021 and July 1, 2022. Participants took one dose daily for three days at 300 micrograms/kg of their body weight.

# TABLES

## Table S1. Unit cost of resource items, in 2022/23£

| **Resource item** | **Unit** | **Unit cost** | **Reference** |
| --- | --- | --- | --- |
| Intervention |  |  |  |
| Azithromycin | per course | 1.05 | PCA 2022/23[1] |
| Doxycycline | per course | 0.96 | PCA 2022/23[1] |
| Inhaled budesonide | per course | 18.67 | PCA 2022/23[1] |
| Colchicine | per course | 0.42 | PCA 2022/23[1] |
| Favipiravir* | per course | 74 | [2,3] |
| Ivermectin | per course per kg | 7.785 | PCA 2022/23[1] |
| Antibiotic | per item | 5.70 | PCA 2022/23[1] |
| Amoxicillin | per item | 4.17 | PCA 2022/23[1] |
| Ampicillin | per item | 122.03 | PCA 2022/23[1] |
| Azithromycin | per item | 3.61 | PCA 2022/23[1] |
| Benzylpenicillin | per item | 5.81 | PCA 2022/23[1] |
| Cefalexin | per item | 2.34 | PCA 2022/23[1] |
| Ceftriaxone | per item | 28.90 | PCA 2022/23[1] |
| Ciprofloxacin | per item | 2.35 | PCA 2022/23[1] |
| Clarithromycin | per item | 7.30 | PCA 2022/23[1] |
| Clindamycin | per item | 6.58 | PCA 2022/23[1] |
| Co-amoxiclav | per item | 4.70 | PCA 2022/23[1] |
| Doxycycline | per item | 1.49 | PCA 2022/23[1] |
| Erythromycin | per item | 4.34 | PCA 2022/23[1] |
| Flucloxacillin | per item | 3.19 | PCA 2022/23[1] |
| Gentamicin | per item | 26.74 | PCA 2022/23[1] |
| Levofloxacin | per item | 24.95 | PCA 2022/23[1] |
| Metronidazole | per item | 1.26 | PCA 2022/23[1] |
| Nitrofurantoin | per item | 6.92 | PCA 2022/23[1] |
| Ofloxacin | per item | 25.55 | PCA 2022/23[1] |
| Phenoxymethylpenicillin | per item | 6.00 | PCA 2022/23[1] |
| Teicoplanin | per item | 80.55 | PCA 2022/23[1] |
| Trimethoprim | per item | 10.70 | PCA 2022/23[1] |
| Inhaled corticosteroid | per item | 5.05 | PCA 2022/23[1], weighted average of beclomethasone, budesonide, fluticasone, mometasone |
| Other medication |  |  |  |
| Paracetamol | per item | 3.13 | PCA 2022/23[1] |
| Cough mixture | per item | 4.89 | PCA 2022/23[1] |
| Ibuprofen | per item | 2.69 | PCA 2022/23[1] |
| Co-codamol | per item | 4.10 | PCA 2022/23[1] |
| Cold/flu tablets | per item | 6.85 | PCA 2022/23[1] |
| Throat lozenges | per item | 18.75 | PCA 2022/23[1] |
| Antihistamine | per item | 5.79 | PCA 2022/23[1] |
| Inhaler (not part of PRINCIPLE trial inhaler) | per item | 30.07 | PCA 2022/23[1] |
| Steroid nasal spray (e.g. Beconase) | per item | 2.93 | PCA 2022/23[1] |
| Anti-diarrheal medication (e.g. Immodium) | per item | 2.50 | PCA 2022/23[1] |
| Inpatient |  |  |  |
| Non-elective long stay | per episode | 4719 | PSSRU 2023[4] |
| Non-elective short stay | per episode | 857 | PSSRU 2023[4] |
| Oxygen* | per use | 232 | NHS Reference Cost 2021/22[5] |
| Mechanical ventilation* | per use | 458 | NHS Reference Cost 2021/22[5] |
| Critical care* | per day | 2293.83 | NHS Reference Cost 2021/22[5] |
| Primary care services |  |  |  |
| General practitioner | per consult | 55 | PSSRU 2023[4] |
| Other | per consult | 47 | PSSRU 2023[4], assumed to be the cost of a practice nurse |
| NHS111* | per call | 13.10 | [6] |
| Accident & Emergency | per consult | 67 | PSSRU 2023[4] |
| Outpatient service | per consult | 217 | PSSRU 2023[4] |
| Acute medicine* | per consult | 88 | NHS Reference Cost 2021/22[5] |
| Anticoagulant* | per consult | 73 | NHS Reference Cost 2021/22[5] |
| Audiology* | per consult | 135 | NHS Reference Cost 2021/22[5] |
| Breast* | per consult | 211 | NHS Reference Cost 2021/22[5] |
| Burns* | per consult | 153 | NHS Reference Cost 2021/22[5] |
| Cardiology* | per consult | 181 | NHS Reference Cost 2021/22[5] |
| Colorectal* | per consult | 139 | NHS Reference Cost 2021/22[5] |
| Cystic fibrosis* | per consult | 572 | NHS Reference Cost 2021/22[5] |
| Denta* | per consult | 204 | NHS Reference Cost 2021/22[5] |
| Dermatology* | per consult | 163 | NHS Reference Cost 2021/22[5] |
| Diabetes* | per consult | 196 | NHS Reference Cost 2021/22[5] |
| Diagnostic imaging* | per consult | 49 | NHS Reference Cost 2021/22[5] |
| Endocrinology* | per consult | 111 | NHS Reference Cost 2021/22[5] |
| Ear, Nose, and Throat* | per consult | 166 | NHS Reference Cost 2021/22[5] |
| Gastroenterology* | per consult | 159 | NHS Reference Cost 2021/22[5] |
| Gastrointestinal* | per consult | 182 | NHS Reference Cost 2021/22[5] |
| Gynaecology* | per consult | 194 | NHS Reference Cost 2021/22[5] |
| Haematology* | per consult | 208 | NHS Reference Cost 2021/22[5] |
| Haemophilia* | per consult | 549 | NHS Reference Cost 2021/22[5] |
| Hepatology* | per consult | 206 | NHS Reference Cost 2021/22[5] |
| Immunology* | per consult | 188 | NHS Reference Cost 2021/22[5] |
| Infectious diseases* | per consult | 175 | NHS Reference Cost 2021/22[5] |
| Mental health* | per consult | 316 | NHS Reference Cost 2021/22[5] |
| Neurology* | per consult | 229 | NHS Reference Cost 2021/22[5] |
| Neurosurgery* | per consult | 232 | NHS Reference Cost 2021/22[5] |
| Occupational therapy* | per consult | 113 | NHS Reference Cost 2021/22[5] |
| Oncology* | per consult | 220 | NHS Reference Cost 2021/22[5] |
| Ophthalmology* | per consult | 152 | NHS Reference Cost 2021/22[5] |
| Oral surgery* | per consult | 195 | NHS Reference Cost 2021/22[5] |
| Orthodontic* | per consult | 226 | NHS Reference Cost 2021/22[5] |
| Orthopaedic* | per consult | 168 | NHS Reference Cost 2021/22[5] |
| Orthotic* | per consult | 184 | NHS Reference Cost 2021/22[5] |
| Pain* | per consult | 218 | NHS Reference Cost 2021/22[5] |
| Palliative* | per consult | 209 | NHS Reference Cost 2021/22[5] |
| Pathology* | per consult | 95 | NHS Reference Cost 2021/22[5] |
| Pharmacy* | per consult | 302 | NHS Reference Cost 2021/22[5] |
| Phlebotomy* | per consult | 5 | NHS Reference Cost 2021/22[5] |
| Physiotherapy* | per consult | 107 | NHS Reference Cost 2021/22[5] |
| Podiatry* | per consult | 151 | NHS Reference Cost 2021/22[5] |
| Post-covid* | per consult | 217 | NHS Reference Cost 2021/22[5] |
| Psychiatry* | per consult | 561 | NHS Reference Cost 2021/22[5] |
| Psychology* | per consult | 271 | NHS Reference Cost 2021/22[5] |
| Pulmonary* | per consult | 62 | NHS Reference Cost 2021/22[5] |
| Rehabilitation* | per consult | 176 | NHS Reference Cost 2021/22[5] |
| Renal* | per consult | 211 | NHS Reference Cost 2021/22[5] |
| Respiratory* | per consult | 198 | NHS Reference Cost 2021/22[5] |
| Rheumatology* | per consult | 177 | NHS Reference Cost 2021/22[5] |
| Sexual health* | per consult | 66 | NHS Reference Cost 2021/22[5] |
| Speech and language therapy* | per consult | 201 | NHS Reference Cost 2021/22[5] |
| Spinal* | per consult | 211 | NHS Reference Cost 2021/22[5] |
| Sport* | per consult | 210 | NHS Reference Cost 2021/22[5] |
| Stroke* | per consult | 323 | NHS Reference Cost 2021/22[5] |
| Thoracic* | per consult | 259 | NHS Reference Cost 2021/22[5] |
| Transplant* | per consult | 416 | NHS Reference Cost 2021/22[5] |
| Urology* | per consult | 148 | NHS Reference Cost 2021/22[5] |
| Vascular* | per consult | 222 | NHS Reference Cost 2021/22[5] |
| Median wage |  |  |  |
| All | per week | 575 | ONS[7] |
| Men | per week | 666 | ONS[7] |
| Women | per week | 491 | ONS[7] |

*Adjusted to 2022/23£ prices.

References:

1. NHS Business Services Authority. Prescription Cost Analysis – England – 2022-23 [Internet]. 2023 [cited 2024 Oct 28]. https://www.nhsbsa.nhs.uk/statistical-collections/prescription-cost-analysis-england/prescription-cost-analysis-england-2022-23. Accessed 28 Oct 2024

2. Campbell & Cochrane Economics Methods Group, EPPI Centre, University College London. CCEMG - EPPI-Centre Cost Converter [Internet]. 2024 [cited 2024 Nov 20]. https://eppi.ioe.ac.uk/costconversion/. Accessed 20 Nov 2024

3. Subhi A, Shamy A, Hussein S, Jarrett J, Kozma S, Harfouche C, et al. Use of anti-viral therapies in hospitalised COVID-19 patients in the United Arab Emirates: a cost-effectiveness and health-care resource use analysis. BMC Health Serv Res. 2023;23:383. https://doi.org/10.1186/s12913-023-09376-w

4. Jones KC, Weatherly H, Birch S, Castelli A, Chalkley M, Dargan A, et al. Unit Costs of Health and Social Care 2023 Manual [Internet]. Kent; 2024. https://kar.kent.ac.uk/105685/. Accessed 30 Oct 2024

5. National Health Service. 2021/22 National Cost Collection Data Publication [Internet]. 2024 [cited 2024 May 7]. https://www.england.nhs.uk/publication/2021-22-national-cost-collection-data-publication/. Accessed 7 May 2024

6. Turner J, Knowles E, Simpson R, Sampson F, Dixon S, Long J, et al. Cost–consequences of introducing NHS 111 Online. NIHR Journals Library; 2021 [cited 2024 Nov 20]; https://www.ncbi.nlm.nih.gov/books/NBK575169/. Accessed 20 Nov 2024

7. Office for National Statistics. Employee earnings in the UK: 2023 [Internet]. 2023 [cited 2024 Oct 28]. https://www.ons.gov.uk/employmentandlabourmarket/peopleinwork/earningsandworkinghours/bulletins/annualsurveyofhoursandearnings/2023. Accessed 28 Oct 2024

## Table S2. Baseline characteristics of participants

|  | **Primary analysis population** | | | | **Concurrent analysis population*** | | | |
| --- | --- | --- | --- | --- | --- | --- | --- | --- |
|  | **Intervention** | | **Usual care** | | **Intervention** | | **Usual care** | |
| **Usual Care vs. Azithromycin** |  |  |  |  |  |  |  |  |
| Total number of participants | 186 |  | 236 |  | 186 |  | 225 |  |
| Mean age (SE) | 61.9 | (0.6) | 61.4 | (0.5) | 61.9 | (0.6) | 61.4 | (0.5) |
| Age category (%) |  |  |  |  |  |  |  |  |
| 50-64 | 108 | (58) | 151 | (64) | 108 | (58) | 143 | (64) |
| 65 and above | 78 | (42) | 85 | (36) | 78 | (42) | 82 | (36) |
| Sex (%) |  |  |  |  |  |  |  |  |
| Male | 91 | (49) | 109 | (46) | 91 | (49) | 105 | (47) |
| Female | 95 | (51) | 127 | (54) | 95 | (51) | 120 | (53) |
| Ethnicity (%) |  |  |  |  |  |  |  |  |
| White | 169 | (91) | 216 | (92) | 169 | (91) | 207 | (92) |
| Non-White | 17 | (9) | 20 | (8) | 17 | (9) | 18 | (8) |
| Vaccinated (%)** | 0 | (0) | 0 | (0) | 0 | (0) | 0 | (0) |
| Comorbidity (%) | 157 | (84) | 207 | (88) | 157 | (84) | 197 | (88) |
| Duration of symptoms before randomisation (%) |  |  |  |  |  |  |  |  |
| Less than 7 days | 75 | (40) | 96 | (41) | 75 | (40) | 87 | (39) |
| 7 days and more | 111 | (60) | 140 | (59) | 111 | (60) | 138 | (61) |
| Major symptoms at baseline (%) |  |  |  |  |  |  |  |  |
| No symptoms rated as 'major' | 143 | (77) | 198 | (84) | 43 | (23) | 36 | (16) |
| At least one symptom rated as 'major' | 43 | (23) | 38 | (16) | 143 | (77) | 189 | (84) |
| Current smoker (%) | 10 | (5) | 13 | (6) | 10 | (5) | 13 | (6) |
| **Usual Care vs. Doxycycline** |  |  |  |  |  |  |  |  |
| Total number of participants | 435 |  | 336 |  | 435 |  | 321 |  |
| Mean age (SE) | 61.9 | (0.4) | 62.2 | (0.4) | 61.9 | (0.4) | 62.3 | (0.4) |
| Age category (%) |  |  |  |  |  |  |  |  |
| 50-64 | 248 | (57) | 189 | (56) | 187 | (43) | 143 | (45) |
| 65 and above | 187 | (43) | 147 | (44) | 248 | (57) | 178 | (55) |
| Sex (%) |  |  |  |  |  |  |  |  |
| Male | 205 | (47) | 152 | (45) | 205 | (47) | 146 | (45) |
| Female | 230 | (53) | 184 | (55) | 230 | (53) | 175 | (55) |
| Ethnicity (%) |  |  |  |  |  |  |  |  |
| White | 400 | (92) | 309 | (92) | 400 | (92) | 296 | (92) |
| Non-White | 35 | (8) | 27 | (8) | 35 | (8) | 25 | (8) |
| Vaccinated (%)** | 0 | (0) | 0 | (0) | 0 | (0) | 0 | (0) |
| Comorbidity (%) | 372 | (86) | 285 | (85) | 372 | (86) | 271 | (84) |
| Duration of symptoms before randomisation (%) |  |  |  |  |  |  |  |  |
| Less than 7 days | 194 | (45) | 145 | (43) | 241 | (55) | 187 | (58) |
| 7 days and more | 241 | (55) | 191 | (57) | 194 | (45) | 134 | (42) |
| Major symptoms at baseline (%) |  |  |  |  |  |  |  |  |
| No symptoms rated as 'major' | 346 | (80) | 272 | (81) | 346 | (80) | 261 | (81) |
| At least one symptom rated as 'major' | 89 | (20) | 64 | (19) | 89 | (20) | 60 | (19) |
| Current smoker (%) | 25 | (6) | 17 | (5) | 25 | (6) | 15 | (5) |
| **Usual Care vs. Budesonide** |  |  |  |  |  |  |  |  |
| Total number of participants (%) | 787 |  | 1069 |  | 787 |  | 838 |  |
| Mean age (SE) | 64.6 | (0.3) | 63.7 | (0.2) | 64.6 | (0.3) | 64.4 | (0.3) |
| Age category (%) |  |  |  |  |  |  |  |  |
| 50-64 | 286 | (36) | 460 | (43) | 286 | (36) | 310 | (37) |
| 65 and above | 501 | (64) | 609 | (57) | 501 | (64) | 528 | (63) |
| Sex (%) |  |  |  |  |  |  |  |  |
| Male | 381 | (48) | 514 | (48) | 381 | (48) | 409 | (49) |
| Female | 406 | (52) | 555 | (52) | 406 | (52) | 429 | (51) |
| Ethnicity (%) |  |  |  |  |  |  |  |  |
| White | 734 | (93) | 999 | (93) | 734 | (93) | 785 | (94) |
| Non-White | 53 | (7) | 70 | (7) | 53 | (7) | 53 | (6) |
| Vaccinated (%) | 265 | (34) | 282 | (26) | 265 | (34) | 282 | (34) |
| Comorbidity (%) | 628 | (80) | 873 | (82) | 628 | (80) | 671 | (80) |
| Duration of symptoms before randomisation (%) |  |  |  |  |  |  |  |  |
| Less than 7 days | 357 | (45) | 493 | (46) | 357 | (45) | 395 | (47) |
| 7 days and more | 430 | (55) | 576 | (54) | 430 | (55) | 443 | (53) |
| Major symptoms at baseline (%) |  |  |  |  |  |  |  |  |
| No symptoms rated as 'major' | 559 | (71) | 797 | (75) | 559 | (71) | 603 | (72) |
| At least one symptom rated as 'major' | 228 | (29) | 272 | (25) | 228 | (29) | 235 | (28) |
| Current smoker (%) | 40 | (5) | 59 | (6) | 40 | (5) | 45 | (5) |
| **Usual Care vs. Colchicine** |  |  |  |  |  |  |  |  |
| Total number of participants (%) | 156 |  | 1145 |  | 156 |  | 133 |  |
| Mean age (SE) | 47.4 | (1) | 62.6 | (0.3) | 47.4 | (1) | 52.9 | (1.2) |
| Age category (%) |  |  |  |  |  |  |  |  |
| 18-64 | 132 | (85) | 528 | (46) | 24 | (15) | 32 | (24) |
| 65 and above | 24 | (15) | 617 | (54) | 132 | (85) | 101 | (76) |
| Sex (%) |  |  |  |  |  |  |  |  |
| Male | 78 | (50) | 543 | (47) | 78 | (50) | 53 | (40) |
| Female | 77 | (49) | 602 | (53) | 77 | (49) | 80 | (60) |
| Other | 1 | (1) | 0 | (0) | 1 | (1) | 0 | (0) |
| Ethnicity (%) |  |  |  |  |  |  |  |  |
| White | 143 | (92) | 1062 | (93) | 143 | (92) | 119 | (89) |
| Non-White | 13 | (8) | 83 | (7) | 13 | (8) | 14 | (11) |
| Vaccinated (%) | 113 | (72) | 332 | (29) | 113 | (72) | 104 | (78) |
| Comorbidity (%) | 153 | (98) | 947 | (83) | 153 | (98) | 125 | (94) |
| Duration of symptoms before randomisation (%) |  |  |  |  |  |  |  |  |
| Less than 7 days | 98 | (63) | 548 | (48) | 98 | (63) | 83 | (62) |
| 7 days and more | 58 | (37) | 597 | (52) | 58 | (37) | 50 | (38) |
| Major symptoms at baseline (%) |  |  |  |  |  |  |  |  |
| No symptoms rated as 'major' | 86 | (55) | 829 | (72) | 70 | (45) | 70 | (53) |
| At least one symptom rated as 'major' | 70 | (45) | 316 | (28) | 86 | (55) | 63 | (47) |
| Current smoker (%) | 19 | (12) | 68 | (6) | 19 | (12) | 18 | (14) |
| **Usual Care vs. Favipiravir** |  |  |  |  |  |  |  |  |
| Total number of participants (%) | 1829 |  | 3256 |  | 1829 |  | 1668 |  |
| Mean age (SE) | 51.8 | (0.3) | 55.6 | (0.2) | 51.8 | (0.3) | 52.2 | (0.3) |
| Age category (%) |  |  |  |  |  |  |  |  |
| 18-64 | 1524 | (83) | 2286 | (70) | 1524 | (83) | 1389 | (83) |
| 65 and above | 305 | (17) | 970 | (30) | 305 | (17) | 279 | (17) |
| Sex (%) |  |  |  |  |  |  |  |  |
| Male | 690 | (38) | 1350 | (41) | 690 | (38) | 619 | (37) |
| Female | 1136 | (62) | 1902 | (58) | 1136 | (62) | 1045 | (63) |
| Other | 1 | (0) | 3 | (0) | 1 | (0) | 3 | (0) |
| Ethnicity (%) |  |  |  |  |  |  |  |  |
| White | 1742 | (95) | 3098 | (95) | 1742 | (95) | 1608 | (96) |
| Non-White | 87 | (5) | 158 | (5) | 87 | (5) | 60 | (4) |
| Vaccinated (%) | 1755 | (96) | 2348 | (72) | 1755 | (96) | 1599 | (96) |
| Comorbidity (%) | 1228 | (67) | 2366 | (73) | 1228 | (67) | 1131 | (68) |
| Duration of symptoms before randomisation (%) |  |  |  |  |  |  |  |  |
| Less than 7 days | 1347 | (74) | 2087 | (64) | 1347 | (74) | 1227 | (74) |
| 7 days and more | 482 | (26) | 1169 | (36) | 482 | (26) | 441 | (26) |
| Major symptoms at baseline (%) |  |  |  |  |  |  |  |  |
| No symptoms rated as 'major' | 1193 | (65) | 2234 | (69) | 1193 | (65) | 1103 | (66) |
| At least one symptom rated as 'major' | 636 | (35) | 1022 | (31) | 636 | (35) | 565 | (34) |
| Current smoker (%) | 116 | (6) | 206 | (6) | 116 | (6) | 114 | (7) |
| **Usual Care vs. Ivermectin** |  |  |  |  |  |  |  |  |
| Total number of participants (%) | 2157 |  | 3256 |  | 2157 |  | 1806 |  |
| Mean age (SE) | 51.3 | (0.3) | 55.6 | (0.2) | 51.3 | (0.3) | 52.0 | (0.3) |
| Age category (%) |  |  |  |  |  |  |  |  |
| 18-64 | 1811 | (84) | 2286 | (70) | 1811 | (84) | 1506 | (83) |
| 65 and above | 346 | (16) | 970 | (30) | 346 | (16) | 300 | (17) |
| Sex (%) |  |  |  |  |  |  |  |  |
| Male | 885 | (41) | 1350 | (41) | 885 | (41) | 688 | (38) |
| Female | 1270 | (59) | 1902 | (58) | 1270 | (59) | 1117 | (62) |
| Other | 0 | (0) | 3 | (0) | 0 | (0) | 1 | (0) |
| Ethnicity (%) |  |  |  |  |  |  |  |  |
| White | 2077 | (96) | 3098 | (95) | 2077 | (96) | 1747 | (97) |
| Non-White | 80 | (4) | 158 | (5) | 80 | (4) | 59 | (3) |
| Vaccinated (%) | 2038 | (94) | 2348 | (72) | 2038 | (94) | 1720 | (95) |
| Comorbidity (%) | 1460 | (68) | 2366 | (73) | 1460 | (68) | 1221 | (68) |
| Duration of symptoms before randomisation (%) |  |  |  |  |  |  |  |  |
| Less than 7 days | 1556 | (72) | 2087 | (64) | 1556 | (72) | 1312 | (73) |
| 7 days and more | 601 | (28) | 1169 | (36) | 601 | (28) | 494 | (27) |
| Major symptoms at baseline (%) |  |  |  |  |  |  |  |  |
| No symptoms rated as 'major' | 1418 | (66) | 2234 | (69) | 739 | (34) | 608 | (34) |
| At least one symptom rated as 'major' | 739 | (34) | 1022 | (31) | 1418 | (66) | 1198 | (66) |
| Current smoker (%) | 135 | (6) | 206 | (6) | 135 | (6) | 116 | (6) |

*The number of participants in the Concurrent analysis population differed from the published papers because only those who tested positive with COVID-19 were included here. ** Vaccination status was not available. SE: standard error

## Table S3. Completion rate of resource use and outcomes in the primary analysis population

|  | **Total no. of participants** | **Costs** | | | | | | | | **Hospitalisation or death** | | | | | | | | **Time-to-recovery** | |
| --- | --- | --- | --- | --- | --- | --- | --- | --- | --- | --- | --- | --- | --- | --- | --- | --- | --- | --- | --- |
|  |  | **28-day** | | **3-month** | | **6-month** | | **12-month** | | **28-day** | | **3-month** | | **6-month** | | **12-month** | | **28-day** | |
|  |  | **n** | **%** | **n** | **%** | **n** | **%** | **n** | **%** | **n** | **%** | **n** | **%** | **n** | **%** | **n** | **%** | **n** | **%** |
| **UC vs. AZI** |  |  |  |  |  |  |  |  |  |  |  |  |  |  |  |  |  |  |  |
| UC | 236 | 236 | 100 | 0 | 0 | 0 | 0 | 91 | 38.6 | 236 | 100 | 0 | 0 | 0 | 0 | 91 | 38.6 | 236 | 100 |
| AZI | 186 | 186 | 100 | 0 | 0 | 0 | 0 | 86 | 46.2 | 186 | 100 | 0 | 0 | 0 | 0 | 86 | 46.2 | 186 | 100 |
| **UC vs. DOX** |  |  |  |  |  |  |  |  |  |  |  |  |  |  |  |  |  |  |  |
| UC | 336 | 336 | 100 | 0 | 0 | 73 | 21.7 | 150 | 44.6 | 336 | 100 | 2 | 0.6 | 75 | 22.3 | 152 | 45.2 | 336 | 100 |
| DOX | 435 | 435 | 100 | 0 | 0 | 102 | 23.4 | 221 | 50.8 | 435 | 100 | 7 | 1.6 | 109 | 25.1 | 228 | 52.4 | 435 | 100 |
| **UC vs. BUD** |  |  |  |  |  |  |  |  |  |  |  |  |  |  |  |  |  |  |  |
| UC | 1069 | 1069 | 100 | 163 | 15.2 | 539 | 50.4 | 601 | 56.2 | 1069 | 100 | 175 | 16.4 | 551 | 51.5 | 613 | 57.3 | 1068 | 99.9 |
| BUD | 787 | 787 | 100 | 158 | 20.1 | 530 | 67.3 | 498 | 63.3 | 787 | 100 | 165 | 21.0 | 537 | 68.2 | 505 | 64.2 | 786 | 99.9 |
| **UC vs. COL** |  |  |  |  |  |  |  |  |  |  |  |  |  |  |  |  |  |  |  |
| UC | 1145 | 1145 | 100 | 212 | 18.5 | 585 | 51.1 | 651 | 56.9 | 1145 | 100 | 225 | 19.7 | 598 | 52.2 | 664 | 58.0 | 1144 | 99.9 |
| COL | 156 | 156 | 100 | 80 | 51.3 | 84 | 53.8 | 120 | 76.9 | 156 | 100 | 80 | 51.3 | 84 | 53.8 | 120 | 76.9 | 156 | 100 |
| **UC vs. FAV** |  |  |  |  |  |  |  |  |  |  |  |  |  |  |  |  |  |  |  |
| UC | 3256 | 3256 | 100 | 1942 | 59.6 | 2289 | 70.3 | 2439 | 74.9 | 3256 | 100 | 1956 | 60.1 | 2303 | 70.7 | 2453 | 75.3 | 3256 | 100 |
| FAV | 1829 | 1829 | 100 | 1506 | 82.3 | 1503 | 82.2 | 1582 | 86.5 | 1829 | 100 | 1507 | 82.4 | 1504 | 82.2 | 1583 | 86.6 | 1829 | 100 |
| **UC vs. IVE** |  |  |  |  |  |  |  |  |  |  |  |  |  |  |  |  |  |  |  |
| UC | 3256 | 3256 | 100 | 1942 | 59.6 | 2289 | 70.3 | 2439 | 74.9 | 3256 | 100 | 1956 | 60.1 | 2303 | 70.7 | 2453 | 75.3 | 3256 | 100 |
| IVE | 2157 | 2157 | 100 | 1765 | 81.8 | 1750 | 81.1 | 1845 | 85.5 | 2157 | 100 | 1767 | 81.9 | 1752 | 81.2 | 1847 | 85.6 | 2157 | 100 |

AZI: Azithromycin, BUD: budesonide, COL: colchicine, DOX: doxycycline, FAV: favipiravir, IVE: ivermectin, UC: Usual care

## Table S4. Mean (SE) resource use consumption per participant by treatment arm at each time-point (available case): Usual care vs. Azithromycin

|  | **Azithromycin** | | **Usual care** | | **Mean difference** | **p-value** | **Lower 95%CI** | **Upper 95%CI** |
| --- | --- | --- | --- | --- | --- | --- | --- | --- |
|  | **(n=186)** | | **(n=236)** | |  |  |  |  |
|  | **Mean** | **SE** | **Mean** | **SE** |  |  |  |  |
| **Baseline to 28 days** |  |  |  |  |  |  |  |  |
| No. of days on: |  |  |  |  |  |  |  |  |
| Anti-diarrheal medication | 0.525 | 0.226 | 0.317 | 0.095 | 0.209 | 0.396 | -0.219 | 0.740 |
| Hay fever tablets (antihistamine) | 1.165 | 0.390 | 1.497 | 0.360 | -0.333 | 0.531 | -1.335 | 0.721 |
| Cold/flu tablets or sachets | 0.614 | 0.165 | 0.759 | 0.169 | -0.145 | 0.540 | -0.598 | 0.312 |
| Cough mixture | 1.076 | 0.301 | 1.276 | 0.274 | -0.200 | 0.623 | -1.004 | 0.595 |
| Co-codamol or codeine-based medication | 1.551 | 0.406 | 1.578 | 0.306 | -0.027 | 0.957 | -0.984 | 0.999 |
| Ibuprofen | 1.190 | 0.284 | 1.869 | 0.308 | -0.679 | 0.106 | -1.499 | 0.127 |
| Inhaler (exclude PRINCIPLE trial inhaler) | 4.146 | 0.674 | 5.915 | 0.686 | -1.769 | 0.067 | -3.679 | 0.076 |
| Paracetamol | 6.930 | 0.618 | 7.472 | 0.603 | -0.542 | 0.531 | -2.210 | 1.125 |
| Steroid nasal spray | 1.234 | 0.351 | 1.266 | 0.312 | -0.032 | 0.945 | -0.942 | 0.895 |
| Throat lozenges | 1.285 | 0.341 | 1.482 | 0.310 | -0.198 | 0.668 | -1.067 | 0.718 |
| No. of admissions/visits/consultations: |  |  |  |  |  |  |  |  |
| Inpatient | 0.118 | 0.028 | 0.165 | 0.030 | -0.047 | 0.256 | -0.130 | 0.035 |
| Intensive care unit | 0.011 | 0.008 | 0.008 | 0.006 | 0.002 | 0.814 | -0.017 | 0.023 |
| NHS111 | 0.124 | 0.031 | 0.102 | 0.030 | 0.022 | 0.611 | -0.061 | 0.107 |
| A&E | 0.075 | 0.022 | 0.123 | 0.024 | -0.048 | 0.144 | -0.113 | 0.018 |
| Outpatient | 0.102 | 0.067 | 0.055 | 0.020 | 0.047 | 0.502 | -0.058 | 0.208 |
| General practitioner | 1.183 | 0.137 | 1.237 | 0.123 | -0.054 | 0.767 | -0.409 | 0.296 |
| Other primary care services | 0.118 | 0.033 | 0.123 | 0.029 | -0.005 | 0.916 | -0.090 | 0.082 |
| No. of participants who: |  |  |  |  |  |  |  |  |
| Took antibiotics | 0.088 | 0.022 | 0.127 | 0.027 | -0.039 | 0.268 | -0.108 | 0.029 |
| Received oxygen while in hospital | 0.054 | 0.018 | 0.038 | 0.012 | 0.016 | 0.480 | -0.027 | 0.061 |
| Received mechanical ventilation while in hospital | 0.005 | 0.005 | 0.008 | 0.006 | -0.003 | 0.700 | -0.020 | 0.012 |
| Length of stay in hospital, in days | 0.618 | 0.238 | 1.144 | 0.440 | -0.526 | 0.294 | -1.592 | 0.359 |
| **29 days to 3 months** |  |  |  |  |  |  |  |  |
| No. of admissions/visits/consultations: |  |  |  |  |  |  |  |  |
| Inpatient | 0 | 0 | 0 | 0 | 0 | - | - | - |
| Intensive care unit | 0 | 0 | 0 | 0 | 0 | - | - | - |
| A&E | 0 | 0 | 0 | 0 | 0 | - | - | - |
| Outpatient | 0 | 0 | 0 | 0 | 0 | - | - | - |
| General practitioner | 0 | 0 | 0 | 0 | 0 | - | - | - |
| No. of participants who: |  |  |  |  |  |  |  |  |
| Received oxygen while in hospital | 0 | 0 | 0 | 0 | 0 | - | - | - |
| Received mechanical ventilation while in hospital | 0 | 0 | 0 | 0 | 0 | - | - | - |
| Length of stay in hospital, in days | 0 | 0 | 0 | 0 | 0 | - | - | - |
| Time off work, in days* | 0 | 0 | 0 | 0 | 0 | - | - | - |
| **3 months to 6 months** |  |  |  |  |  |  |  |  |
| No. of admissions/visits/consultations: |  |  |  |  |  |  |  |  |
| Inpatient | 0 | 0 | 0 | 0 | 0 | - | - | - |
| Intensive care unit | 0 | 0 | 0 | 0 | 0 | - | - | - |
| A&E | 0 | 0 | 0 | 0 | 0 | - | - | - |
| Outpatient | 0 | 0 | 0 | 0 | 0 | - | - | - |
| General practitioner | 0 | 0 | 0 | 0 | 0 | - | - | - |
| No. of participants who: |  |  |  |  |  |  |  |  |
| Received oxygen while in hospital | 0 | 0 | 0 | 0 | 0 | - | - | - |
| Received mechanical ventilation while in hospital | 0 | 0 | 0 | 0 | 0 | - | - | - |
| Length of stay in hospital, in days | 0 | 0 | 0 | 0 | 0 | - | - | - |
| Time off work, in days | 0 | 0 | 0 | 0 | 0 | - | - | - |
| **6 months to 12 months** |  |  |  |  |  |  |  |  |
| No. of admissions/visits/consultations: |  |  |  |  |  |  |  |  |
| Inpatient | 0.035 | 0.026 | 0.011 | 0.011 | 0.024 | 0.397 | -0.022 | 0.082 |
| Intensive care unit | 0 | 0 | 0 | 0 | 0 | - | - | - |
| A&E | 0.116 | 0.069 | 0.110 | 0.053 | 0.006 | 0.942 | -0.153 | 0.188 |
| Outpatient | 0.419 | 0.114 | 0.352 | 0.104 | 0.067 | 0.665 | -0.232 | 0.371 |
| General practitioner | 1.570 | 0.544 | 0.714 | 0.170 | 0.855 | 0.136 | -0.098 | 2.051 |
| No. of participants who: |  |  |  |  |  |  |  |  |
| Received oxygen while in hospital | 0.012 | 0.012 | 0 | 0 | 0.012 | 0.320 | 0.000 | 0.035 |
| Received mechanical ventilation while in hospital | 0 | 0 | 0 | 0 | 0 | - | - | - |
| Length of stay in hospital, in days | 0.221 | 0.209 | 0.132 | 0.132 | 0.089 | 0.720 | -0.372 | 0.640 |
| Time off work, in days | 42.143 | 13.980 | 89.600 | 28.223 | -47.457 | 0.183 | -104.147 | 6.086 |

*Time off work at 3-month was measured from baseline to 3-month. A&E: accident and emergency, CI: confidence interval, NHS: National Health Service, SE: standard error

## Table S5. Mean (SE) resource use consumption per participant by treatment arm at each time-point (available case): Usual care vs. Doxycycline

|  | **Doxycycline** | | **Usual care** | | **Mean difference** | **p-value** | **Lower 95%CI** | **Upper 95%CI** |
| --- | --- | --- | --- | --- | --- | --- | --- | --- |
|  | **(n=435)** | | **(n=336)** | |  |  |  |  |
|  | **Mean** | **SE** | **Mean** | **SE** |  |  |  |  |
| **Baseline to 28 days** |  |  |  |  |  |  |  |  |
| No. of days on: |  |  |  |  |  |  |  |  |
| Anti-diarrheal medication | 0.257 | 0.101 | 0.259 | 0.072 | -0.002 | 0.987 | -0.227 | 0.256 |
| Hay fever tablets (antihistamine) | 1.612 | 0.269 | 1.738 | 0.344 | -0.126 | 0.774 | -0.988 | 0.715 |
| Cold/flu tablets or sachets | 0.770 | 0.134 | 0.858 | 0.169 | -0.088 | 0.685 | -0.516 | 0.329 |
| Cough mixture | 1.003 | 0.158 | 1.479 | 0.243 | -0.476 | 0.101 | -1.051 | 0.076 |
| Co-codamol or codeine-based medication | 1.355 | 0.250 | 1.592 | 0.270 | -0.237 | 0.519 | -0.960 | 0.488 |
| Ibuprofen | 1.355 | 0.189 | 1.794 | 0.265 | -0.439 | 0.177 | -1.087 | 0.178 |
| Inhaler (exclude PRINCIPLE trial inhaler) | 5.751 | 0.487 | 5.748 | 0.568 | 0.003 | 0.997 | -1.464 | 1.423 |
| Paracetamol | 6.030 | 0.400 | 8.170 | 0.531 | -2.140 | 0.001 | -3.455 | -0.852 |
| Steroid nasal spray | 1.224 | 0.242 | 1.564 | 0.299 | -0.340 | 0.378 | -1.100 | 0.399 |
| Throat lozenges | 1.492 | 0.211 | 1.699 | 0.267 | -0.207 | 0.544 | -0.874 | 0.450 |
| No. of admissions/visits/consultations: |  |  |  |  |  |  |  |  |
| Inpatient | 0.152 | 0.021 | 0.190 | 0.027 | -0.039 | 0.252 | -0.106 | 0.025 |
| Intensive care unit | 0.007 | 0.004 | 0.012 | 0.006 | -0.005 | 0.483 | -0.019 | 0.008 |
| NHS111 | 0.087 | 0.022 | 0.098 | 0.025 | -0.011 | 0.741 | -0.075 | 0.053 |
| A&E | 0.094 | 0.015 | 0.131 | 0.022 | -0.037 | 0.166 | -0.089 | 0.014 |
| Outpatient | 0.051 | 0.013 | 0.042 | 0.014 | 0.009 | 0.649 | -0.030 | 0.047 |
| General practitioner | 1.101 | 0.082 | 1.143 | 0.096 | -0.042 | 0.741 | -0.295 | 0.201 |
| Other primary care services | 0.064 | 0.015 | 0.098 | 0.021 | -0.034 | 0.190 | -0.086 | 0.016 |
| No. of participants who: |  |  |  |  |  |  |  |  |
| Took antibiotics | 0.085 | 0.015 | 0.152 | 0.024 | -0.067 | 0.019 | -0.123 | -0.012 |
| Received oxygen while in hospital | 0.057 | 0.012 | 0.060 | 0.013 | -0.002 | 0.908 | -0.036 | 0.032 |
| Received mechanical ventilation while in hospital | 0.007 | 0.004 | 0.015 | 0.007 | -0.008 | 0.301 | -0.024 | 0.006 |
| Length of stay in hospital, in days | 0.676 | 0.143 | 1.161 | 0.330 | -0.485 | 0.178 | -1.246 | 0.139 |
| **29 days to 3 months** |  |  |  |  |  |  |  |  |
| No. of admissions/visits/consultations: |  |  |  |  |  |  |  |  |
| Inpatient | 0 | 0 | 0 | 0 | 0 | - | - | - |
| Intensive care unit | 0 | 0 | 0 | 0 | 0 | - | - | - |
| A&E | 0 | 0 | 0 | 0 | 0 | - | - | - |
| Outpatient | 0 | 0 | 0 | 0 | 0 | - | - | - |
| General practitioner | 0 | 0 | 0 | 0 | 0 | - | - | - |
| No. of participants who: |  |  |  |  |  |  |  |  |
| Received oxygen while in hospital | 0 | 0 | 0 | 0 | 0 | - | - | - |
| Received mechanical ventilation while in hospital | 0 | 0 | 0 | 0 | 0 | - | - | - |
| Length of stay in hospital, in days | 0 | 0 | 0 | 0 | 0 | - | - | - |
| Time off work, in days* | 0 | 0 | 0 | 0 | 0 | - | - | - |
| **3 months to 6 months** |  |  |  |  |  |  |  |  |
| No. of admissions/visits/consultations: |  |  |  |  |  |  |  |  |
| Inpatient | 0.010 | 0.010 | 0.055 | 0.033 | -0.045 | 0.197 | -0.123 | 0.010 |
| Intensive care unit | 0 | 0 | 0 | 0 | 0 | - | - | - |
| A&E | 0.069 | 0.029 | 0.014 | 0.014 | 0.055 | 0.087 | -0.004 | 0.118 |
| Outpatient | 0.275 | 0.115 | 0.151 | 0.077 | 0.124 | 0.373 | -0.137 | 0.410 |
| General practitioner | 0.755 | 0.160 | 0.781 | 0.249 | -0.026 | 0.930 | -0.648 | 0.507 |
| No. of participants who: |  |  |  |  |  |  |  |  |
| Received oxygen while in hospital | 0.010 | 0.010 | 0.055 | 0.033 | -0.045 | 0.197 | -0.123 | 0.010 |
| Received mechanical ventilation while in hospital | 0 | 0 | 0 | 0 | 0 | - | - | - |
| Length of stay in hospital, in days | 0.059 | 0.059 | 0.425 | 0.294 | -0.366 | 0.227 | -1.051 | 0.104 |
| Time off work, in days | 53.125 | 17.765 | 39.333 | 16.593 | 13.792 | 0.581 | -30.918 | 57.668 |
| **6 months to 12 months** |  |  |  |  |  |  |  |  |
| No. of admissions/visits/consultations: |  |  |  |  |  |  |  |  |
| Inpatient | 0.036 | 0.016 | 0.033 | 0.022 | 0.003 | 0.915 | -0.055 | 0.052 |
| Intensive care unit | 0.005 | 0.005 | 0 | 0 | 0.005 | 0.318 | 0.000 | 0.014 |
| A&E | 0.068 | 0.019 | 0.073 | 0.033 | -0.005 | 0.886 | -0.084 | 0.064 |
| Outpatient | 0.262 | 0.068 | 0.320 | 0.074 | -0.058 | 0.568 | -0.256 | 0.135 |
| General practitioner | 0.855 | 0.137 | 0.620 | 0.118 | 0.235 | 0.194 | -0.116 | 0.587 |
| No. of participants who: |  |  |  |  |  |  |  |  |
| Received oxygen while in hospital | 0.009 | 0.006 | 0.027 | 0.021 | -0.018 | 0.424 | -0.069 | 0.016 |
| Received mechanical ventilation while in hospital | 0 | 0 | 0 | 0 | 0 | - | - | - |
| Length of stay in hospital, in days | 0.059 | 0.035 | 0.287 | 0.209 | -0.228 | 0.284 | -0.723 | 0.077 |
| Time off work, in days | 84.500 | 19.570 | 89.600 | 28.223 | -5.100 | 0.886 | -66.126 | 57.613 |

*Time off work at 3-month was measured from baseline to 3-month. A&E: accident and emergency, CI: confidence interval, NHS: National Health Service, SE: standard error

## Table S6. Mean (SE) resource use consumption per participant by treatment arm at each time-point (available case): Usual care vs. Budesonide

|  | **Budesonide** | | **Usual care** | | **Mean difference** | **p-value** | **Lower 95%CI** | **Upper 95%CI** |
| --- | --- | --- | --- | --- | --- | --- | --- | --- |
|  | **(n=787)** | | **(n=1069)** | |  |  |  |  |
|  | **Mean** | **SE** | **Mean** | **SE** |  |  |  |  |
| **Baseline to 28 days** |  |  |  |  |  |  |  |  |
| No. of days on: |  |  |  |  |  |  |  |  |
| Anti-diarrheal medication | 0.241 | 0.057 | 0.328 | 0.069 | -0.087 | 0.330 | -0.265 | 0.089 |
| Hay fever tablets (antihistamine) | 0.865 | 0.164 | 1.582 | 0.191 | -0.717 | 0.004 | -1.210 | -0.236 |
| Cold/flu tablets or sachets | 0.526 | 0.079 | 0.930 | 0.107 | -0.404 | 0.003 | -0.666 | -0.145 |
| Cough mixture | 1.051 | 0.128 | 1.594 | 0.149 | -0.542 | 0.006 | -0.934 | -0.163 |
| Co-codamol or codeine-based medication | 1.367 | 0.178 | 1.569 | 0.165 | -0.201 | 0.407 | -0.675 | 0.282 |
| Ibuprofen | 1.555 | 0.167 | 1.833 | 0.160 | -0.278 | 0.230 | -0.734 | 0.176 |
| Inhaler (exclude PRINCIPLE trial inhaler) | 4.822 | 0.292 | 3.103 | 0.256 | 1.720 | <0.001 | 0.962 | 2.485 |
| Paracetamol | 6.165 | 0.304 | 7.914 | 0.292 | -1.749 | <0.001 | -2.589 | -0.927 |
| Steroid nasal spray | 0.848 | 0.136 | 1.081 | 0.151 | -0.233 | 0.252 | -0.622 | 0.169 |
| Throat lozenges | 1.369 | 0.154 | 1.976 | 0.170 | -0.607 | 0.008 | -1.060 | -0.159 |
| No. of admissions/visits/consultations: |  |  |  |  |  |  |  |  |
| Inpatient | 0.187 | 0.017 | 0.199 | 0.015 | -0.012 | 0.586 | -0.055 | 0.033 |
| Intensive care unit | 0.018 | 0.005 | 0.027 | 0.005 | -0.009 | 0.210 | -0.024 | 0.005 |
| NHS111 | 0.118 | 0.019 | 0.109 | 0.015 | 0.010 | 0.694 | -0.037 | 0.058 |
| A&E | 0.046 | 0.010 | 0.076 | 0.011 | -0.030 | 0.046 | -0.059 | -0.001 |
| Outpatient | 0.039 | 0.008 | 0.041 | 0.007 | -0.002 | 0.874 | -0.023 | 0.020 |
| General practitioner | 1.107 | 0.063 | 1.184 | 0.054 | -0.078 | 0.350 | -0.242 | 0.089 |
| Other primary care services | 0.044 | 0.009 | 0.062 | 0.009 | -0.017 | 0.177 | -0.042 | 0.008 |
| No. of participants who: |  |  |  |  |  |  |  |  |
| Took antibiotics | 0.118 | 0.014 | 0.149 | 0.014 | -0.032 | 0.106 | -0.070 | 0.007 |
| Received oxygen while in hospital | 0.064 | 0.010 | 0.080 | 0.009 | -0.017 | 0.205 | -0.043 | 0.009 |
| Received mechanical ventilation while in hospital | 0.011 | 0.004 | 0.019 | 0.004 | -0.007 | 0.229 | -0.019 | 0.005 |
| Length of stay in hospital, in days | 0.949 | 0.189 | 1.233 | 0.171 | -0.284 | 0.266 | -0.784 | 0.219 |
| **29 days to 3 months** |  |  |  |  |  |  |  |  |
| No. of admissions/visits/consultations: |  |  |  |  |  |  |  |  |
| Inpatient | 0 | 0 | 0.025 | 0.012 | -0.025 | 0.045 | -0.049 | -0.006 |
| Intensive care unit | 0 | 0 | 0.025 | 0.015 | -0.025 | 0.103 | -0.055 | 0 |
| A&E | 0.051 | 0.051 | 0.025 | 0.015 | 0.026 | 0.622 | -0.049 | 0.146 |
| Outpatient | 0.146 | 0.057 | 0.209 | 0.074 | -0.063 | 0.502 | -0.259 | 0.112 |
| General practitioner | 0.449 | 0.112 | 0.564 | 0.115 | -0.115 | 0.474 | -0.432 | 0.205 |
| No. of participants who: |  |  |  |  |  |  |  |  |
| Received oxygen while in hospital | 0 | 0 | 0.025 | 0.012 | -0.025 | 0.045 | -0.049 | -0.006 |
| Received mechanical ventilation while in hospital | 0 | 0 | 0 | 0 | 0 | - | - | - |
| Length of stay in hospital, in days | 0 | 0 | 0.325 | 0.231 | -0.325 | 0.161 | -0.859 | -0.006 |
| Time off work, in days* | 20 | 4.763 | 34.857 | 5.527 | -14.857 | 0.053 | -28.286 | -0.513 |
| **3 months to 6 months** |  |  |  |  |  |  |  |  |
| No. of admissions/visits/consultations: |  |  |  |  |  |  |  |  |
| Inpatient | 0.013 | 0.006 | 0.024 | 0.008 | -0.011 | 0.266 | -0.031 | 0.008 |
| Intensive care unit | 0.004 | 0.004 | 0.002 | 0.002 | 0.002 | 0.648 | -0.006 | 0.011 |
| A&E | 0.070 | 0.025 | 0.030 | 0.008 | 0.040 | 0.131 | -0.003 | 0.100 |
| Outpatient | 0.164 | 0.028 | 0.165 | 0.027 | -0.001 | 0.980 | -0.076 | 0.074 |
| General practitioner | 0.555 | 0.070 | 0.557 | 0.064 | -0.002 | 0.984 | -0.182 | 0.183 |
| No. of participants who: |  |  |  |  |  |  |  |  |
| Received oxygen while in hospital | 0.006 | 0.003 | 0.013 | 0.006 | -0.007 | 0.292 | -0.022 | 0.006 |
| Received mechanical ventilation while in hospital | 0 | 0 | 0 | 0 | 0 | - | - | - |
| Length of stay in hospital, in days | 0.047 | 0.029 | 0.212 | 0.088 | -0.164 | 0.075 | -0.354 | -0.001 |
| Time off work, in days | 57.903 | 9.818 | 53.656 | 8.499 | 4.247 | 0.745 | -20.757 | 29.585 |
| **6 months to 12 months** |  |  |  |  |  |  |  |  |
| No. of admissions/visits/consultations: |  |  |  |  |  |  |  |  |
| Inpatient | 0.026 | 0.012 | 0.028 | 0.010 | -0.002 | 0.890 | -0.032 | 0.030 |
| Intensive care unit | 0.002 | 0.002 | 0 | 0 | 0.002 | 0.318 | 0.000 | 0.006 |
| A&E | 0.072 | 0.025 | 0.067 | 0.016 | 0.006 | 0.848 | -0.050 | 0.069 |
| Outpatient | 0.227 | 0.041 | 0.300 | 0.038 | -0.073 | 0.192 | -0.180 | 0.037 |
| General practitioner | 0.558 | 0.085 | 0.566 | 0.060 | -0.007 | 0.943 | -0.206 | 0.201 |
| No. of participants who: |  |  |  |  |  |  |  |  |
| Received oxygen while in hospital | 0.012 | 0.009 | 0.008 | 0.006 | 0.004 | 0.713 | -0.014 | 0.026 |
| Received mechanical ventilation while in hospital | 0.002 | 0.002 | 0 | 0 | 0.002 | 0.318 | 0.000 | 0.006 |
| Length of stay in hospital, in days | 0.179 | 0.110 | 0.211 | 0.134 | -0.033 | 0.851 | -0.390 | 0.292 |
| Time off work, in days | 59.773 | 12.928 | 59.771 | 14.653 | 0.001 | 1.000 | -38.821 | 37.192 |

*Time off work at 3-month was measured from baseline to 3-month. A&E: accident and emergency, CI: confidence interval, NHS: National Health Service, SE: standard error

## Table S7. Mean (SE) resource use consumption per participant by treatment arm at each time-point (available case): Usual care vs. Colchicine

|  | **Colchicine** | | **Usual care** | | **Mean difference** | **p-value** | **Lower 95%CI** | **Upper 95%CI** |
| --- | --- | --- | --- | --- | --- | --- | --- | --- |
|  | **(n=156)** | | **(n=1145)** | |  |  |  |  |
|  | **Mean** | **SE** | **Mean** | **SE** |  |  |  |  |
| **Baseline to 28 days** |  |  |  |  |  |  |  |  |
| No. of days on: |  |  |  |  |  |  |  |  |
| Anti-diarrheal medication | 0.242 | 0.146 | 0.314 | 0.064 | -0.072 | 0.654 | -0.337 | 0.287 |
| Hay fever tablets (antihistamine) | 2.798 | 0.653 | 1.701 | 0.192 | 1.097 | 0.109 | -0.159 | 2.485 |
| Cold/flu tablets or sachets | 0.565 | 0.242 | 0.938 | 0.102 | -0.373 | 0.158 | -0.808 | 0.212 |
| Cough mixture | 0.702 | 0.216 | 1.627 | 0.145 | -0.926 | <0.001 | -1.416 | -0.397 |
| Co-codamol or codeine-based medication | 1.798 | 0.480 | 1.634 | 0.163 | 0.164 | 0.746 | -0.746 | 1.210 |
| Ibuprofen | 2.056 | 0.503 | 1.838 | 0.153 | 0.218 | 0.678 | -0.764 | 1.297 |
| Inhaler (exclude PRINCIPLE trial inhaler) | 7.089 | 1.002 | 3.292 | 0.257 | 3.796 | <0.001 | 1.840 | 5.876 |
| Paracetamol | 6.524 | 0.768 | 7.975 | 0.283 | -1.451 | 0.078 | -3.015 | 0.196 |
| Steroid nasal spray | 1.403 | 0.453 | 1.097 | 0.147 | 0.306 | 0.522 | -0.530 | 1.301 |
| Throat lozenges | 0.806 | 0.201 | 1.953 | 0.162 | -1.147 | <0.001 | -1.629 | -0.626 |
| No. of admissions/visits/consultations: |  |  |  |  |  |  |  |  |
| Inpatient | 0.135 | 0.030 | 0.191 | 0.014 | -0.057 | 0.091 | -0.119 | 0.012 |
| Intensive care unit | 0 | 0 | 0.025 | 0.005 | -0.025 | <0.001 | -0.036 | -0.016 |
| NHS111 | 0.038 | 0.015 | 0.108 | 0.015 | -0.070 | 0.001 | -0.110 | -0.026 |
| A&E | 0.019 | 0.014 | 0.071 | 0.011 | -0.052 | 0.004 | -0.083 | -0.013 |
| Outpatient | 0.032 | 0.021 | 0.041 | 0.007 | -0.009 | 0.688 | -0.045 | 0.042 |
| General practitioner | 0.801 | 0.112 | 1.173 | 0.054 | -0.372 | 0.003 | -0.611 | -0.118 |
| Other primary care services | 0.026 | 0.013 | 0.064 | 0.009 | -0.038 | 0.014 | -0.066 | -0.006 |
| No. of participants who: |  |  |  |  |  |  |  |  |
| Took antibiotics | 0.101 | 0.031 | 0.153 | 0.014 | -0.052 | 0.122 | -0.114 | 0.016 |
| Received oxygen while in hospital | 0.026 | 0.013 | 0.075 | 0.009 | -0.049 | 0.001 | -0.078 | -0.017 |
| Received mechanical ventilation while in hospital | 0 | 0 | 0.017 | 0.004 | -0.017 | <0.001 | -0.026 | -0.010 |
| Length of stay in hospital, in days | 0.391 | 0.184 | 1.158 | 0.160 | -0.767 | 0.002 | -1.220 | -0.255 |
| **29 days to 3 months** |  |  |  |  |  |  |  |  |
| No. of admissions/visits/consultations: |  |  |  |  |  |  |  |  |
| Inpatient | 0 | 0 | 0.033 | 0.014 | -0.033 | 0.019 | -0.061 | -0.009 |
| Intensive care unit | 0 | 0 | 0.019 | 0.012 | -0.019 | 0.103 | -0.047 | 0.000 |
| A&E | 0.050 | 0.030 | 0.028 | 0.015 | 0.022 | 0.521 | -0.036 | 0.095 |
| Outpatient | 0.150 | 0.054 | 0.203 | 0.061 | -0.053 | 0.516 | -0.213 | 0.102 |
| General practitioner | 0.500 | 0.155 | 0.524 | 0.097 | -0.024 | 0.898 | -0.361 | 0.347 |
| No. of participants who: |  |  |  |  |  |  |  |  |
| Received oxygen while in hospital | 0 | 0 | 0.028 | 0.013 | -0.028 | 0.034 | -0.057 | -0.005 |
| Received mechanical ventilation while in hospital | 0 | 0 | 0 | 0 | 0 | - | - | - |
| Length of stay in hospital, in days | 0 | 0 | 0.321 | 0.186 | -0.321 | 0.085 | -0.750 | -0.047 |
| Time off work, in days* | 42.889 | 12.812 | 31.158 | 4.384 | 11.731 | 0.407 | -11.772 | 37.457 |
| **3 months to 6 months** |  |  |  |  |  |  |  |  |
| No. of admissions/visits/consultations: |  |  |  |  |  |  |  |  |
| Inpatient | 0.036 | 0.036 | 0.026 | 0.008 | 0.010 | 0.784 | -0.039 | 0.092 |
| Intensive care unit | 0 | 0 | 0.002 | 0.002 | -0.002 | 0.318 | -0.005 | 0.000 |
| A&E | 0.060 | 0.039 | 0.031 | 0.008 | 0.029 | 0.475 | -0.032 | 0.121 |
| Outpatient | 0.024 | 0.017 | 0.166 | 0.026 | -0.142 | <0.001 | -0.203 | -0.081 |
| General practitioner | 0.500 | 0.266 | 0.542 | 0.061 | -0.042 | 0.878 | -0.474 | 0.564 |
| No. of participants who: |  |  |  |  |  |  |  |  |
| Received oxygen while in hospital | 0.000 | 0.000 | 0.015 | 0.007 | -0.015 | 0.020 | -0.029 | -0.003 |
| Received mechanical ventilation while in hospital | 0 | 0 | 0 | 0 | 0 | - | - | - |
| Length of stay in hospital, in days | 0.107 | 0.107 | 0.224 | 0.086 | -0.117 | 0.395 | -0.368 | 0.180 |
| Time off work, in days | 34.200 | 21.823 | 50.947 | 7.777 | -16.747 | 0.502 | -51.227 | 29.674 |
| **6 months to 12 months** |  |  |  |  |  |  |  |  |
| No. of admissions/visits/consultations: |  |  |  |  |  |  |  |  |
| Inpatient | 0.058 | 0.043 | 0.031 | 0.011 | 0.028 | 0.535 | -0.037 | 0.128 |
| Intensive care unit | 0 | 0 | 0 | 0 | 0 | - | - | - |
| A&E | 0.042 | 0.022 | 0.066 | 0.016 | -0.024 | 0.363 | -0.072 | 0.030 |
| Outpatient | 0.275 | 0.171 | 0.310 | 0.042 | -0.035 | 0.841 | -0.292 | 0.356 |
| General practitioner | 0.617 | 0.188 | 0.579 | 0.060 | 0.038 | 0.849 | -0.305 | 0.447 |
| No. of participants who: |  |  |  |  |  |  |  |  |
| Received oxygen while in hospital | 0.008 | 0.008 | 0.011 | 0.006 | -0.002 | 0.813 | -0.020 | 0.019 |
| Received mechanical ventilation while in hospital | 0 | 0 | 0 | 0 | 0 | - | - | - |
| Length of stay in hospital, in days | 0.067 | 0.048 | 0.198 | 0.124 | -0.131 | 0.323 | -0.427 | 0.079 |
| Time off work, in days | 68.600 | 25.508 | 60.634 | 13.040 | 7.966 | 0.785 | -41.930 | 65.062 |

*Time off work at 3-month was measured from baseline to 3-month. A&E: accident and emergency, CI: confidence interval, NHS: National Health Service, SE: standard error

## Table S8. Mean (SE) resource use consumption per participant by treatment arm at each time-point (available case): Usual care vs. Favipiravir

|  | **Favipiravir** | | **Usual care** | | **Mean difference** | **p-value** | **Lower 95%CI** | **Upper 95%CI** |
| --- | --- | --- | --- | --- | --- | --- | --- | --- |
|  | **(n=1829)** | | **(n=3256)** | |  |  |  |  |
|  | **Mean** | **SE** | **Mean** | **SE** |  |  |  |  |
| **Baseline to 28 days** |  |  |  |  |  |  |  |  |
| No. of days on: |  |  |  |  |  |  |  |  |
| Anti-diarrheal medication | 0.228 | 0.040 | 0.262 | 0.032 | -0.034 | 0.508 | -0.135 | 0.068 |
| Hay fever tablets (antihistamine) | 3.437 | 0.197 | 3.182 | 0.147 | 0.254 | 0.300 | -0.221 | 0.733 |
| Cold/flu tablets or sachets | 0.781 | 0.060 | 1.206 | 0.062 | -0.425 | <0.001 | -0.595 | -0.254 |
| Cough mixture | 0.830 | 0.071 | 1.539 | 0.079 | -0.709 | <0.001 | -0.921 | -0.500 |
| Co-codamol or codeine-based medication | 1.372 | 0.122 | 1.619 | 0.096 | -0.247 | 0.111 | -0.553 | 0.058 |
| Ibuprofen | 2.250 | 0.121 | 2.594 | 0.101 | -0.344 | 0.029 | -0.639 | -0.036 |
| Inhaler (exclude PRINCIPLE trial inhaler) | 4.691 | 0.240 | 4.554 | 0.179 | 0.137 | 0.646 | -0.447 | 0.715 |
| Paracetamol | 5.259 | 0.177 | 7.780 | 0.159 | -2.521 | <0.001 | -2.975 | -2.042 |
| Steroid nasal spray | 1.535 | 0.136 | 1.659 | 0.106 | -0.124 | 0.472 | -0.452 | 0.217 |
| Throat lozenges | 1.470 | 0.096 | 2.119 | 0.093 | -0.650 | <0.001 | -0.909 | -0.384 |
| No. of admissions/visits/consultations: |  |  |  |  |  |  |  |  |
| Inpatient | 0.084 | 0.008 | 0.111 | 0.006 | -0.027 | 0.008 | -0.047 | -0.007 |
| Intensive care unit | 0 | 0 | 0.010 | 0.002 | -0.010 | <0.001 | -0.013 | -0.006 |
| NHS111 | 0.021 | 0.006 | 0.053 | 0.006 | -0.032 | <0.001 | -0.048 | -0.015 |
| A&E | 0.010 | 0.003 | 0.032 | 0.004 | -0.022 | <0.001 | -0.032 | -0.012 |
| Outpatient | 0.062 | 0.008 | 0.059 | 0.006 | 0.003 | 0.752 | -0.015 | 0.022 |
| General practitioner | 0.654 | 0.029 | 0.837 | 0.028 | -0.183 | <0.001 | -0.263 | -0.106 |
| Other primary care services | 0.010 | 0.003 | 0.029 | 0.004 | -0.018 | <0.001 | -0.028 | -0.009 |
| No. of participants who: |  |  |  |  |  |  |  |  |
| Took antibiotics | 0.164 | 0.011 | 0.159 | 0.009 | 0.004 | 0.763 | -0.023 | 0.033 |
| Received oxygen while in hospital | 0.010 | 0.003 | 0.030 | 0.003 | -0.020 | <0.001 | -0.028 | -0.012 |
| Received mechanical ventilation while in hospital | 0 | 0 | 0.006 | 0.001 | -0.006 | <0.001 | -0.010 | -0.004 |
| Length of stay in hospital, in days | 0.072 | 0.017 | 0.453 | 0.061 | -0.381 | <0.001 | -0.508 | -0.261 |
| **29 days to 3 months** |  |  |  |  |  |  |  |  |
| No. of admissions/visits/consultations: |  |  |  |  |  |  |  |  |
| Inpatient | 0.009 | 0.003 | 0.011 | 0.003 | -0.002 | 0.574 | -0.010 | 0.005 |
| Intensive care unit | 0.001 | 0.001 | 0.003 | 0.001 | -0.002 | 0.207 | -0.005 | 0.001 |
| A&E | 0.028 | 0.005 | 0.026 | 0.005 | 0.002 | 0.763 | -0.012 | 0.016 |
| Outpatient | 0.067 | 0.011 | 0.095 | 0.012 | -0.028 | 0.080 | -0.059 | 0.004 |
| General practitioner | 0.307 | 0.031 | 0.377 | 0.026 | -0.070 | 0.082 | -0.148 | 0.009 |
| No. of participants who: |  |  |  |  |  |  |  |  |
| Received oxygen while in hospital | 0.002 | 0.001 | 0.004 | 0.002 | -0.002 | 0.285 | -0.006 | 0.002 |
| Received mechanical ventilation while in hospital | 0.001 | 0.001 | 0 | 0 | 0.001 | 0.317 | 0.000 | 0.002 |
| Length of stay in hospital, in days | 0.028 | 0.012 | 0.061 | 0.022 | -0.033 | 0.189 | -0.087 | 0.012 |
| Time off work, in days* | 24.352 | 2.659 | 22.724 | 1.493 | 1.628 | 0.594 | -3.888 | 7.925 |
| **3 months to 6 months** |  |  |  |  |  |  |  |  |
| No. of admissions/visits/consultations: |  |  |  |  |  |  |  |  |
| Inpatient | 0.005 | 0.002 | 0.014 | 0.003 | -0.008 | 0.023 | -0.015 | -0.001 |
| Intensive care unit | 0 | 0 | 0.0004 | 0.0004 | -0.0004 | 0.317 | -0.001 | 0.000 |
| A&E | 0.027 | 0.008 | 0.026 | 0.005 | 0.0004 | 0.964 | -0.017 | 0.018 |
| Outpatient | 0.103 | 0.015 | 0.115 | 0.012 | -0.012 | 0.518 | -0.049 | 0.025 |
| General practitioner | 0.271 | 0.028 | 0.343 | 0.024 | -0.072 | 0.051 | -0.143 | 0.004 |
| No. of participants who: |  |  |  |  |  |  |  |  |
| Received oxygen while in hospital | 0.002 | 0.001 | 0.005 | 0.002 | -0.003 | 0.172 | -0.008 | 0.001 |
| Received mechanical ventilation while in hospital | 0 | 0 | 0 | 0 | 0 | - | - | - |
| Length of stay in hospital, in days | 0.029 | 0.017 | 0.082 | 0.024 | -0.052 | 0.073 | -0.111 | 0.005 |
| Time off work, in days | 27.682 | 4.884 | 34.827 | 3.428 | -7.145 | 0.233 | -18.265 | 5.344 |
| **6 months to 12 months** |  |  |  |  |  |  |  |  |
| No. of admissions/visits/consultations: |  |  |  |  |  |  |  |  |
| Inpatient | 0.011 | 0.003 | 0.020 | 0.005 | -0.008 | 0.152 | -0.020 | 0.002 |
| Intensive care unit | 0.003 | 0.002 | 0.002 | 0.001 | 0.001 | 0.646 | -0.003 | 0.005 |
| A&E | 0.044 | 0.009 | 0.045 | 0.006 | -0.001 | 0.896 | -0.022 | 0.022 |
| Outpatient | 0.131 | 0.016 | 0.183 | 0.017 | -0.052 | 0.027 | -0.097 | -0.005 |
| General practitioner | 0.358 | 0.047 | 0.434 | 0.032 | -0.076 | 0.181 | -0.184 | 0.039 |
| No. of participants who: |  |  |  |  |  |  |  |  |
| Received oxygen while in hospital | 0.007 | 0.003 | 0.006 | 0.002 | 0.001 | 0.718 | -0.005 | 0.008 |
| Received mechanical ventilation while in hospital | 0.001 | 0.001 | 0 | 0 | 0.001 | 0.317 | 0.000 | 0.002 |
| Length of stay in hospital, in days | 0.050 | 0.023 | 0.091 | 0.035 | -0.041 | 0.331 | -0.131 | 0.035 |
| Time off work, in days | 36.344 | 5.341 | 46.394 | 5.857 | -10.050 | 0.206 | -25.344 | 5.669 |

*Time off work at 3-month was measured from baseline to 3-month. A&E: accident and emergency, CI: confidence interval, NHS: National Health Service, SE: standard error

## Table S9. Mean (SE) resource use consumption per participant by treatment arm at each time-point (available case): Usual care vs. Ivermectin

|  | **Ivermectin** | | **Usual care** | | **Mean difference** | **p-value** | **Lower 95%CI** | **Upper 95%CI** |
| --- | --- | --- | --- | --- | --- | --- | --- | --- |
|  | **(n=2157)** | | **(n=3256)** | |  |  |  |  |
|  | **Mean** | **SE** | **Mean** | **SE** |  |  |  |  |
| **Baseline to 28 days** |  |  |  |  |  |  |  |  |
| No. of days on: |  |  |  |  |  |  |  |  |
| Anti-diarrheal medication | 0.246 | 0.037 | 0.262 | 0.032 | -0.016 | 0.739 | -0.111 | 0.083 |
| Hay fever tablets (antihistamine) | 3.693 | 0.189 | 3.182 | 0.147 | 0.511 | 0.033 | 0.055 | 0.986 |
| Cold/flu tablets or sachets | 0.954 | 0.063 | 1.206 | 0.062 | -0.253 | 0.004 | -0.426 | -0.076 |
| Cough mixture | 0.978 | 0.068 | 1.539 | 0.079 | -0.561 | <0.001 | -0.769 | -0.360 |
| Co-codamol or codeine-based medication | 1.464 | 0.112 | 1.619 | 0.096 | -0.155 | 0.295 | -0.444 | 0.142 |
| Ibuprofen | 2.548 | 0.116 | 2.594 | 0.101 | -0.046 | 0.766 | -0.345 | 0.250 |
| Inhaler (exclude PRINCIPLE trial inhaler) | 4.752 | 0.223 | 4.554 | 0.179 | 0.198 | 0.488 | -0.358 | 0.768 |
| Paracetamol | 6.172 | 0.172 | 7.780 | 0.159 | -1.608 | <0.001 | -2.065 | -1.144 |
| Steroid nasal spray | 1.823 | 0.134 | 1.659 | 0.106 | 0.164 | 0.339 | -0.166 | 0.499 |
| Throat lozenges | 1.666 | 0.095 | 2.119 | 0.093 | -0.453 | 0.001 | -0.718 | -0.189 |
| No. of admissions/visits/consultations: |  |  |  |  |  |  |  |  |
| Inpatient | 0.072 | 0.006 | 0.111 | 0.006 | -0.039 | <0.001 | -0.056 | -0.021 |
| Intensive care unit | 0.001 | 0.001 | 0.010 | 0.002 | -0.009 | <0.001 | -0.012 | -0.005 |
| NHS111 | 0.007 | 0.002 | 0.053 | 0.006 | -0.045 | <0.001 | -0.058 | -0.032 |
| A&E | 0.005 | 0.002 | 0.032 | 0.004 | -0.028 | <0.001 | -0.037 | -0.019 |
| Outpatient | 0.058 | 0.007 | 0.059 | 0.006 | -0.001 | 0.881 | -0.018 | 0.016 |
| General practitioner | 0.607 | 0.025 | 0.837 | 0.028 | -0.230 | <0.001 | -0.304 | -0.157 |
| Other primary care services | 0.006 | 0.002 | 0.029 | 0.004 | -0.022 | <0.001 | -0.031 | -0.014 |
| No. of participants who: |  |  |  |  |  |  |  |  |
| Took antibiotics | 0.163 | 0.011 | 0.159 | 0.009 | 0.004 | 0.785 | -0.024 | 0.032 |
| Received oxygen while in hospital | 0.006 | 0.002 | 0.030 | 0.003 | -0.024 | <0.001 | -0.032 | -0.017 |
| Received mechanical ventilation while in hospital | 0.001 | 0.001 | 0.006 | 0.001 | -0.005 | 0.003 | -0.008 | -0.002 |
| Length of stay in hospital, in days | 0.073 | 0.019 | 0.453 | 0.061 | -0.380 | <0.001 | -0.506 | -0.262 |
| **29 days to 3 months** |  |  |  |  |  |  |  |  |
| No. of admissions/visits/consultations: |  |  |  |  |  |  |  |  |
| Inpatient | 0.008 | 0.003 | 0.011 | 0.003 | -0.002 | 0.541 | -0.010 | 0.005 |
| Intensive care unit | 0.002 | 0.001 | 0.003 | 0.001 | -0.001 | 0.638 | -0.005 | 0.003 |
| A&E | 0.015 | 0.003 | 0.026 | 0.005 | -0.010 | 0.076 | -0.023 | 0.001 |
| Outpatient | 0.057 | 0.009 | 0.095 | 0.012 | -0.038 | 0.009 | -0.067 | -0.010 |
| General practitioner | 0.307 | 0.027 | 0.377 | 0.026 | -0.071 | 0.058 | -0.142 | 0.005 |
| No. of participants who: |  |  |  |  |  |  |  |  |
| Received oxygen while in hospital | 0.002 | 0.001 | 0.004 | 0.002 | -0.002 | 0.203 | -0.006 | 0.001 |
| Received mechanical ventilation while in hospital | 0.0006 | 0.0006 | 0 | 0 | 0.0006 | 0.317 | 0.000 | 0.002 |
| Length of stay in hospital, in days | 0.020 | 0.011 | 0.061 | 0.022 | -0.040 | 0.102 | -0.093 | 0.003 |
| Time off work, in days* | 20.540 | 1.885 | 22.724 | 1.493 | -2.184 | 0.364 | -6.745 | 2.624 |
| **3 months to 6 months** |  |  |  |  |  |  |  |  |
| No. of admissions/visits/consultations: |  |  |  |  |  |  |  |  |
| Inpatient | 0.007 | 0.002 | 0.014 | 0.003 | -0.007 | 0.057 | -0.014 | 0.0001 |
| Intensive care unit | 0.0006 | 0.0006 | 0.0004 | 0.0004 | 0.0001 | 0.852 | -0.001 | 0.002 |
| A&E | 0.021 | 0.005 | 0.026 | 0.005 | -0.005 | 0.464 | -0.019 | 0.009 |
| Outpatient | 0.131 | 0.018 | 0.115 | 0.012 | 0.016 | 0.460 | -0.026 | 0.059 |
| General practitioner | 0.281 | 0.026 | 0.343 | 0.024 | -0.062 | 0.077 | -0.131 | 0.010 |
| No. of participants who: |  |  |  |  |  |  |  |  |
| Received oxygen while in hospital | 0.003 | 0.001 | 0.005 | 0.002 | -0.002 | 0.289 | -0.007 | 0.002 |
| Received mechanical ventilation while in hospital | 0 | 0 | 0 | 0 | 0 | - | - | - |
| Length of stay in hospital, in days | 0.024 | 0.012 | 0.082 | 0.024 | -0.058 | 0.030 | -0.113 | -0.008 |
| Time off work, in days | 31.730 | 4.588 | 34.827 | 3.428 | -3.097 | 0.589 | -14.036 | 8.376 |
| **6 months to 12 months** |  |  |  |  |  |  |  |  |
| No. of admissions/visits/consultations: |  |  |  |  |  |  |  |  |
| Inpatient | 0.008 | 0.002 | 0.020 | 0.005 | -0.012 | 0.027 | -0.022 | -0.002 |
| Intensive care unit | 0.001 | 0.001 | 0.002 | 0.001 | -0.001 | 0.391 | -0.004 | 0.001 |
| A&E | 0.021 | 0.006 | 0.045 | 0.006 | -0.024 | 0.005 | -0.041 | -0.007 |
| Outpatient | 0.159 | 0.020 | 0.183 | 0.017 | -0.024 | 0.347 | -0.075 | 0.025 |
| General practitioner | 0.335 | 0.033 | 0.434 | 0.032 | -0.099 | 0.030 | -0.189 | -0.011 |
| No. of participants who: |  |  |  |  |  |  |  |  |
| Received oxygen while in hospital | 0.005 | 0.002 | 0.006 | 0.002 | -0.001 | 0.770 | -0.007 | 0.005 |
| Received mechanical ventilation while in hospital | 0 | 0 | 0 | 0 | 0 | - | - | - |
| Length of stay in hospital, in days | 0.029 | 0.014 | 0.091 | 0.035 | -0.062 | 0.104 | -0.145 | 0.002 |
| Time off work, in days | 40.302 | 7.379 | 46.394 | 5.857 | -6.092 | 0.519 | -23.520 | 13.057 |

*Time off work at 3-month was measured from baseline to 3-month. A&E: accident and emergency, CI: confidence interval, NHS: National Health Service, SE: standard error

## Table S10. Mean cost of resource use consumption per participant by treatment arm at each time-point (available case), in 2022/23£: Usual care vs. Azithromycin

|  | **Azithromycin** | | **Usual care** | | **Mean difference** | **p-value** | **Lower 95%CI** | **Upper 95%CI** |
| --- | --- | --- | --- | --- | --- | --- | --- | --- |
|  | **(n=186)** | | **(n=236)** | |  |  |  |  |
|  | **Mean** | **SE** | **Mean** | **SE** |  |  |  |  |
| **Baseline to 28 days** |  |  |  |  |  |  |  |  |
| Medication | 201.137 | 24.923 | 265.144 | 24.074 | -64.007 | 0.066 | -132.328 | 2.855 |
| Anti-diarrheal medication | 1.313 | 0.566 | 0.791 | 0.237 | 0.522 | 0.396 | -0.549 | 1.851 |
| Hay fever tablets (antihistamine) | 6.743 | 2.260 | 8.670 | 2.085 | -1.928 | 0.531 | -7.730 | 4.176 |
| Cold/flu tablets or sachets | 4.205 | 1.129 | 5.198 | 1.158 | -0.992 | 0.540 | -4.095 | 2.135 |
| Cough mixture | 5.261 | 1.472 | 6.242 | 1.339 | -0.980 | 0.623 | -4.911 | 2.910 |
| Co-codamol or codeine-based medication | 6.358 | 1.664 | 6.469 | 1.256 | -0.112 | 0.957 | -4.034 | 4.095 |
| Ibuprofen | 3.201 | 0.763 | 5.029 | 0.830 | -1.828 | 0.106 | -4.031 | 0.342 |
| Inhaler (exclude PRINCIPLE trial inhaler) | 124.657 | 20.259 | 177.851 | 20.635 | -53.194 | 0.067 | -110.620 | 2.285 |
| Paracetamol | 21.692 | 1.933 | 23.388 | 1.888 | -1.696 | 0.531 | -6.918 | 3.523 |
| Steroid nasal spray | 3.616 | 1.030 | 3.710 | 0.913 | -0.094 | 0.945 | -2.759 | 2.623 |
| Throat lozenges | 24.090 | 6.389 | 27.795 | 5.821 | -3.705 | 0.668 | -20.011 | 13.460 |
| Inhaled corticosteroids | 0 | 0 | 0.531 | 0.365 | -0.531 | 0.163 | -1.329 | 0.000 |
| Antibiotics | 1.051 | 0.682 | 2.439 | 0.999 | -1.388 | 0.252 | -3.930 | 0.922 |
| Inpatient | 388.514 | 110.702 | 463.447 | 100.365 | -74.934 | 0.616 | -368.679 | 220.782 |
| Oxygen | 12.473 | 4.234 | 8.847 | 2.899 | 3.626 | 0.480 | -6.207 | 14.059 |
| Mechanical ventilation | 2.462 | 2.462 | 3.881 | 2.739 | -1.419 | 0.700 | -9.182 | 5.446 |
| Intensive care unit | 23.051 | 16.256 | 18.168 | 12.819 | 4.884 | 0.814 | -36.335 | 48.544 |
| NHS111 | 1.620 | 0.400 | 1.332 | 0.399 | 0.288 | 0.611 | -0.801 | 1.395 |
| A&E | 5.043 | 1.487 | 8.233 | 1.595 | -3.190 | 0.144 | -7.546 | 1.187 |
| Outpatient | 18.306 | 12.677 | 6.970 | 2.630 | 11.336 | 0.382 | -6.818 | 41.216 |
| General practitioner | 65.054 | 7.511 | 68.051 | 6.788 | -2.997 | 0.767 | -22.481 | 16.264 |
| Other primary care services | 5.559 | 1.549 | 5.775 | 1.345 | -0.216 | 0.916 | -4.244 | 3.865 |
| Total cost from the NHS perspective | 655.983 | 116.447 | 779.793 | 104.136 | -123.811 | 0.429 | -428.302 | 182.731 |
| **29 days to 3 months** |  |  |  |  |  |  |  |  |
| Inhaled corticosteroids | 0 | 0 | 0 | 0 | 0 | - | - | - |
| Inpatient | 0 | 0 | 0 | 0 | 0 | - | - | - |
| Oxygen | 0 | 0 | 0 | 0 | 0 | - | - | - |
| Mechanical ventilation | 0 | 0 | 0 | 0 | 0 | - | - | - |
| Intensive care unit | 0 | 0 | 0 | 0 | 0 | - | - | - |
| A&E | 0 | 0 | 0 | 0 | 0 | - | - | - |
| Outpatient | 0 | 0 | 0 | 0 | 0 | - | - | - |
| General practitioner | 0 | 0 | 0 | 0 | 0 | - | - | - |
| Time off work | 0 | 0 | 0 | 0 | 0 | - | - | - |
| Total cost from the NHS perspective | 0 | 0 | 0 | 0 | 0 | - | - | - |
| Total cost from the societal perspective | 0 | 0 | 0 | 0 | 0 | - | - | - |
| **3 months to 6 months** |  |  |  |  |  |  |  |  |
| Inhaled corticosteroids | 0 | 0 | 0.266 | 0.266 | -0.266 | 0.331 | -0.797 | 0.000 |
| Inpatient | 0 | 0 | 0 | 0 | 0 | - | - | - |
| Oxygen | 0 | 0 | 0 | 0 | 0 | - | - | - |
| Mechanical ventilation | 0 | 0 | 0 | 0 | 0 | - | - | - |
| Intensive care unit | 0 | 0 | 0 | 0 | 0 | - | - | - |
| A&E | 0 | 0 | 0 | 0 | 0 | - | - | - |
| Outpatient | 0 | 0 | 0 | 0 | 0 | - | - | - |
| General practitioner | 0 | 0 | 0 | 0 | 0 | - | - | - |
| Time off work* | 0 | 0 | 0 | 0 | 0 | - | - | - |
| Total cost from the NHS perspective | 0 | 0 | 0.266 | 0.266 | -0.266 | 0.331 | -0.797 | 0.000 |
| Total cost from the societal perspective | 0 | 0 | 0.266 | 0.266 | -0.266 | 0.331 | -0.797 | 0.000 |
| **6 months to 12 months** |  |  |  |  |  |  |  |  |
| Inhaled corticosteroids | 0 | 0 | 0 | 0 | 0 | - | - | - |
| Inpatient | 122.407 | 112.766 | 51.857 | 51.857 | 70.550 | 0.571 | -135.641 | 347.291 |
| Oxygen | 2.698 | 2.698 | 0 | 0 | 2.698 | 0.320 | 0.000 | 8.093 |
| Mechanical ventilation | 0 | 0 | 0 | 0 | 0 | - | - | - |
| Intensive care unit | 0 | 0 | 0 | 0 | 0 | - | - | - |
| A&E | 7.791 | 4.625 | 7.363 | 3.543 | 0.428 | 0.942 | -10.222 | 12.594 |
| Outpatient | 90.837 | 24.729 | 76.308 | 22.589 | 14.530 | 0.665 | -50.271 | 80.581 |
| General practitioner | 86.337 | 29.905 | 39.286 | 9.348 | 47.051 | 0.136 | -5.399 | 112.812 |
| Time off work | 2219.679 | 630.094 | 1500.407 | 559.791 | 719.272 | 0.395 | -910.234 | 2387.155 |
| Total cost from the NHS perspective | 290.484 | 126.399 | 164.000 | 63.138 | 126.484 | 0.372 | -112.926 | 436.318 |
| Total cost from the societal perspective | 1705.224 | 487.238 | 1045.682 | 349.909 | 659.542 | 0.273 | -462.723 | 1874.706 |

*Time off work at 3-month was measured from baseline to 3-month. A&E: accident and emergency, CI: confidence interval, NHS: National Health Service, SE: standard error

## Table S11. Mean cost of resource use consumption per participant by treatment arm at each time-point (available case), in 2022/23£: Usual care vs. Doxycycline

|  | **Doxycycline** | | **Usual care** | | **Mean difference** | **p-value** | **Lower 95%CI** | **Upper 95%CI** |
| --- | --- | --- | --- | --- | --- | --- | --- | --- |
|  | **(n=435)** | | **(n=336)** | |  |  |  |  |
|  | **Mean** | **SE** | **Mean** | **SE** |  |  |  |  |
| **Baseline to 28 days** |  |  |  |  |  |  |  |  |
| Medication | 252.734 | 17.271 | 270.024 | 20.564 | -17.290 | 0.520 | -70.348 | 34.811 |
| Anti-diarrheal medication | 0.642 | 0.252 | 0.647 | 0.180 | -0.005 | 0.987 | -0.567 | 0.639 |
| Hay fever tablets (antihistamine) | 9.334 | 1.556 | 10.061 | 1.990 | -0.727 | 0.774 | -5.722 | 4.138 |
| Cold/flu tablets or sachets | 5.278 | 0.921 | 5.878 | 1.160 | -0.600 | 0.685 | -3.537 | 2.257 |
| Cough mixture | 4.903 | 0.772 | 7.231 | 1.186 | -2.328 | 0.101 | -5.141 | 0.371 |
| Co-codamol or codeine-based medication | 5.556 | 1.024 | 6.528 | 1.106 | -0.972 | 0.519 | -3.937 | 1.999 |
| Ibuprofen | 3.645 | 0.508 | 4.827 | 0.712 | -1.181 | 0.177 | -2.924 | 0.480 |
| Inhaler (exclude PRINCIPLE trial inhaler) | 172.944 | 14.640 | 172.849 | 17.083 | 0.094 | 0.997 | -44.011 | 42.802 |
| Paracetamol | 18.874 | 1.253 | 25.573 | 1.661 | -6.699 | 0.001 | -10.816 | -2.668 |
| Steroid nasal spray | 3.586 | 0.710 | 4.582 | 0.877 | -0.996 | 0.378 | -3.222 | 1.169 |
| Throat lozenges | 27.971 | 3.966 | 31.848 | 5.009 | -3.877 | 0.544 | -16.386 | 8.437 |
| Inhaled corticosteroids | 0 | 0 | 0.322 | 0.182 | -0.322 | 0.083 | -0.752 | 0.000 |
| Antibiotics | 1.140 | 0.437 | 3.081 | 0.938 | -1.941 | 0.061 | -4.076 | -0.037 |
| Inpatient | 455.589 | 72.289 | 581.435 | 93.397 | -125.846 | 0.287 | -358.417 | 96.722 |
| Oxygen | 13.333 | 2.803 | 13.810 | 2.999 | -0.476 | 0.908 | -8.352 | 7.400 |
| Mechanical ventilation | 3.159 | 1.819 | 6.815 | 3.030 | -3.657 | 0.301 | -10.905 | 2.848 |
| Intensive care unit | 14.785 | 8.516 | 25.521 | 12.703 | -10.736 | 0.483 | -41.186 | 16.809 |
| NHS111 | 1.144 | 0.287 | 1.287 | 0.321 | -0.142 | 0.741 | -0.980 | 0.692 |
| A&E | 6.315 | 0.989 | 8.774 | 1.471 | -2.459 | 0.166 | -5.948 | 0.922 |
| Outpatient | 5.807 | 1.665 | 5.485 | 1.941 | 0.322 | 0.900 | -4.748 | 5.251 |
| General practitioner | 60.563 | 4.489 | 62.857 | 5.285 | -2.294 | 0.741 | -16.233 | 11.072 |
| Other primary care services | 3.025 | 0.703 | 4.616 | 0.988 | -1.591 | 0.190 | -4.045 | 0.749 |
| Total cost from the NHS perspective | 746.167 | 73.638 | 894.024 | 96.588 | -147.857 | 0.224 | -387.880 | 80.680 |
| **29 days to 3 months** |  |  |  |  |  |  |  |  |
| Inhaled corticosteroids | 0.071 | 0.071 | 0 | 0 | 0.071 | 0.321 | 0.000 | 0.213 |
| Inpatient | 0 | 0 | 0 | 0 | 0 | - | - | - |
| Oxygen | 0 | 0 | 0 | 0 | 0 | - | - | - |
| Mechanical ventilation | 0 | 0 | 0 | 0 | 0 | - | - | - |
| Intensive care unit | 0 | 0 | 0 | 0 | 0 | - | - | - |
| A&E | 0 | 0 | 0 | 0 | 0 | - | - | - |
| Outpatient | 0 | 0 | 0 | 0 | 0 | - | - | - |
| General practitioner | 0 | 0 | 0 | 0 | 0 | - | - | - |
| Time off work | 0 | 0 | 0 | 0 | 0 | - | - | - |
| Total cost from the NHS perspective | 0.071 | 0.071 | 0 | 0 | 0.071 | 0.321 | 0.000 | 0.213 |
| Total cost from the societal perspective | 0.071 | 0.071 | 0 | 0 | 0.071 | 0.321 | 0.000 | 0.213 |
| **3 months to 6 months** |  |  |  |  |  |  |  |  |
| Inhaled corticosteroids | 0 | 0 | 0.215 | 0.150 | -0.215 | 0.160 | -0.537 | 0.000 |
| Inpatient | 48.539 | 48.539 | 218.384 | 151.267 | -169.844 | 0.288 | -528.346 | 82.161 |
| Oxygen | 2.275 | 2.275 | 12.712 | 7.694 | -10.438 | 0.197 | -28.603 | 2.275 |
| Mechanical ventilation | 0 | 0 | 0 | 0 | 0 | - | - | - |
| Intensive care unit | 0 | 0 | 0 | 0 | 0 | - | - | - |
| A&E | 4.598 | 1.927 | 0.918 | 0.918 | 3.680 | 0.087 | -0.261 | 7.882 |
| Outpatient | 59.569 | 24.961 | 32.699 | 16.757 | 26.870 | 0.373 | -29.668 | 88.974 |
| General practitioner | 41.520 | 8.786 | 42.945 | 13.708 | -1.426 | 0.930 | -35.662 | 27.870 |
| Time off work* | 1406.455 | 418.451 | 1333.490 | 482.658 | 72.965 | 0.909 | -1178.464 | 1293.199 |
| Total cost from the NHS perspective | 107.014 | 43.524 | 224.386 | 121.208 | -117.373 | 0.364 | -409.483 | 99.312 |
| Total cost from the societal perspective | 728.916 | 215.611 | 807.789 | 290.807 | -78.873 | 0.828 | -814.055 | 614.089 |
| **6 months to 12 months** |  |  |  |  |  |  |  |  |
| Inhaled corticosteroids | 0 | 0 | 0 | 0 | 0 | - | - | - |
| Inpatient | 95.248 | 54.232 | 163.487 | 108.556 | -68.239 | 0.574 | -338.105 | 136.156 |
| Oxygen | 2.100 | 1.481 | 6.187 | 4.881 | -4.087 | 0.424 | -15.908 | 3.702 |
| Mechanical ventilation | 0 | 0 | 0 | 0 | 0 | - | - | - |
| Intensive care unit | 9.700 | 9.700 | 0 | 0 | 9.700 | 0.318 | 0.000 | 29.101 |
| A&E | 4.548 | 1.289 | 4.913 | 2.204 | -0.366 | 0.886 | -5.615 | 4.261 |
| Outpatient | 56.950 | 14.820 | 69.440 | 16.074 | -12.490 | 0.568 | -55.634 | 29.313 |
| General practitioner | 47.036 | 7.555 | 34.100 | 6.471 | 12.936 | 0.194 | -6.381 | 32.294 |
| Time off work | 1618.155 | 371.055 | 1065.692 | 377.729 | 552.464 | 0.298 | -478.837 | 1565.883 |
| Total cost from the NHS perspective | 180.143 | 57.176 | 245.729 | 101.476 | -65.586 | 0.574 | -319.763 | 140.933 |
| Total cost from the societal perspective | 1138.091 | 235.856 | 862.033 | 245.949 | 276.059 | 0.418 | -411.082 | 940.629 |

*Time off work at 3-month was measured from baseline to 3-month. A&E: accident and emergency, CI: confidence interval, NHS: National Health Service, SE: standard error

## Table S12. Mean cost of resource use consumption per participant by treatment arm at each time-point (available case), in 2022/23£: Usual care vs. Budesonide

|  | **Budesonide** | | **Usual care** | | **Mean difference** | **p-value** | **Lower 95%CI** | **Upper 95%CI** |
| --- | --- | --- | --- | --- | --- | --- | --- | --- |
|  | **(n=787)** | | **(n=1069)** | |  |  |  |  |
|  | **Mean** | **SE** | **Mean** | **SE** |  |  |  |  |
| **Baseline to 28 days** |  |  |  |  |  |  |  |  |
| Medication | 216.589 | 10.856 | 193.784 | 10.045 | 22.805 | 0.123 | -6.118 | 51.808 |
| Anti-diarrheal medication | 0.601 | 0.144 | 0.819 | 0.172 | -0.218 | 0.330 | -0.664 | 0.223 |
| Hay fever tablets (antihistamine) | 5.008 | 0.949 | 9.158 | 1.104 | -4.150 | 0.004 | -7.008 | -1.364 |
| Cold/flu tablets or sachets | 3.600 | 0.544 | 6.368 | 0.736 | -2.768 | 0.003 | -4.562 | -0.991 |
| Cough mixture | 5.140 | 0.627 | 7.793 | 0.729 | -2.653 | 0.006 | -4.568 | -0.797 |
| Co-codamol or codeine-based medication | 5.606 | 0.729 | 6.431 | 0.678 | -0.825 | 0.407 | -2.766 | 1.155 |
| Ibuprofen | 4.183 | 0.448 | 4.931 | 0.432 | -0.748 | 0.230 | -1.974 | 0.474 |
| Inhaler (exclude PRINCIPLE trial inhaler) | 145.000 | 8.782 | 93.292 | 7.700 | 51.708 | <0.001 | 28.917 | 74.713 |
| Paracetamol | 19.296 | 0.951 | 24.771 | 0.913 | -5.476 | <0.001 | -8.104 | -2.902 |
| Steroid nasal spray | 2.486 | 0.399 | 3.167 | 0.441 | -0.682 | 0.252 | -1.823 | 0.494 |
| Throat lozenges | 25.669 | 2.888 | 37.053 | 3.196 | -11.384 | 0.008 | -19.878 | -2.982 |
| Inhaled corticosteroids | 0.050 | 0.025 | 0.072 | 0.029 | -0.021 | 0.583 | -0.095 | 0.053 |
| Antibiotics | 1.937 | 0.502 | 2.793 | 0.514 | -0.856 | 0.234 | -2.242 | 0.597 |
| Inpatient | 505.287 | 57.417 | 648.761 | 57.974 | -143.474 | 0.079 | -302.150 | 18.656 |
| Oxygen | 14.740 | 2.262 | 18.664 | 2.117 | -3.925 | 0.205 | -9.940 | 2.156 |
| Mechanical ventilation | 5.238 | 1.922 | 8.569 | 1.993 | -3.331 | 0.229 | -8.779 | 2.118 |
| Intensive care unit | 38.136 | 10.818 | 58.157 | 11.735 | -20.021 | 0.210 | -51.539 | 10.929 |
| NHS111 | 1.548 | 0.250 | 1.422 | 0.202 | 0.127 | 0.694 | -0.489 | 0.759 |
| A&E | 3.065 | 0.662 | 5.077 | 0.760 | -2.012 | 0.046 | -3.969 | -0.045 |
| Outpatient | 5.910 | 1.395 | 6.975 | 1.371 | -1.065 | 0.586 | -4.834 | 2.728 |
| General practitioner | 60.870 | 3.455 | 65.136 | 2.974 | -4.265 | 0.350 | -13.334 | 4.913 |
| Other primary care services | 2.090 | 0.437 | 2.902 | 0.412 | -0.812 | 0.177 | -1.971 | 0.392 |
| Total cost from the NHS perspective | 747.644 | 58.343 | 884.928 | 59.112 | -137.283 | 0.099 | -299.981 | 28.700 |
| **29 days to 3 months** |  |  |  |  |  |  |  |  |
| Inhaled corticosteroids | 0.013 | 0.013 | 0 | 0 | 0.013 | 0.318 | 0.000 | 0.038 |
| Inpatient | 0 | 0 | 150.412 | 83.366 | -150.412 | 0.073 | -333.598 | -6.681 |
| Oxygen | 0 | 0 | 5.693 | 2.820 | -5.693 | 0.045 | -11.387 | -1.423 |
| Mechanical ventilation | 0 | 0 | 0 | 0 | 0 | - | - | - |
| Intensive care unit | 0 | 0 | 52.608 | 32.049 | -52.608 | 0.103 | -118.368 | 0.000 |
| A&E | 3.392 | 3.392 | 1.644 | 1.002 | 1.748 | 0.622 | -3.288 | 9.766 |
| Outpatient | 31.589 | 12.450 | 45.264 | 16.075 | -13.675 | 0.502 | -56.152 | 24.318 |
| General practitioner | 24.715 | 6.139 | 31.043 | 6.351 | -6.328 | 0.474 | -23.761 | 11.276 |
| Time off work | 1248.907 | 268.827 | 1308.588 | 281.796 | -59.680 | 0.878 | -808.257 | 704.640 |
| Total cost from the NHS perspective | 22.101 | 7.634 | 81.630 | 31.020 | -59.529 | 0.063 | -129.990 | -5.912 |
| Total cost from the societal perspective | 267.787 | 61.192 | 357.122 | 82.395 | -89.334 | 0.384 | -294.867 | 106.835 |
| **3 months to 6 months** |  |  |  |  |  |  |  |  |
| Inhaled corticosteroids | 0.013 | 0.013 | 0.036 | 0.021 | -0.023 | 0.338 | -0.072 | 0.025 |
| Inpatient | 42.582 | 19.603 | 106.476 | 39.124 | -63.894 | 0.145 | -153.828 | 16.713 |
| Oxygen | 1.313 | 0.757 | 3.013 | 1.423 | -1.700 | 0.292 | -5.151 | 1.328 |
| Mechanical ventilation | 0 | 0 | 0 | 0 | 0 | - | - | - |
| Intensive care unit | 8.090 | 8.090 | 3.977 | 3.977 | 4.112 | 0.648 | -11.932 | 24.269 |
| A&E | 4.677 | 1.690 | 1.989 | 0.550 | 2.688 | 0.131 | -0.202 | 6.727 |
| Outpatient | 35.621 | 6.061 | 35.831 | 5.872 | -0.210 | 0.980 | -16.465 | 16.113 |
| General practitioner | 30.509 | 3.852 | 30.612 | 3.534 | -0.103 | 0.984 | -10.018 | 10.064 |
| Time off work* | 1226.275 | 206.987 | 1121.142 | 185.363 | 105.134 | 0.705 | -425.521 | 645.914 |
| Total cost from the NHS perspective | 93.616 | 19.672 | 135.475 | 32.918 | -41.859 | 0.275 | -119.261 | 30.749 |
| Total cost from the societal perspective | 641.811 | 99.954 | 621.948 | 92.035 | 19.863 | 0.884 | -245.339 | 283.595 |
| **6 months to 12 months** |  |  |  |  |  |  |  |  |
| Inhaled corticosteroids | 0 | 0 | 0 | 0 | 0 | - | - | - |
| Inpatient | 59.690 | 25.217 | 89.005 | 38.632 | -29.315 | 0.525 | -124.286 | 54.759 |
| Oxygen | 2.795 | 1.975 | 1.930 | 1.279 | 0.865 | 0.713 | -3.316 | 5.990 |
| Mechanical ventilation | 0.920 | 0.920 | 0 | 0 | 0.920 | 0.318 | 0.000 | 2.759 |
| Intensive care unit | 4.305 | 4.305 | 0 | 0 | 4.305 | 0.318 | 0.000 | 12.914 |
| A&E | 4.843 | 1.668 | 4.459 | 1.101 | 0.384 | 0.848 | -3.325 | 4.616 |
| Outpatient | 49.239 | 8.890 | 64.992 | 8.177 | -15.753 | 0.192 | -38.960 | 8.089 |
| General practitioner | 30.703 | 4.652 | 31.115 | 3.320 | -0.412 | 0.943 | -11.312 | 11.052 |
| Time off work | 1152.904 | 221.115 | 1324.096 | 232.808 | -171.192 | 0.594 | -786.593 | 460.140 |
| Total cost from the NHS perspective | 113.843 | 24.882 | 152.724 | 34.610 | -38.881 | 0.362 | -125.403 | 38.666 |
| Total cost from the societal perspective | 668.404 | 120.047 | 816.547 | 127.937 | -148.143 | 0.399 | -494.275 | 187.606 |

*Time off work at 3-month was measured from baseline to 3-month. A&E: accident and emergency, CI: confidence interval, NHS: National Health Service, SE: standard error

## Table S13. Mean cost of resource use consumption per participant by treatment arm at each time-point (available case), in 2022/23£: Usual care vs. Colchicine

|  | **Colchicine** | | **Usual care** | | **Mean difference** | **p-value** | **Lower 95%CI** | **Upper 95%CI** |
| --- | --- | --- | --- | --- | --- | --- | --- | --- |
|  | **(n=156)** | | **(n=1145)** | |  |  |  |  |
|  | **Mean** | **SE** | **Mean** | **SE** |  |  |  |  |
| **Baseline to 28 days** |  |  |  |  |  |  |  |  |
| Medication | 289.821 | 32.287 | 200.457 | 9.889 | 89.365 | 0.009 | 26.169 | 156.217 |
| Anti-diarrheal medication | 0.605 | 0.365 | 0.784 | 0.161 | -0.179 | 0.654 | -0.842 | 0.719 |
| Hay fever tablets (antihistamine) | 16.203 | 3.782 | 9.848 | 1.110 | 6.354 | 0.109 | -0.921 | 14.388 |
| Cold/flu tablets or sachets | 3.867 | 1.661 | 6.422 | 0.699 | -2.555 | 0.158 | -5.536 | 1.453 |
| Cough mixture | 3.431 | 1.058 | 7.957 | 0.709 | -4.526 | <0.001 | -6.924 | -1.944 |
| Co-codamol or codeine-based medication | 7.373 | 1.969 | 6.699 | 0.669 | 0.674 | 0.746 | -3.057 | 4.963 |
| Ibuprofen | 5.532 | 1.352 | 4.945 | 0.412 | 0.587 | 0.678 | -2.054 | 3.489 |
| Inhaler (exclude PRINCIPLE trial inhaler) | 213.158 | 30.141 | 99.003 | 7.729 | 114.155 | <0.001 | 55.323 | 176.678 |
| Paracetamol | 20.421 | 2.403 | 24.963 | 0.885 | -4.542 | 0.078 | -9.437 | 0.613 |
| Steroid nasal spray | 4.111 | 1.328 | 3.214 | 0.431 | 0.897 | 0.522 | -1.553 | 3.811 |
| Throat lozenges | 15.121 | 3.760 | 36.621 | 3.044 | -21.500 | <0.001 | -30.541 | -11.730 |
| Inhaled corticosteroids | 0.039 | 0.039 | 0.062 | 0.025 | -0.023 | 0.628 | -0.103 | 0.085 |
| Antibiotics | 2.225 | 1.370 | 2.747 | 0.483 | -0.522 | 0.720 | -2.892 | 2.646 |
| Inpatient | 278.853 | 96.088 | 610.744 | 54.447 | -331.892 | 0.003 | -534.805 | -100.670 |
| Oxygen | 5.949 | 2.945 | 17.425 | 1.981 | -11.477 | 0.001 | -18.167 | -4.039 |
| Mechanical ventilation | 0 | 0 | 8.000 | 1.862 | -8.000 | <0.001 | -12.000 | -4.400 |
| Intensive care unit | 0 | 0 | 54.296 | 10.965 | -54.296 | <0.001 | -76.764 | -33.701 |
| NHS111 | 0.504 | 0.202 | 1.419 | 0.195 | -0.915 | 0.001 | -1.445 | -0.346 |
| A&E | 1.288 | 0.958 | 4.740 | 0.710 | -3.451 | 0.004 | -5.539 | -0.875 |
| Outpatient | 3.244 | 2.138 | 6.872 | 1.300 | -3.628 | 0.148 | -8.013 | 1.796 |
| General practitioner | 44.071 | 6.155 | 64.511 | 2.993 | -20.440 | 0.003 | -33.614 | -6.474 |
| Other primary care services | 1.205 | 0.597 | 2.997 | 0.407 | -1.791 | 0.014 | -3.092 | -0.299 |
| Total cost from the NHS perspective | 561.407 | 103.931 | 850.662 | 55.546 | -289.255 | 0.015 | -506.513 | -43.445 |
| **29 days to 3 months** |  |  |  |  |  |  |  |  |
| Inhaled corticosteroids | 0 | 0 | 0 | 0 | 0 | - | - | - |
| Inpatient | 0 | 0 | 184.614 | 81.935 | -184.614 | 0.025 | -364.094 | -44.499 |
| Oxygen | 0 | 0 | 6.566 | 3.069 | -6.566 | 0.034 | -13.132 | -1.094 |
| Mechanical ventilation | 0 | 0 | 0 | 0 | 0 | - | - | - |
| Intensive care unit | 0 | 0 | 40.448 | 24.672 | -40.448 | 0.103 | -101.121 | 0.000 |
| A&E | 3.350 | 2.030 | 1.896 | 0.993 | 1.454 | 0.521 | -2.433 | 6.368 |
| Outpatient | 32.550 | 11.645 | 44.014 | 13.230 | -11.464 | 0.516 | -46.271 | 22.161 |
| General practitioner | 27.500 | 8.530 | 28.797 | 5.348 | -1.297 | 0.898 | -19.834 | 19.107 |
| Time off work | 1356.423 | 402.952 | 1301.801 | 229.008 | 54.621 | 0.906 | -792.891 | 1002.675 |
| Total cost from the NHS perspective | 35.972 | 11.990 | 103.728 | 34.120 | -67.757 | 0.061 | -147.116 | -3.818 |
| Total cost from the societal perspective | 632.413 | 191.177 | 440.232 | 83.271 | 192.181 | 0.358 | -187.400 | 623.969 |
| **3 months to 6 months** |  |  |  |  |  |  |  |  |
| Inhaled corticosteroids | 0 | 0 | 0.031 | 0.018 | -0.031 | 0.083 | -0.072 | 0.000 |
| Inpatient | 122.560 | 122.560 | 115.030 | 39.767 | 7.529 | 0.954 | -178.611 | 303.308 |
| Oxygen | 0.000 | 0.000 | 3.569 | 1.530 | -3.569 | 0.020 | -6.742 | -0.793 |
| Mechanical ventilation | 0 | 0 | 0 | 0 | 0 | - | - | - |
| Intensive care unit | 0 | 0 | 3.665 | 3.665 | -3.665 | 0.318 | -10.994 | 0.000 |
| A&E | 3.988 | 2.625 | 2.062 | 0.555 | 1.927 | 0.475 | -2.176 | 8.078 |
| Outpatient | 5.167 | 3.631 | 35.981 | 5.714 | -30.815 | <0.001 | -44.142 | -17.487 |
| General practitioner | 27.500 | 14.625 | 29.803 | 3.342 | -2.303 | 0.878 | -26.070 | 31.027 |
| Time off work* | 1347.818 | 388.190 | 1153.379 | 174.145 | 194.439 | 0.649 | -593.205 | 1063.780 |
| Total cost from the NHS perspective | 94.183 | 77.571 | 139.320 | 33.108 | -45.137 | 0.593 | -175.457 | 145.884 |
| Total cost from the societal perspective | 739.617 | 226.097 | 646.927 | 89.657 | 92.690 | 0.704 | -349.797 | 590.540 |
| **6 months to 12 months** |  |  |  |  |  |  |  |  |
| Inhaled corticosteroids | 0 | 0 | 0 | 0 | 0 | - | - | - |
| Inpatient | 87.725 | 57.121 | 85.515 | 35.820 | 2.210 | 0.974 | -118.788 | 141.267 |
| Oxygen | 1.933 | 1.933 | 2.495 | 1.378 | -0.561 | 0.813 | -4.633 | 4.375 |
| Mechanical ventilation | 0 | 0 | 0 | 0 | 0 | - | - | - |
| Intensive care unit | 0 | 0 | 0 | 0 | 0 | - | - | - |
| A&E | 2.792 | 1.461 | 4.425 | 1.041 | -1.634 | 0.363 | -4.853 | 2.040 |
| Outpatient | 59.675 | 37.034 | 67.333 | 9.107 | -7.658 | 0.841 | -63.301 | 77.294 |
| General practitioner | 33.917 | 10.316 | 31.851 | 3.308 | 2.066 | 0.849 | -16.798 | 24.609 |
| Time off work | 2133.175 | 614.174 | 1524.061 | 230.396 | 609.115 | 0.355 | -578.133 | 1953.519 |
| Total cost from the NHS perspective | 149.277 | 62.255 | 150.146 | 32.369 | -0.869 | 0.990 | -126.516 | 147.098 |
| Total cost from the societal perspective | 1432.065 | 404.147 | 921.470 | 127.605 | 510.595 | 0.230 | -251.759 | 1390.146 |

*Time off work at 3-month was measured from baseline to 3-month. A&E: accident and emergency, CI: confidence interval, NHS: National Health Service, SE: standard error

## Table S14. Mean cost of resource use consumption per participant by treatment arm at each time-point (available case), in 2022/23£: Usual care vs. Favipiravir

|  | **Favipiravir** | | **Usual care** | | **Mean difference** | | **p-value** | | **Lower 95%CI** | | **Upper 95%CI** | |  |
| --- | --- | --- | --- | --- | --- | --- | --- | --- | --- | --- | --- | --- | --- |
|  | **(n=1829)** | | **(n=3256)** | |  |  |  |  |  |  |  |  |  |
|  | **Mean** | **SE** | **Mean** | **SE** | |  | |  | |  | |  | |
| **Baseline to 28 days** |  |  |  |  | |  | |  | |  | |  | |
| Medication | 231.136 | 8.425 | 254.372 | 6.589 | | -23.236 | | 0.030 | | -43.958 | | -2.425 | |
| Anti-diarrheal medication | 0.570 | 0.100 | 0.655 | 0.079 | | -0.085 | | 0.508 | | -0.337 | | 0.171 | |
| Hay fever tablets (antihistamine) | 19.899 | 1.138 | 18.426 | 0.851 | | 1.473 | | 0.300 | | -1.282 | | 4.244 | |
| Cold/flu tablets or sachets | 5.350 | 0.412 | 8.263 | 0.425 | | -2.913 | | <0.001 | | -4.079 | | -1.742 | |
| Cough mixture | 4.059 | 0.345 | 7.526 | 0.388 | | -3.467 | | <0.001 | | -4.504 | | -2.444 | |
| Co-codamol or codeine-based medication | 5.624 | 0.499 | 6.637 | 0.395 | | -1.013 | | 0.111 | | -2.268 | | 0.236 | |
| Ibuprofen | 6.053 | 0.324 | 6.978 | 0.272 | | -0.925 | | 0.029 | | -1.720 | | -0.097 | |
| Inhaler (exclude PRINCIPLE trial inhaler) | 141.064 | 7.208 | 136.938 | 5.381 | | 4.127 | | 0.646 | | -13.454 | | 21.508 | |
| Paracetamol | 16.461 | 0.553 | 24.351 | 0.498 | | -7.890 | | <0.001 | | -9.313 | | -6.391 | |
| Steroid nasal spray | 4.498 | 0.400 | 4.861 | 0.310 | | -0.363 | | 0.472 | | -1.324 | | 0.636 | |
| Throat lozenges | 27.558 | 1.798 | 39.738 | 1.750 | | -12.179 | | <0.001 | | -17.049 | | -7.191 | |
| Inhaled corticosteroids | 0.208 | 0.024 | 0.175 | 0.018 | | 0.034 | | 0.258 | | -0.024 | | 0.092 | |
| Antibiotics | 2.713 | 0.352 | 2.640 | 0.291 | | 0.072 | | 0.874 | | -0.816 | | 0.980 | |
| Inpatient | 116.954 | 16.361 | 259.088 | 20.767 | | -142.134 | | <0.001 | | -193.861 | | -90.383 | |
| Oxygen | 2.410 | 0.632 | 7.054 | 0.774 | | -4.644 | | <0.001 | | -6.584 | | -2.672 | |
| Mechanical ventilation | 0 | 0 | 2.954 | 0.673 | | -2.954 | | <0.001 | | -4.361 | | -1.688 | |
| Intensive care unit | 0 | 0 | 20.411 | 3.990 | | -20.411 | | <0.001 | | -28.311 | | -12.510 | |
| NHS111 | 0.272 | 0.072 | 0.688 | 0.082 | | -0.416 | | <0.001 | | -0.631 | | -0.199 | |
| A&E | 0.696 | 0.197 | 2.161 | 0.280 | | -1.465 | | <0.001 | | -2.153 | | -0.811 | |
| Outpatient | 10.348 | 1.418 | 10.139 | 1.264 | | 0.208 | | 0.913 | | -3.556 | | 3.907 | |
| General practitioner | 35.965 | 1.605 | 46.047 | 1.522 | | -10.082 | | <0.001 | | -14.473 | | -5.846 | |
| Other primary care services | 0.488 | 0.152 | 1.342 | 0.167 | | -0.854 | | <0.001 | | -1.294 | | -0.414 | |
| Total cost from the NHS perspective | 378.763 | 18.750 | 541.428 | 21.864 | | -162.664 | | <0.001 | | -218.828 | | -106.167 | |
| **29 days to 3 months** |  |  |  |  | |  | |  | |  | |  | |
| Inhaled corticosteroids | 0.003 | 0.003 | 0.004 | 0.003 | | -0.001 | | 0.777 | | -0.008 | | 0.006 | |
| Inpatient | 27.538 | 9.588 | 49.550 | 13.071 | | -22.012 | | 0.175 | | -55.154 | | 9.059 | |
| Oxygen | 0.462 | 0.267 | 0.956 | 0.377 | | -0.494 | | 0.285 | | -1.434 | | 0.362 | |
| Mechanical ventilation | 0.304 | 0.304 | 0 | 0 | | 0.304 | | 0.317 | | 0.000 | | 0.912 | |
| Intensive care unit | 1.423 | 1.423 | 5.519 | 2.919 | | -4.096 | | 0.207 | | -11.039 | | 1.743 | |
| A&E | 1.869 | 0.347 | 1.725 | 0.325 | | 0.144 | | 0.763 | | -0.800 | | 1.076 | |
| Outpatient | 14.553 | 2.298 | 20.560 | 2.545 | | -6.007 | | 0.080 | | -12.800 | | 0.821 | |
| General practitioner | 16.909 | 1.704 | 20.760 | 1.418 | | -3.850 | | 0.082 | | -8.115 | | 0.510 | |
| Time off work | 661.193 | 67.401 | 872.433 | 56.433 | | -211.240 | | 0.016 | | -381.569 | | -29.209 | |
| Total cost from the NHS perspective | 50.205 | 8.957 | 68.040 | 10.246 | | -17.835 | | 0.190 | | -44.784 | | 8.791 | |
| Total cost from the societal perspective | 466.981 | 45.628 | 546.013 | 36.326 | | -79.032 | | 0.175 | | -187.637 | | 38.520 | |
| **3 months to 6 months** |  |  |  |  | |  | |  | |  | |  | |
| Inhaled corticosteroids | 0 | 0 | 0.006 | 0.003 | | -0.006 | | 0.083 | | -0.014 | | 0.000 | |
| Inpatient | 20.442 | 9.372 | 52.565 | 12.166 | | -32.123 | | 0.037 | | -62.674 | | -1.729 | |
| Oxygen | 0.463 | 0.345 | 1.216 | 0.429 | | -0.753 | | 0.172 | | -1.824 | | 0.323 | |
| Mechanical ventilation | 0 | 0 | 0 | 0 | | 0 | | - | | - | | - | |
| Intensive care unit | 0 | 0 | 0.937 | 0.937 | | -0.937 | | 0.317 | | -2.810 | | 0.000 | |
| A&E | 1.783 | 0.506 | 1.756 | 0.327 | | 0.027 | | 0.964 | | -1.111 | | 1.239 | |
| Outpatient | 22.379 | 3.226 | 25.028 | 2.522 | | -2.649 | | 0.518 | | -10.641 | | 5.515 | |
| General practitioner | 14.930 | 1.555 | 18.886 | 1.304 | | -3.956 | | 0.051 | | -7.862 | | 0.242 | |
| Time off work* | 453.495 | 56.610 | 644.188 | 54.011 | | -190.693 | | 0.015 | | -340.143 | | -38.590 | |
| Total cost from the NHS perspective | 48.976 | 9.589 | 78.163 | 10.948 | | -29.187 | | 0.045 | | -57.005 | | -0.450 | |
| Total cost from the societal perspective | 341.377 | 39.019 | 449.405 | 35.661 | | -108.027 | | 0.041 | | -210.629 | | -4.768 | |
| **6 months to 12 months** |  |  |  |  | |  | |  | |  | |  | |
| Inhaled corticosteroids | 0.017 | 0.007 | 0.006 | 0.003 | | 0.011 | | 0.153 | | -0.003 | | 0.027 | |
| Inpatient | 41.486 | 14.848 | 65.876 | 16.043 | | -24.390 | | 0.265 | | -67.302 | | 17.957 | |
| Oxygen | 1.613 | 0.638 | 1.332 | 0.445 | | 0.281 | | 0.718 | | -1.177 | | 1.871 | |
| Mechanical ventilation | 0.290 | 0.290 | 0.000 | 0.000 | | 0.290 | | 0.317 | | 0.000 | | 0.869 | |
| Intensive care unit | 5.420 | 3.318 | 3.516 | 2.486 | | 1.905 | | 0.646 | | -6.079 | | 10.841 | |
| A&E | 2.922 | 0.625 | 3.022 | 0.430 | | -0.099 | | 0.896 | | -1.506 | | 1.503 | |
| Outpatient | 28.531 | 3.569 | 39.770 | 3.625 | | -11.239 | | 0.027 | | -21.112 | | -1.114 | |
| General practitioner | 19.678 | 2.596 | 23.881 | 1.765 | | -4.203 | | 0.181 | | -10.094 | | 2.147 | |
| Time off work | 799.897 | 89.353 | 1063.199 | 89.004 | | -263.302 | | 0.037 | | -510.911 | | -14.353 | |
| Total cost from the NHS perspective | 80.214 | 15.908 | 110.304 | 15.318 | | -30.090 | | 0.173 | | -72.580 | | 13.896 | |
| Total cost from the societal perspective | 630.553 | 66.985 | 778.476 | 61.115 | | -147.923 | | 0.103 | | -324.542 | | 32.775 | |

*Time off work at 3-month was measured from baseline to 3-month. A&E: accident and emergency, CI: confidence interval, NHS: National Health Service, SE: standard error

## Table S15. Mean cost of resource use consumption per participant by treatment arm at each time-point (available case), in 2022/23£: Usual care vs. Ivermectin

|  | **Ivermectin** | | **Usual care** | | **Mean difference** | **p-value** | **Lower 95%CI** | **Upper 95%CI** |
| --- | --- | --- | --- | --- | --- | --- | --- | --- |
|  | **(n=2157)** | | **(n=3256)** | |  |  |  |  |
|  | **Mean** | **SE** | **Mean** | **SE** |  |  |  |  |
| **Baseline to 28 days** |  |  |  |  |  |  |  |  |
| Medication | 244.977 | 7.836 | 254.372 | 6.589 | -9.395 | 0.359 | -29.289 | 10.991 |
| Anti-diarrheal medication | 0.614 | 0.093 | 0.655 | 0.079 | -0.041 | 0.739 | -0.277 | 0.207 |
| Hay fever tablets (antihistamine) | 21.382 | 1.093 | 18.426 | 0.851 | 2.956 | 0.033 | 0.317 | 5.711 |
| Cold/flu tablets or sachets | 6.533 | 0.434 | 8.263 | 0.425 | -1.730 | 0.004 | -2.921 | -0.518 |
| Cough mixture | 4.781 | 0.331 | 7.526 | 0.388 | -2.745 | <0.001 | -3.762 | -1.763 |
| Co-codamol or codeine-based medication | 6.003 | 0.459 | 6.637 | 0.395 | -0.634 | 0.295 | -1.820 | 0.582 |
| Ibuprofen | 6.854 | 0.312 | 6.978 | 0.272 | -0.123 | 0.766 | -0.927 | 0.671 |
| Inhaler (exclude PRINCIPLE trial inhaler) | 142.905 | 6.703 | 136.938 | 5.381 | 5.967 | 0.488 | -10.775 | 23.093 |
| Paracetamol | 19.318 | 0.539 | 24.351 | 0.498 | -5.033 | <0.001 | -6.464 | -3.581 |
| Steroid nasal spray | 5.340 | 0.394 | 4.861 | 0.310 | 0.479 | 0.339 | -0.486 | 1.462 |
| Throat lozenges | 31.247 | 1.790 | 39.738 | 1.750 | -8.491 | 0.001 | -13.460 | -3.552 |
| Inhaled corticosteroids | 0.218 | 0.022 | 0.175 | 0.018 | 0.043 | 0.132 | -0.011 | 0.100 |
| Antibiotics | 2.808 | 0.361 | 2.618 | 0.290 | 0.190 | 0.681 | -0.698 | 1.113 |
| Inpatient | 86.074 | 11.709 | 259.088 | 20.767 | -173.014 | <0.001 | -218.830 | -127.565 |
| Oxygen | 1.398 | 0.387 | 7.054 | 0.774 | -5.656 | <0.001 | -7.367 | -3.975 |
| Mechanical ventilation | 0.637 | 0.368 | 2.954 | 0.673 | -2.317 | 0.003 | -3.798 | -0.833 |
| Intensive care unit | 1.988 | 1.405 | 20.411 | 3.990 | -18.423 | <0.001 | -26.659 | -10.187 |
| NHS111 | 0.097 | 0.030 | 0.688 | 0.082 | -0.591 | <0.001 | -0.766 | -0.425 |
| A&E | 0.311 | 0.116 | 2.161 | 0.280 | -1.850 | <0.001 | -2.467 | -1.263 |
| Outpatient | 8.776 | 1.218 | 10.139 | 1.264 | -1.363 | 0.437 | -4.896 | 2.058 |
| General practitioner | 33.377 | 1.394 | 46.047 | 1.522 | -12.670 | <0.001 | -16.719 | -8.643 |
| Other primary care services | 0.305 | 0.111 | 1.342 | 0.167 | -1.037 | <0.001 | -1.435 | -0.639 |
| Total cost from the NHS perspective | 355.314 | 14.320 | 541.406 | 21.863 | -186.092 | <0.001 | -236.691 | -135.460 |
| **29 days to 3 months** |  |  |  |  |  |  |  |  |
| Inhaled corticosteroids | 0.007 | 0.004 | 0.004 | 0.003 | 0.003 | 0.521 | -0.006 | 0.013 |
| Inpatient | 19.848 | 7.954 | 49.550 | 13.071 | -29.702 | 0.052 | -59.981 | -1.291 |
| Oxygen | 0.394 | 0.228 | 0.956 | 0.377 | -0.561 | 0.203 | -1.434 | 0.263 |
| Mechanical ventilation | 0.259 | 0.259 | 0 | 0 | 0.259 | 0.317 | 0.000 | 0.778 |
| Intensive care unit | 3.644 | 2.715 | 5.519 | 2.919 | -1.876 | 0.638 | -9.714 | 6.073 |
| A&E | 1.025 | 0.223 | 1.725 | 0.325 | -0.700 | 0.076 | -1.514 | 0.073 |
| Outpatient | 12.295 | 1.883 | 20.560 | 2.545 | -8.266 | 0.009 | -14.647 | -2.097 |
| General practitioner | 16.858 | 1.486 | 20.760 | 1.418 | -3.901 | 0.058 | -7.784 | 0.274 |
| Time off work | 812.373 | 58.719 | 872.433 | 56.433 | -60.060 | 0.461 | -221.026 | 101.177 |
| Total cost from the NHS perspective | 40.941 | 7.326 | 68.040 | 10.246 | -27.098 | 0.032 | -52.553 | -2.673 |
| Total cost from the societal perspective | 550.887 | 39.561 | 546.013 | 36.326 | 4.874 | 0.928 | -100.004 | 110.263 |
| **3 months to 6 months** |  |  |  |  |  |  |  |  |
| Inhaled corticosteroids | 0 | 0 | 0.006 | 0.003 | -0.006 | 0.083 | -0.014 | 0.000 |
| Inpatient | 23.212 | 8.866 | 52.565 | 12.166 | -29.352 | 0.051 | -59.283 | -0.467 |
| Oxygen | 0.663 | 0.296 | 1.216 | 0.429 | -0.553 | 0.289 | -1.598 | 0.445 |
| Mechanical ventilation | 0 | 0 | 0 | 0 | 0 | - | - | - |
| Intensive care unit | 1.225 | 1.225 | 0.937 | 0.937 | 0.288 | 0.852 | -2.810 | 3.675 |
| A&E | 1.417 | 0.330 | 1.756 | 0.327 | -0.340 | 0.464 | -1.243 | 0.572 |
| Outpatient | 28.520 | 3.992 | 25.028 | 2.522 | 3.492 | 0.460 | -5.616 | 12.793 |
| General practitioner | 15.463 | 1.434 | 18.886 | 1.304 | -3.423 | 0.077 | -7.185 | 0.524 |
| Time off work* | 542.507 | 59.845 | 644.188 | 54.011 | -101.681 | 0.207 | -260.495 | 57.330 |
| Total cost from the NHS perspective | 55.666 | 9.009 | 78.163 | 10.948 | -22.497 | 0.113 | -50.369 | 4.896 |
| Total cost from the societal perspective | 401.744 | 41.328 | 449.405 | 35.661 | -47.661 | 0.383 | -152.147 | 62.639 |
| **6 months to 12 months** |  |  |  |  |  |  |  |  |
| Inhaled corticosteroids | 0.005 | 0.003 | 0.006 | 0.003 | -0.001 | 0.809 | -0.010 | 0.008 |
| Inpatient | 24.843 | 8.548 | 65.876 | 16.043 | -41.034 | 0.024 | -78.335 | -6.709 |
| Oxygen | 1.132 | 0.518 | 1.332 | 0.445 | -0.200 | 0.770 | -1.532 | 1.153 |
| Mechanical ventilation | 0 | 0 | 0 | 0 | 0 | - | - | - |
| Intensive care unit | 1.162 | 1.162 | 3.516 | 2.486 | -2.354 | 0.391 | -8.790 | 2.324 |
| A&E | 1.416 | 0.381 | 3.022 | 0.430 | -1.605 | 0.005 | -2.727 | -0.474 |
| Outpatient | 34.461 | 4.327 | 39.770 | 3.625 | -5.309 | 0.347 | -16.369 | 5.515 |
| General practitioner | 18.423 | 1.798 | 23.881 | 1.765 | -5.458 | 0.030 | -10.414 | -0.609 |
| Time off work | 746.799 | 86.391 | 1063.199 | 89.004 | -316.400 | 0.011 | -562.644 | -62.989 |
| Total cost from the NHS perspective | 67.700 | 9.645 | 110.304 | 15.318 | -42.604 | 0.019 | -79.321 | -8.766 |
| Total cost from the societal perspective | 585.647 | 62.603 | 778.476 | 61.115 | -192.829 | 0.028 | -360.220 | -18.343 |

*Time off work at 3-month was measured from baseline to 3-month. A&E: accident and emergency, CI: confidence interval, NHS: National Health Service, SE: standard error

## Table S16. Mean outcome by treatment arm at each time-point (available case): Usual care vs. Azithromycin

|  | **Azithromycin** | | **Usual care** | | **Mean difference** | **p-value** | **Lower 95%CI** | **Upper 95%CI** |
| --- | --- | --- | --- | --- | --- | --- | --- | --- |
|  | **(n=186)** | | **(n=236)** | |  |  |  |  |
|  | **Mean** | **SE** | **Mean** | **SE** |  |  |  |  |
| **Baseline to 28 days** |  |  |  |  |  |  |  |  |
| No. of hospitalisation or death | 1.065 | 0.018 | 1.068 | 0.016 | -0.003 | 0.893 | -0.051 | 0.045 |
| Time-to-recovery, days | 7.566 | 0.512 | 9.620 | 0.530 | -2.053 | 0.006 | -3.486 | -0.616 |
| **Baseline to 12 months** |  |  |  |  |  |  |  |  |
| No. of hospitalisation or death | 1.065 | 0.018 | 1.072 | 0.017 | -0.008 | 0.761 | -0.056 | 0.041 |

CI: confidence interval, SE: standard error

## Table S17. Mean outcome by treatment arm at each time-point (available case): Usual care vs. Doxycycline

|  | **Doxycycline** | | **Usual care** | | **Mean difference** | **p-value** | **Lower 95%CI** | **Upper 95%CI** |
| --- | --- | --- | --- | --- | --- | --- | --- | --- |
|  | **(n=435)** | | **(n=336)** | |  |  |  |  |
|  | **Mean** | **SE** | **Mean** | **SE** |  |  |  |  |
| **Baseline to 28 days** |  |  |  |  |  |  |  |  |
| No. of hospitalisation or death | 1.083 | 0.013 | 1.089 | 0.016 | -0.007 | 0.750 | -0.046 | 0.032 |
| Time-to-recovery, days | 9.138 | 0.406 | 9.628 | 0.474 | -0.490 | 0.433 | -1.714 | 0.730 |
| **Baseline to 12 months** |  |  |  |  |  |  |  |  |
| No. of hospitalisation or death | 1.090 | 0.014 | 1.095 | 0.016 | -0.006 | 0.791 | -0.046 | 0.035 |

CI: confidence interval, SE: standard error

## Table S18. Mean outcome by treatment arm at each time-point (available case): Usual care vs. Budesonide

|  | **Budesonide** | | **Usual care** | | **Mean difference** | **p-value** | **Lower 95%CI** | **Upper 95%CI** |
| --- | --- | --- | --- | --- | --- | --- | --- | --- |
|  | **(n=787)** | | **(n=1069)** | |  |  |  |  |
|  | **Mean** | **SE** | **Mean** | **SE** |  |  |  |  |
| **Baseline to 28 days** |  |  |  |  |  |  |  |  |
| No. of hospitalisation or death | 1.089 | 0.010 | 1.107 | 0.009 | -0.018 | 0.202 | -0.044 | 0.010 |
| Time-to-recovery, days | 9.136 | 0.283 | 10.010 | 0.282 | -0.874 | 0.029 | -1.664 | -0.095 |
| **Baseline to 12 months** |  |  |  |  |  |  |  |  |
| No. of hospitalisation or death | 1.097 | 0.011 | 1.115 | 0.010 | -0.018 | 0.198 | -0.046 | 0.010 |

CI: confidence interval, SE: standard error

## Table S19. Mean outcome by treatment arm at each time-point (available case): Usual care vs. Colchicine

|  | **Colchicine** | | **Usual care** | | **Mean difference** | **p-value** | **Lower 95%CI** | **Upper 95%CI** |
| --- | --- | --- | --- | --- | --- | --- | --- | --- |
|  | **(n=156)** | | **(n=1145)** | |  |  |  |  |
|  | **Mean** | **SE** | **Mean** | **SE** |  |  |  |  |
| **Baseline to 28 days** |  |  |  |  |  |  |  |  |
| No. of hospitalisation or death | 1.038 | 0.015 | 1.103 | 0.009 | -0.065 | <0.001 | -0.098 | -0.028 |
| Time-to-recovery, days | 12.085 | 0.773 | 9.901 | 0.270 | 2.184 | 0.009 | 0.571 | 3.784 |
| **Baseline to 12 months** |  |  |  |  |  |  |  |  |
| No. of hospitalisation or death | 1.051 | 0.018 | 1.112 | 0.009 | -0.061 | 0.003 | -0.098 | -0.019 |

CI: confidence interval, SE: standard error

## Table S20. Mean outcome by treatment arm at each time-point (available case): Usual care vs. Favipiravir

|  | **Favipiravir** | | **Usual care** | | **Mean difference** | **p-value** | **Lower 95%CI** | **Upper 95%CI** |
| --- | --- | --- | --- | --- | --- | --- | --- | --- |
|  | **(n=1829)** | | **(n=3256)** | |  |  |  |  |
|  | **Mean** | **SE** | **Mean** | **SE** |  |  |  |  |
| **Baseline to 28 days** |  |  |  |  |  |  |  |  |
| No. of hospitalisation or death | 1.016 | 0.003 | 1.045 | 0.004 | -0.029 | <0.001 | -0.038 | -0.020 |
| Time-to-recovery, days | 10.698 | 0.182 | 11.162 | 0.158 | -0.465 | 0.054 | -0.940 | 0.003 |
| **Baseline to 12 months** |  |  |  |  |  |  |  |  |
| No. of hospitalisation or death | 1.023 | 0.004 | 1.055 | 0.004 | -0.032 | <0.001 | -0.042 | -0.021 |

CI: confidence interval, SE: standard error

## Table S21. Mean outcome by treatment arm at each time-point (available case): Usual care vs. Ivermectin

|  | **Ivermectin** | | **Usual care** | | **Mean difference** | **p-value** | **Lower 95%CI** | **Upper 95%CI** |
| --- | --- | --- | --- | --- | --- | --- | --- | --- |
|  | **(n=2157)** | | **(n=3256)** | |  |  |  |  |
|  | **Mean** | **SE** | **Mean** | **SE** |  |  |  |  |
| **Baseline to 28 days** |  |  |  |  |  |  |  |  |
| No. of hospitalisation or death | 1.015 | 0.003 | 1.045 | 0.004 | -0.030 | <0.001 | -0.038 | -0.021 |
| Time-to-recovery, days | 10.833 | 0.178 | 11.162 | 0.158 | -0.330 | 0.166 | -0.799 | 0.129 |
| **Baseline to 12 months** |  |  |  |  |  |  |  |  |
| No. of hospitalisation or death | 1.024 | 0.003 | 1.055 | 0.004 | -0.031 | <0.001 | -0.041 | -0.021 |

CI: confidence interval, SE: standard error

## Table S22. Sensitivity and subgroup analyses of the cost-effectiveness analysis in terms of incremental cost per hospitalisation or death avoided: Usual care vs. Azithromycin

|  | **Number** | | **Cost** | | | | | | | **Hospitalisation or death** | | | | | | | **ICER** | **ICER quadrant** |
| --- | --- | --- | --- | --- | --- | --- | --- | --- | --- | --- | --- | --- | --- | --- | --- | --- | --- | --- |
|  | **Azithromycin** | **Usual care** | **Azithromycin** | | **Usual care** | | **Mean difference** | **Lower 95%CI** | **Upper 95%CI** | **Azithromycin** | | **Usual care** | | **Mean difference** | **Lower 95%CI** | **Upper 95%CI** |  |  |
|  |  |  | **Mean** | **SE** | **Mean** | **SE** |  |  |  | **Mean** | **SE** | **Mean** | **SE** |  |  |  |  |  |
| [1] | 186 | 236 | 662.64 | 193.57 | 781.04 | 106.13 | -118.40 | -435.54 | 198.73 | 1.064 | 0.030 | 1.068 | 0.016 | -0.004 | -0.053 | 0.045 | 27,490 | SW |
| [2] | 186 | 236 | **-** | **-** | **-** | **-** | **-** | **-** | **-** | **-** | **-** | **-** | **-** | **-** | **-** | **-** | - | - |
| [3] | 186 | 236 | **-** | **-** | **-** | **-** | **-** | **-** | **-** | **-** | **-** | **-** | **-** | **-** | **-** | **-** | - | - |
| [4] | 186 | 225 | 662.68 | 184.08 | 693.53 | 97.84 | -30.85 | -336.31 | 274.60 | 1.063 | 0.029 | 1.063 | 0.016 | 0.001 | -0.046 | 0.048 | -44,433 | SE |
| [5] | 108 | 151 | 712.77 | 198.91 | 703.68 | 102.60 | 9.09 | -324.73 | 342.91 | 1.064 | 0.035 | 1.053 | 0.019 | 0.011 | -0.048 | 0.069 | 853 | NE |
| [6] | 78 | 85 | 599.51 | 373.57 | 906.36 | 224.90 | -306.85 | -891.18 | 277.48 | 1.067 | 0.052 | 1.092 | 0.032 | -0.025 | -0.106 | 0.055 | 12,138 | SW |
| [7] | 29 | 29 | **-** | **-** | **-** | **-** | **-** | **-** | **-** | **-** | **-** | **-** | **-** | **-** | **-** | **-** | - | - |
| [8] | 157 | 207 | 710.28 | 196.59 | 795.68 | 107.35 | -85.40 | -408.03 | 237.24 | 1.066 | 0.032 | 1.072 | 0.018 | -0.006 | -0.058 | 0.045 | 13,494 | SW |
| [9] | 75 | 96 | 836.90 | 342.82 | 991.78 | 190.30 | -154.88 | -713.49 | 403.73 | 1.103 | 0.056 | 1.103 | 0.030 | -0.001 | -0.094 | 0.093 | 225,696 | SW |
| [10] | 111 | 140 | 546.04 | 207.37 | 638.01 | 115.81 | -91.97 | -428.95 | 245.02 | 1.036 | 0.030 | 1.044 | 0.017 | -0.008 | -0.055 | 0.039 | 11,723 | SW |
| [11] | 143 | 198 | 605.66 | 199.00 | 722.73 | 106.29 | -117.06 | -446.64 | 212.51 | 1.060 | 0.030 | 1.060 | 0.017 | -0.0001 | -0.049 | 0.049 | 1,218,428 | SW |
| [12] | 43 | 38 | 838.59 | 489.29 | 1078.81 | 296.45 | -240.22 | -999.36 | 518.92 | 1.073 | 0.079 | 1.108 | 0.045 | -0.035 | -0.162 | 0.093 | 6,941 | SW |
| [13] | 0 | 0 | **-** | **-** | **-** | **-** | **-** | **-** | **-** | **-** | **-** | **-** | **-** | **-** | **-** | **-** | **-** | - |
| [14] | 0 | 0 | **-** | **-** | **-** | **-** | **-** | **-** | **-** | **-** | **-** | **-** | **-** | **-** | **-** | **-** | **-** | - |

ICER: incremental cost-effectiveness ratio, CI: confidence interval, NE: north-east, NW: north-west, SE (under ICER quadrant): south-east, SE: standard error, SW: south-west

[1] Sensitivity analysis: Complete case analysis – same as the base case results because there were no missing data in the base case analysis. Adjusted for age category, comorbidity and duration of illness.

[2] Sensitivity analysis: 12-month time horizon – high missingness of resource use data at 3- and 6-months.

[3] Sensitivity analysis: Societal perspective – high missingness of resource use data at 3- and 6-months.

[4] Sensitivity analysis: Concurrent randomisation analysis population. Adjusted for age category, comorbidity and duration of illness.

[5] Subgroup analysis: Aged 50-64 years. Adjusted for duration of illness.

[6] Subgroup analysis: Aged ≥65 years. Adjusted for comorbidity and duration of illness.

[7] Subgroup analysis: No comorbidity at baseline – numbers were too small for computation.

[8] Subgroup analysis: Had comorbidity at baseline. Adjusted for age category and duration of illness.

[9] Subgroup analysis: Duration of symptoms of <7 days before randomisation. Adjusted for age category and comorbidity.

[10] Subgroup analysis: Duration of symptoms of ≥7 days before randomisation. Adjusted for age category and comorbidity.

[11] Subgroup analysis: No symptoms rated as 'major' at baseline. Adjusted for age category, comorbidity and duration of illness.

[12] Subgroup analysis: At least one symptom rated as 'major' at baseline. Adjusted for duration of illness.

[13] Subgroup analysis: Did not use inhaled corticosteroid steroid at randomisation/within 28 days follow-up.

[14] Subgroup analysis: Used inhaled corticosteroid steroid at randomisation/within 28 days follow-up.

## Table S23. Sensitivity and subgroup analyses of the cost-effectiveness analysis in terms of incremental cost per hospitalisation or death avoided: Usual care vs. Doxycycline

|  | **Number** | | **Cost** | | | | | | | **Hospitalisation or death** | | | | | | | **ICER** | **ICER quadrant** |
| --- | --- | --- | --- | --- | --- | --- | --- | --- | --- | --- | --- | --- | --- | --- | --- | --- | --- | --- |
|  | **Doxycycline** | **Usual care** | **Doxycycline** | | **Usual care** | | **Mean difference** | **Lower 95%CI** | **Upper 95%CI** | **Doxycycline** | | **Usual care** | | **Mean difference** | **Lower 95%CI** | **Upper 95%CI** |  |  |
|  |  |  | **Mean** | **SE** | **Mean** | **SE** |  |  |  | **Mean** | **SE** | **Mean** | **SE** |  |  |  |  |  |
| [1] | 435 | 336 | 746.62 | 154.47 | 897.99 | 98.05 | -151.37 | -385.19 | 82.46 | 1.082 | 0.025 | 1.090 | 0.015 | -0.008 | -0.047 | 0.031 | 19,592 | SW |
| [2] | 435 | 336 | **-** | **-** | **-** | **-** | **-** | **-** | **-** | **-** | **-** | **-** | **-** | **-** | **-** | **-** | **-** | **-** |
| [3] | 435 | 336 | **-** | **-** | **-** | **-** | **-** | **-** | **-** | **-** | **-** | **-** | **-** | **-** | **-** | **-** | **-** | **-** |
| [4] | 435 | 321 | 745.35 | 11.64 | 828.36 | 11.64 | -83.01 | -315.49 | 149.46 | 1.082 | 0.002 | 1.084 | 0.002 | -0.002 | -0.041 | 0.038 | 46,084 | SW |
| [5] | 248 | 189 | 705.09 | 8.25 | 678.27 | 8.25 | 26.82 | -212.78 | 266.42 | 1.064 | 0.002 | 1.053 | 0.002 | 0.011 | -0.033 | 0.055 | 2,411 | NE |
| [6] | 187 | 147 | 797.87 | 22.18 | 1171.43 | 22.18 | -373.56 | -814.49 | 67.38 | 1.106 | 0.004 | 1.136 | 0.004 | -0.030 | -0.101 | 0.040 | 12,300 | SW |
| [7] | 63 | 51 | 509.17 | 25.42 | 749.37 | 25.42 | -240.20 | -824.67 | 344.27 | 1.066 | 0.002 | 1.059 | 0.002 | 0.007 | -0.079 | 0.093 | -33,977 | SE |
| [8] | 372 | 285 | 790.78 | 13.03 | 919.91 | 13.03 | -129.13 | -385.45 | 127.20 | 1.085 | 0.003 | 1.095 | 0.003 | -0.010 | -0.054 | 0.034 | 13,270 | SW |
| [9] | 194 | 145 | 841.30 | 10.74 | 858.36 | 10.74 | -17.06 | -370.78 | 336.65 | 1.104 | 0.003 | 1.090 | 0.003 | 0.015 | -0.050 | 0.079 | -1,169 | SE |
| [10] | 241 | 191 | 694.72 | 22.85 | 921.10 | 22.85 | -226.37 | -538.80 | 86.05 | 1.065 | 0.003 | 1.089 | 0.003 | -0.024 | -0.073 | 0.026 | 9,575 | SW |
| [11] | 346 | 272 | 681.92 | 9.05 | 767.49 | 9.05 | -85.57 | -327.92 | 156.78 | 1.076 | 0.002 | 1.074 | 0.002 | 0.002 | -0.039 | 0.044 | -35,020 | SE |
| [12] | 89 | 64 | 1019.66 | 63.58 | 1431.79 | 63.58 | -412.13 | -1084.10 | 259.84 | 1.110 | 0.011 | 1.156 | 0.011 | -0.046 | -0.154 | 0.062 | 9,006 | SW |
| [13] | 343 | 205 | 653.10 | 169.21 | 873.50 | 108.02 | -220.40 | -585.88 | 145.08 | 1.069 | 0.027 | 1.088 | 0.017 | -0.019 | -0.079 | 0.041 | 11,571 | SW |
| [14] | 92 | 131 | 1071.47 | 492.71 | 933.94 | 276.57 | 137.53 | -826.94 | 1102.01 | 1.125 | 0.070 | 1.090 | 0.041 | 0.034 | -0.107 | 0.176 | 3,993 | NE |

ICER: incremental cost-effectiveness ratio, CI: confidence interval, NE: north-east, NW: north-west, SE (under ICER quadrant): south-east, SE: standard error, SW: south-west

[1] Sensitivity analysis: Complete case analysis. Adjusted for age category, comorbidity and duration of illness.

[2] Sensitivity analysis: 12-month time horizon – high missingness of resource use data at 3- and 6-months.

[3] Sensitivity analysis: Societal perspective – high missingness of resource use data at 3- and 6-months.

[4] Sensitivity analysis: Concurrent randomisation analysis population. Adjusted for age category, comorbidity and duration of illness.

[5] Subgroup analysis: Aged 50-64 years. Adjusted for duration of illness.

[6] Subgroup analysis: Aged ≥65 years. Adjusted for comorbidity and duration of illness.

[7] Subgroup analysis: No comorbidity at baseline. Adjusted for duration of illness.

[8] Subgroup analysis: Had comorbidity at baseline. Adjusted for age category and duration of illness.

[9] Subgroup analysis: Duration of symptoms of <7 days before randomisation. Adjusted for age category and comorbidity.

[10] Subgroup analysis: Duration of symptoms of ≥7 days before randomisation. Adjusted for age category and comorbidity.

[11] Subgroup analysis: No symptoms rated as 'major' at baseline. Adjusted for age category, comorbidity and duration of illness.

[12] Subgroup analysis: At least one symptom rated as 'major' at baseline. Adjusted for age category, comorbidity and duration of illness.

[13] Subgroup analysis: Did not use inhaled corticosteroid steroid at randomisation/within 28 days follow-up. Adjusted for age category, comorbidity and duration of illness.

[14] Subgroup analysis: Used inhaled corticosteroid steroid at randomisation/within 28 days follow-up. Adjusted for age category, comorbidity and duration of illness.

## Table S24. Sensitivity and subgroup analyses of the cost-effectiveness analysis in terms of incremental cost per hospitalisation or death avoided: Usual care vs. Budesonide

|  | **Number** | | **Cost** | | | | | | | **Hospitalisation or death** | | | | | | | **ICER** | **ICER quadrant** |
| --- | --- | --- | --- | --- | --- | --- | --- | --- | --- | --- | --- | --- | --- | --- | --- | --- | --- | --- |
|  | **Budesonide** | **Usual care** | **Budesonide** | | **Usual care** | | **Mean difference** | **Lower 95%CI** | **Upper 95%CI** | **Budesonide** | | **Usual care** | | **Mean difference** | **Lower 95%CI** | **Upper 95%CI** |  |  |
|  |  |  | **Mean** | **SE** | **Mean** | **SE** |  |  |  | **Mean** | **SE** | **Mean** | **SE** |  |  |  |  |  |
| [1] | 476 | 516 | 878.47 | 150.65 | 968.13 | 93.85 | -89.66 | -320.53 | 141.21 | 1.114 | 0.024 | 1.127 | 0.014 | -0.013 | -0.051 | 0.025 | 6,977 | SW |
| [2] | 787 | 1069 | 1207.63 | 219.06 | 1883.32 | 154.27 | -675.69 | -1055.22 | -296.17 | 0.094 | 0.003 | 0.115 | 0.002 | -0.021 | -0.049 | 0.008 | 32,645 | SW |
| [3] | 787 | 1069 | 3594.67 | 324.21 | 4842.57 | 209.78 | -1247.90 | -1975.52 | -520.29 | 0.095 | 0.002 | 0.115 | 0.002 | -0.020 | -0.048 | 0.008 | 62,765 | SW |
| [4] | 787 | 838 | 777.70 | 21.09 | 918.12 | 14.62 | -140.41 | -317.18 | 36.36 | 1.091 | 0.003 | 1.118 | 0.002 | -0.027 | -0.056 | 0.002 | 5,149 | SW |
| [5] | 286 | 460 | 784.28 | 17.63 | 762.26 | 12.30 | 22.03 | -207.90 | 251.95 | 1.090 | 0.003 | 1.076 | 0.002 | 0.014 | -0.026 | 0.055 | 1,555 | NE |
| [6] | 501 | 609 | 770.09 | 30.18 | 977.58 | 19.27 | -207.50 | -432.29 | 17.29 | 1.090 | 0.004 | 1.130 | 0.003 | -0.040 | -0.076 | -0.003 | 5,249 | SW |
| [7] | 159 | 196 | 622.02 | 41.45 | 676.04 | 26.21 | -54.02 | -396.19 | 288.15 | 1.065 | 0.006 | 1.082 | 0.003 | -0.016 | -0.070 | 0.038 | 3,344 | SW |
| [8] | 628 | 873 | 793.01 | 21.69 | 931.83 | 13.14 | -138.82 | -325.70 | 48.06 | 1.092 | 0.004 | 1.112 | 0.002 | -0.020 | -0.052 | 0.012 | 6,938 | SW |
| [9] | 357 | 493 | 941.60 | 23.33 | 885.96 | 17.26 | 55.64 | -205.15 | 316.43 | 1.126 | 0.004 | 1.105 | 0.003 | 0.021 | -0.023 | 0.065 | 2,698 | NE |
| [10] | 430 | 576 | 616.46 | 28.91 | 884.05 | 16.36 | -267.58 | -472.56 | -62.61 | 1.055 | 0.004 | 1.108 | 0.002 | -0.052 | -0.086 | -0.019 | 5,105 | SW |
| [11] | 559 | 797 | 732.86 | 16.99 | 727.49 | 10.78 | 5.37 | -175.57 | 186.32 | 1.077 | 0.003 | 1.083 | 0.002 | -0.006 | -0.036 | 0.024 | -861 (dominated) | NW |
| [12] | 228 | 272 | 877.34 | 58.53 | 1346.26 | 38.83 | -468.92 | -826.16 | -111.68 | 1.119 | 0.009 | 1.176 | 0.006 | -0.057 | -0.118 | 0.004 | 8,226 | SW |
| [13] | 746 | 971 | 764.47 | 32.16 | 876.67 | 20.87 | -112.20 | -289.12 | 64.72 | 1.089 | 0.006 | 1.107 | 0.004 | -0.018 | -0.047 | 0.011 | 6,216 | SW |
| [14] | 41 | 98 | 830.83 | 325.89 | 963.81 | 150.68 | -132.99 | -1011.97 | 746.00 | 1.060 | 0.056 | 1.103 | 0.029 | -0.043 | -0.196 | 0.110 | 3,076 | SW |

ICER: incremental cost-effectiveness ratio, CI: confidence interval, NE: north-east, NW: north-west, SE (under ICER quadrant): south-east, SE: standard error, SW: south-west

[1] Sensitivity analysis: Complete case analysis. Adjusted for age category, comorbidity, duration of illness and vaccination status.

[2] Sensitivity analysis: 12-month time horizon. Adjusted for age category, comorbidity, duration of illness and vaccination status.

[3] Sensitivity analysis: Societal perspective. Adjusted for age category, comorbidity, duration of illness and vaccination status.

[4] Sensitivity analysis: Concurrent randomisation analysis population. Adjusted for age category, comorbidity, duration of illness and vaccination status.

[5] Subgroup analysis: Aged 50-64 years. Adjusted for duration of illness and vaccination status.

[6] Subgroup analysis: Aged ≥65 years. Adjusted for comorbidity, duration of illness and vaccination status.

[7] Subgroup analysis: No comorbidity at baseline. Adjusted for duration of illness and vaccination status.

[8] Subgroup analysis: Had comorbidity at baseline. Adjusted for age category, duration of illness and vaccination status.

[9] Subgroup analysis: Duration of symptoms of <7 days before randomisation. Adjusted for age category, comorbidity and vaccination status.

[10] Subgroup analysis: Duration of symptoms of ≥7 days before randomisation. Adjusted for age category, comorbidity and vaccination status.

[11] Subgroup analysis: No symptoms rated as 'major' at baseline. Adjusted for age category, comorbidity, duration of illness and vaccination status.

[12] Subgroup analysis: At least one symptom rated as 'major' at baseline. Adjusted for age category, comorbidity, duration of illness and vaccination status.

[13] Subgroup analysis: Did not use inhaled corticosteroid steroid at randomisation/within 28 days follow-up. Adjusted for age category, comorbidity, duration of illness and vaccination status.

[14] Subgroup analysis: Used inhaled corticosteroid steroid at randomisation/within 28 days follow-up. Adjusted for age category, comorbidity, duration of illness and vaccination status.

## Table S25. Sensitivity and subgroup analyses of the cost-effectiveness analysis in terms of incremental cost per hospitalisation or death avoided: Usual care vs. Colchicine

|  | **Number** | | **Cost** | | | | | | | **Hospitalisation or death** | | | | | | | **ICER** | **ICER quadrant** |
| --- | --- | --- | --- | --- | --- | --- | --- | --- | --- | --- | --- | --- | --- | --- | --- | --- | --- | --- |
|  | **Colchicine** | **Usual care** | **Colchicine** | | **Usual care** | | **Mean difference** | **Lower 95%CI** | **Upper 95%CI** | **Colchicine** | | **Usual care** | | **Mean difference** | **Lower 95%CI** | **Upper 95%CI** |  |  |
|  |  |  | **Mean** | **SE** | **Mean** | **SE** |  |  |  | **Mean** | **SE** | **Mean** | **SE** |  |  |  |  |  |
| [1] | 156 | 590 | 868.67 | 156.48 | 897.70 | 81.78 | -29.04 | -290.38 | 232.31 | 1.087 | 0.025 | 1.119 | 0.013 | -0.032 | -0.073 | 0.010 | 913 | SW |
| [2] | 156 | 1145 | 1227.82 | 175.07 | 1868.31 | 124.44 | -640.48 | -1114.02 | -166.94 | 0.090 | 0.003 | 0.112 | 0.002 | -0.022 | -0.063 | 0.019 | 29,233 | SW |
| [3] | 156 | 1145 | 3511.22 | 252.34 | 5044.93 | 145.01 | -1533.71 | -2846.33 | -221.10 | 0.089 | 0.004 | 0.112 | 0.002 | -0.023 | -0.064 | 0.018 | 67,106 | SW |
| [4] | 156 | 133 | 542.03 | 10.03 | 341.40 | 10.03 | 200.63 | -27.92 | 429.18 | 1.035 | 0.0013 | 1.030 | 0.0013 | 0.005 | -0.040 | 0.050 | 39,892 | NE |
| [5] | 132 | 528 | 627.71 | 17.59 | 698.21 | 8.43 | -70.50 | -331.92 | 190.92 | 1.046 | 0.003 | 1.072 | 0.002 | -0.026 | -0.066 | 0.014 | 2,685 | SW |
| [6] | 24 | 617 | 699.90 | 46.34 | 981.12 | 21.34 | -281.22 | -758.26 | 195.81 | 1.065 | 0.007 | 1.130 | 0.003 | -0.065 | -0.154 | 0.025 | 4,347 | SW |
| [7] | 3 | 198 | - | - | - | - | - | - | - | - | - | - | - | - | - | - | - | - |
| [8] | 153 | 947 | 812.78 | 19.89 | 888.54 | 11.99 | -75.76 | -313.80 | 162.28 | 1.079 | 0.003 | 1.108 | 0.002 | -0.028 | -0.066 | 0.009 | 2,668 | SW |
| [9] | 98 | 548 | 732.30 | 27.72 | 836.23 | 12.07 | -103.93 | -427.53 | 219.67 | 1.067 | 0.005 | 1.102 | 0.002 | -0.035 | -0.084 | 0.014 | 2,949 | SW |
| [10] | 58 | 597 | 795.21 | 25.95 | 863.91 | 19.80 | -68.70 | -385.76 | 248.36 | 1.081 | 0.004 | 1.104 | 0.003 | -0.023 | -0.077 | 0.031 | 3,003 | SW |
| [11] | 86 | 829 | 566.79 | 25.37 | 717.64 | 8.74 | -150.85 | -421.69 | 119.99 | 1.039 | 0.004 | 1.082 | 0.001 | -0.043 | -0.075 | -0.011 | 3,518 | SW |
| [12] | 70 | 316 | 1144.96 | 53.50 | 1199.64 | 40.36 | -54.68 | -479.69 | 370.32 | 1.132 | 0.007 | 1.158 | 0.005 | -0.026 | -0.105 | 0.052 | 2,094 | SW |
| [13] | 117 | 1006 | 643.66 | 41.52 | 823.55 | 27.51 | -179.89 | -396.49 | 36.71 | 1.065 | 0.008 | 1.101 | 0.005 | -0.036 | -0.073 | 0.001 | 5,023 | SW |
| [14] | 39 | 139 | 1358.02 | 232.10 | 1026.88 | 161.97 | 331.14 | -397.56 | 1059.85 | 1.117 | 0.039 | 1.116 | 0.029 | 0.002 | -0.112 | 0.115 | 183,501 | NE |

ICER: incremental cost-effectiveness ratio, CI: confidence interval, NE: north-east, NW: north-west, SE (under ICER quadrant): south-east, SE: standard error, SW: south-west

[1] Sensitivity analysis: Complete case analysis. Adjusted for age category, comorbidity, duration of illness and vaccination status.

[2] Sensitivity analysis: 12-month time horizon. Adjusted for age category, comorbidity, duration of illness and vaccination status.

[3] Sensitivity analysis: Societal perspective. Adjusted for age category, comorbidity, duration of illness and vaccination status.

[4] Sensitivity analysis: Concurrent randomisation analysis population. Adjusted for age category, comorbidity, duration of illness and vaccination status.

[5] Subgroup analysis: Aged 18-64 years. Adjusted for duration of illness and vaccination status.

[6] Subgroup analysis: Aged ≥65 years. Adjusted for comorbidity, duration of illness and vaccination status.

[7] Subgroup analysis: No comorbidity at baseline – numbers were too small for computation.

[8] Subgroup analysis: Had comorbidity at baseline. Adjusted for age category, duration of illness and vaccination status.

[9] Subgroup analysis: Duration of symptoms of <7 days before randomisation. Adjusted for age category, comorbidity and vaccination status.

[10] Subgroup analysis: Duration of symptoms of ≥7 days before randomisation. Adjusted for age category, comorbidity and vaccination status.

[11] Subgroup analysis: No symptoms rated as 'major' at baseline. Adjusted for age category, comorbidity, duration of illness and vaccination status.

[12] Subgroup analysis: At least one symptom rated as 'major' at baseline. Adjusted for age category, comorbidity, duration of illness and vaccination status.

[13] Subgroup analysis: Did not use inhaled corticosteroid steroid at randomisation/within 28 days follow-up. Adjusted for age category, comorbidity, duration of illness and vaccination status.

[14] Subgroup analysis: Used inhaled corticosteroid steroid at randomisation/within 28 days follow-up. Adjusted for age category, comorbidity, duration of illness and vaccination status.

## Table S26. Sensitivity and subgroup analyses of the cost-effectiveness analysis in terms of incremental cost per hospitalisation or death avoided: Usual care vs. Favipiravir

|  | **Number** | | **Cost** | | | | | | | **Hospitalisation or death** | | | | | | | **ICER** | **ICER quadrant** |
| --- | --- | --- | --- | --- | --- | --- | --- | --- | --- | --- | --- | --- | --- | --- | --- | --- | --- | --- |
|  | **Favipiravir** | **Usual care** | **Favipiravir** | | **Usual care** | | **Mean difference** | **Lower 95%CI** | **Upper 95%CI** | **Favipiravir** | | **Usual care** | | **Mean difference** | **Lower 95%CI** | **Upper 95%CI** |  |  |
|  |  |  | **Mean** | **SE** | **Mean** | **SE** |  |  |  | **Mean** | **SE** | **Mean** | **SE** |  |  |  |  |  |
| [1] | 1829 | 2704 | 539.47 | 32.94 | 492.88 | 22.91 | 46.58 | 0.23 | 92.94 | 1.031 | 0.006 | 1.037 | 0.004 | -0.006 | -0.014 | 0.003 | -8,274 (dominated) | NW |
| [2] | 1829 | 3256 | 821.69 | 27.00 | 976.95 | 22.71 | -155.27 | -251.15 | -59.38 | 0.043 | 0.001 | 0.055 | 0.001 | -0.012 | -0.022 | -0.002 | 12,762 | SW |
| [3] | 1829 | 3256 | 2217.84 | 74.14 | 2952.13 | 56.04 | -734.28 | -1055.35 | -413.22 | 0.043 | 0.001 | 0.055 | 0.001 | -0.012 | -0.022 | -0.002 | 59,984 | SW |
| [4] | 1829 | 1668 | 449.12 | 4.71 | 385.06 | 4.71 | 64.06 | 16.42 | 111.70 | 1.016 | 0.0004 | 1.014 | 0.0004 | 0.002 | -0.006 | 0.010 | 28,798 | NE |
| [5] | 1524 | 2286 | 498.44 | 4.95 | 437.20 | 4.31 | 61.24 | 10.52 | 111.96 | 1.022 | 0.001 | 1.028 | 0.001 | -0.006 | -0.015 | 0.003 | -10,244 (dominated) | NW |
| [6] | 305 | 970 | 711.59 | 22.90 | 787.05 | 17.68 | -75.47 | -212.50 | 61.57 | 1.064 | 0.004 | 1.086 | 0.003 | -0.021 | -0.045 | 0.002 | 3,513 | SW |
| [7] | 601 | 890 | 299.29 | 7.95 | 279.01 | 7.57 | 20.28 | -25.59 | 66.15 | 1.018 | 0.001 | 1.024 | 0.001 | -0.005 | -0.015 | 0.004 | -3,774 (dominated) | NW |
| [8] | 1228 | 2366 | 674.22 | 7.29 | 640.14 | 5.96 | 34.08 | -32.79 | 100.96 | 1.041 | 0.001 | 1.053 | 0.001 | -0.011 | -0.023 | 0.000 | -2,986 (dominated) | NW |
| [9] | 1347 | 2087 | 487.21 | 6.40 | 477.29 | 5.62 | 9.92 | -41.74 | 61.57 | 1.027 | 0.001 | 1.037 | 0.001 | -0.010 | -0.020 | 0.000 | -998 (dominated) | NW |
| [10] | 482 | 1169 | 748.53 | 14.51 | 655.93 | 11.34 | 92.60 | -19.98 | 205.18 | 1.053 | 0.002 | 1.059 | 0.002 | -0.006 | -0.022 | 0.010 | -16,315 (dominated) | NW |
| [11] | 1193 | 2234 | 497.06 | 6.47 | 490.37 | 5.18 | 6.69 | -52.79 | 66.17 | 1.025 | 0.001 | 1.037 | 0.001 | -0.012 | -0.021 | -0.003 | -551 (dominated) | NW |
| [12] | 636 | 1022 | 710.53 | 15.71 | 653.03 | 14.47 | 57.51 | -30.07 | 145.09 | 1.054 | 0.003 | 1.063 | 0.003 | -0.008 | -0.026 | 0.009 | -7,033 (dominated) | NW |
| [13] | 1478 | 2642 | 437.88 | 13.81 | 400.56 | 11.42 | 37.32 | -15.21 | 89.85 | 1.032 | 0.003 | 1.036 | 0.002 | -0.004 | -0.013 | 0.006 | -10,286 (dominated) | NW |
| [14] | 351 | 614 | 1118.15 | 55.91 | 1126.08 | 48.54 | -7.93 | -142.11 | 126.26 | 1.050 | 0.011 | 1.082 | 0.009 | -0.032 | -0.057 | -0.008 | 245 | SW |

ICER: incremental cost-effectiveness ratio, CI: confidence interval, NE: north-east, NW: north-west, SE (under ICER quadrant): south-east, SE: standard error, SW: south-west

[1] Sensitivity analysis: Complete case analysis. Adjusted for age category, comorbidity, duration of illness and vaccination status.

[2] Sensitivity analysis: 12-month time horizon. Adjusted for age category, comorbidity, duration of illness and vaccination status.

[3] Sensitivity analysis: Societal perspective. Adjusted for age category, comorbidity, duration of illness and vaccination status.

[4] Sensitivity analysis: Concurrent randomisation analysis population. Adjusted for age category, comorbidity, duration of illness and vaccination status.

[5] Subgroup analysis: Aged 18-64 years. Adjusted for comorbidity, duration of illness and vaccination status.

[6] Subgroup analysis: Aged ≥65 years. Adjusted for comorbidity, duration of illness and vaccination status.

[7] Subgroup analysis: No comorbidity at baseline. Adjusted for age category, duration of illness and vaccination status.

[8] Subgroup analysis: Had comorbidity at baseline. Adjusted for age category, duration of illness and vaccination status.

[9] Subgroup analysis: Duration of symptoms of <7 days before randomisation. Adjusted for age category, comorbidity and vaccination status.

[10] Subgroup analysis: Duration of symptoms of ≥7 days before randomisation. Adjusted for age category, comorbidity and vaccination status.

[11] Subgroup analysis: No symptoms rated as 'major' at baseline. Adjusted for age category, comorbidity, duration of illness and vaccination status.

[12] Subgroup analysis: At least one symptom rated as 'major' at baseline. Adjusted for age category, comorbidity, duration of illness and vaccination status.

[13] Subgroup analysis: Did not use inhaled corticosteroid steroid at randomisation/within 28 days follow-up. Adjusted for age category, comorbidity, duration of illness and vaccination status.

[14] Subgroup analysis: Used inhaled corticosteroid steroid at randomisation/within 28 days follow-up. Adjusted for age category, comorbidity, duration of illness and vaccination status.

## Table S27. Sensitivity and subgroup analyses of the cost-effectiveness analysis in terms of incremental cost per hospitalisation or death avoided: Usual care vs. Ivermectin

|  | **Number** | | **Cost** | | | | | | | **Hospitalisation or death** | | | | | | | **ICER*** | **ICER quadrant** |
| --- | --- | --- | --- | --- | --- | --- | --- | --- | --- | --- | --- | --- | --- | --- | --- | --- | --- | --- |
|  | **Ivermectin** | **Usual care** | **Ivermectin** | | **Usual care** | | **Mean difference** | **Lower 95%CI** | **Upper 95%CI** | **Ivermectin** | | **Usual care** | | **Mean difference** | **Lower 95%CI** | **Upper 95%CI** |  |  |
|  |  |  | **Mean** | **SE** | **Mean** | **SE** |  |  |  | **Mean** | **SE** | **Mean** | **SE** |  |  |  |  |  |
| [1] | 2157 | 2704 | 1075.68 | 31.55 | 490.38 | 22.65 | 585.30 | 542.29 | 628.31 | 1.027 | 0.006 | 1.037 | 0.004 | -0.009 | -0.017 | -0.001 | -63,130 | NW |
| [2] | 2157 | 3256 | 1331.39 | 22.56 | 972.22 | 17.53 | 359.17 | 276.21 | 442.14 | 0.041 | 0.001 | 0.055 | 0.001 | -0.014 | -0.024 | -0.005 | -25,010 | NW |
| [3] | 2157 | 3256 | 2799.27 | 45.55 | 2968.52 | 37.00 | -169.25 | -466.57 | 128.07 | 0.041 | 0.001 | 0.055 | 0.001 | -0.014 | -0.024 | -0.005 | 11,929 | SW |
| [4] | 2157 | 1806 | 1000.94 | 25.90 | 387.21 | 15.68 | 613.74 | 573.36 | 654.11 | 1.015 | 0.005 | 1.015 | 0.003 | -0.0001 | -0.008 | 0.008 | -11,507,890 | NW |
| [5] | 1811 | 2286 | 1039.28 | 4.87 | 437.19 | 4.16 | 602.09 | 559.18 | 645.00 | 1.022 | 0.001 | 1.028 | 0.001 | -0.006 | -0.014 | 0.003 | -104,618 | NW |
| [6] | 346 | 970 | 1261.21 | 31.10 | 787.02 | 16.69 | 474.19 | 333.57 | 614.81 | 1.049 | 0.005 | 1.086 | 0.002 | -0.037 | -0.058 | -0.016 | -12,815 | NW |
| [7] | 697 | 890 | 822.98 | 7.90 | 279.01 | 7.69 | 543.97 | 489.59 | 598.35 | 1.018 | 0.001 | 1.024 | 0.001 | -0.006 | -0.016 | 0.004 | -89,475 | NW |
| [8] | 1460 | 2366 | 1214.20 | 9.50 | 640.11 | 5.33 | 574.09 | 514.87 | 633.30 | 1.037 | 0.001 | 1.053 | 0.001 | -0.016 | -0.027 | -0.006 | -35,852 | NW |
| [9] | 1556 | 2087 | 1031.58 | 7.12 | 477.28 | 5.63 | 554.30 | 504.63 | 603.98 | 1.020 | 0.001 | 1.037 | 0.001 | -0.016 | -0.025 | -0.008 | -33,621 | NW |
| [10] | 601 | 1169 | 1259.35 | 13.26 | 655.90 | 10.07 | 603.45 | 511.20 | 695.69 | 1.056 | 0.002 | 1.059 | 0.002 | -0.003 | -0.020 | 0.014 | -209,188 | NW |
| [11] | 1418 | 2234 | 1074.47 | 8.86 | 490.37 | 5.36 | 584.09 | 531.92 | 636.27 | 1.026 | 0.001 | 1.037 | 0.001 | -0.011 | -0.020 | -0.002 | -52,639 | NW |
| [12] | 739 | 1022 | 1177.74 | 13.12 | 652.96 | 12.60 | 524.79 | 443.47 | 606.11 | 1.043 | 0.002 | 1.063 | 0.002 | -0.019 | -0.035 | -0.004 | -27,344 | NW |
| [13] | 1737 | 2625 | 959.61 | 11.94 | 392.89 | 9.86 | 566.71 | 523.67 | 609.75 | 1.025 | 0.002 | 1.035 | 0.002 | -0.009 | -0.017 | -0.001 | -60,396 | NW |
| [14] | 420 | 631 | 1688.58 | 46.26 | 1146.78 | 35.21 | 541.79 | 396.97 | 686.61 | 1.060 | 0.009 | 1.086 | 0.007 | -0.026 | -0.052 | -0.0004 | -20,797 | NW |

ICER: incremental cost-effectiveness ratio, CI: confidence interval, NE: north-east, NW: north-west, SE (under ICER quadrant): south-east, SE: standard error, SW: south-west

* All dominated except [3]

[1] Sensitivity analysis: Complete case analysis. Adjusted for age category, comorbidity, duration of illness and vaccination status.

[2] Sensitivity analysis: 12-month time horizon. Adjusted for age category, comorbidity, duration of illness and vaccination status.

[3] Sensitivity analysis: Societal perspective. Adjusted for age category, comorbidity, duration of illness and vaccination status.

[4] Sensitivity analysis: Concurrent randomisation analysis population. Adjusted for age category, comorbidity, duration of illness and vaccination status.

[5] Subgroup analysis: Aged 18-64 years. Adjusted for comorbidity, duration of illness and vaccination status.

[6] Subgroup analysis: Aged ≥65 years. Adjusted for comorbidity, duration of illness and vaccination status.

[7] Subgroup analysis: No comorbidity at baseline. Adjusted for age category, duration of illness and vaccination status.

[8] Subgroup analysis: Had comorbidity at baseline. Adjusted for age category, duration of illness and vaccination status.

[9] Subgroup analysis: Duration of symptoms of <7 days before randomisation. Adjusted for age category, comorbidity and vaccination status.

[10] Subgroup analysis: Duration of symptoms of ≥7 days before randomisation. Adjusted for age category, comorbidity and vaccination status.

[11] Subgroup analysis: No symptoms rated as 'major' at baseline. Adjusted for age category, comorbidity, duration of illness and vaccination status.

[12] Subgroup analysis: At least one symptom rated as 'major' at baseline. Adjusted for age category, comorbidity, duration of illness and vaccination status.

[13] Subgroup analysis: Did not use inhaled corticosteroid steroid at randomisation/within 28 days follow-up. Adjusted for age category, comorbidity, duration of illness and vaccination status.

[14] Subgroup analysis: Used inhaled corticosteroid steroid at randomisation/within 28 days follow-up. Adjusted for age category, comorbidity, duration of illness and vaccination status.

## Table S28. Sensitivity and subgroup analyses of the cost-effectiveness analysis in terms of incremental cost per day saved until recovery: Usual care vs. Azithromycin

|  | **Number** | | **Cost** | | | | | | | **Time-to-recovery** | | | | | | | **ICER** | **ICER quadrant** |
| --- | --- | --- | --- | --- | --- | --- | --- | --- | --- | --- | --- | --- | --- | --- | --- | --- | --- | --- |
|  | **Azithromycin** | **Usual care** | **Azithromycin** | | **Usual care** | | **Mean difference** | **Lower 95%CI** | **Upper 95%CI** | **Azithromycin** | | **Usual care** | | **Mean difference** | **Lower 95%CI** | **Upper 95%CI** |  |  |
|  |  |  | **Mean** | **SE** | **Mean** | **SE** |  |  |  | **Mean** | **SE** | **Mean** | **SE** |  |  |  |  |  |
| [1] | 186 | 236 | 660.50 | 183.76 | 693.53 | 98.58 | -33.03 | -336.84 | 270.78 | 13.23 | 1.23 | 14.95 | 0.67 | 1.72 | -0.31 | 3.75 | -19 | SE |
| [2] | 186 | 225 | 712.90 | 201.35 | 703.68 | 103.25 | 9.22 | -329.41 | 347.84 | 14.25 | 1.55 | 15.39 | 0.84 | 1.14 | -1.42 | 3.69 | 8 | NE |
| [3] | 108 | 151 | 575.18 | 379.16 | 906.36 | 226.65 | -331.17 | -926.63 | 264.28 | 11.79 | 1.87 | 14.27 | 1.04 | 2.48 | -0.57 | 5.53 | -134 | SE |
| [4] | 78 | 85 | 274.36 | 577.57 | 663.89 | 402.93 | -389.53 | -1200.17 | 421.11 | 13.81 | 3.30 | 15.88 | 1.84 | 2.07 | -3.30 | 7.44 | -188 | SE |
| [5] | 29 | 29 | 721.80 | 197.33 | 795.68 | 107.78 | -73.88 | -397.69 | 249.93 | 13.06 | 1.23 | 14.94 | 0.66 | 1.88 | -0.15 | 3.92 | -39 | SE |
| [6] | 157 | 207 | 836.62 | 341.69 | 991.78 | 192.44 | -155.16 | -708.28 | 397.95 | 13.60 | 1.84 | 15.44 | 0.95 | 1.84 | -1.25 | 4.93 | -84 | SE |
| [7] | 75 | 96 | 523.82 | 206.90 | 632.26 | 110.14 | -108.44 | -451.54 | 234.66 | 12.87 | 1.54 | 14.85 | 0.87 | 1.98 | -0.50 | 4.47 | -55 | SE |
| [8] | 111 | 140 | 617.96 | 199.55 | 722.73 | 107.04 | -104.77 | -434.69 | 225.15 | 12.30 | 1.29 | 14.86 | 0.69 | 2.56 | 0.43 | 4.69 | -41 | SE |
| [9] | 143 | 198 | 782.43 | 488.21 | 1052.72 | 319.46 | -270.29 | -993.50 | 452.93 | 16.24 | 2.67 | 15.68 | 1.50 | -0.56 | -4.89 | 3.77 | 482 | SW |
| [10] | 43 | 38 | 660.50 | 183.76 | 693.53 | 98.58 | -33.03 | -336.84 | 270.78 | 13.23 | 1.23 | 14.95 | 0.67 | 1.72 | -0.31 | 3.75 | -19 | SE |
| [11] | 0 | 0 | - | - | - | - | - | - | - | - | - | - | - | - | - | - | - |  |
| [12] | 0 | 0 | - | - | - | - | - | - | - | - | - | - | - | - | - | - | - |  |

ICER: incremental cost-effectiveness ratio, CI: confidence interval, NE: north-east, NW: north-west, SE (under ICER quadrant): south-east, SE: standard error, SW: south-west

[1] Sensitivity analysis: Complete case analysis – same as the base case results because there were no missing data in the base case analysis. Adjusted for age category, comorbidity and duration of illness.

[2] Sensitivity analysis: Concurrent randomisation analysis population. Adjusted for age category, comorbidity and duration of illness.

[3] Subgroup analysis: Aged 50-64 years. Adjusted for duration of illness.

[4] Subgroup analysis: Aged ≥65 years. Adjusted for comorbidity and duration of illness.

[5] Subgroup analysis: No comorbidity at baseline. Adjusted for duration of illness.

[6] Subgroup analysis: Had comorbidity at baseline. Adjusted for age category and duration of illness.

[7] Subgroup analysis: Duration of symptoms of <7 days before randomisation. Adjusted for age category and comorbidity.

[8] Subgroup analysis: Duration of symptoms of ≥7 days before randomisation. Adjusted for age category and comorbidity.

[9] Subgroup analysis: No symptoms rated as 'major' at baseline. Adjusted for age category, comorbidity and duration of illness.

[10] Subgroup analysis: At least one symptom rated as 'major' at baseline. Adjusted for age category and duration of illness.

[11] Subgroup analysis: Did not use inhaled corticosteroid steroid at randomisation/within 28 days follow-up.

[12] Subgroup analysis: Used inhaled corticosteroid steroid at randomisation/within 28 days follow-up.

## Table S29. Sensitivity and subgroup analyses of the cost-effectiveness analysis in terms of incremental cost per day saved until recovery: Usual care vs. Doxycycline

|  | **Number** | | **Cost** | | | | | | | **Time-to-recovery** | | | | | | | **ICER** | **ICER quadrant** |
| --- | --- | --- | --- | --- | --- | --- | --- | --- | --- | --- | --- | --- | --- | --- | --- | --- | --- | --- |
|  | **Doxycycline** | **Usual care** | **Doxycycline** | | **Usual care** | | **Mean difference** | **Lower 95%CI** | **Upper 95%CI** | **Doxycycline** | | **Usual care** | | **Mean difference** | **Lower 95%CI** | **Upper 95%CI** |  |  |
|  |  |  | **Mean** | **SE** | **Mean** | **SE** |  |  |  | **Mean** | **SE** | **Mean** | **SE** |  |  |  |  |  |
| [1] | 435 | 336 | 746.68 | 155.85 | 897.99 | 98.44 | -151.31 | -388.00 | 85.38 | 14.53 | 0.93 | 15.25 | 0.58 | 0.72 | -0.71 | 2.15 | -210 | SE |
| [2] | 435 | 321 | 747.13 | 74.17 | 828.36 | 94.58 | -81.24 | -316.68 | 154.21 | 14.77 | 0.48 | 15.50 | 0.55 | 0.73 | -0.66 | 2.13 | -111 | SE |
| [3] | 248 | 189 | 706.03 | 85.30 | 678.27 | 87.64 | 27.76 | -212.94 | 268.45 | 15.04 | 0.60 | 16.04 | 0.73 | 1.00 | -0.80 | 2.79 | 28 | NE |
| [4] | 187 | 147 | 801.64 | 127.95 | 1171.43 | 187.62 | -369.79 | -815.16 | 75.58 | 14.50 | 0.78 | 14.75 | 0.78 | 0.25 | -1.89 | 2.39 | -1,506 | SE |
| [5] | 63 | 51 | 493.83 | 158.70 | 749.37 | 265.89 | -255.53 | -858.37 | 347.30 | 13.18 | 1.38 | 14.91 | 1.37 | 1.73 | -2.02 | 5.48 | -148 | SE |
| [6] | 372 | 285 | 790.02 | 82.16 | 919.91 | 103.62 | -129.89 | -389.48 | 129.71 | 15.07 | 0.51 | 15.54 | 0.58 | 0.47 | -1.02 | 1.97 | -274 | SE |
| [7] | 194 | 145 | 842.09 | 114.61 | 858.36 | 141.83 | -16.27 | -374.82 | 342.27 | 14.76 | 0.67 | 15.55 | 0.79 | 0.79 | -1.22 | 2.79 | -21 | SE |
| [8] | 241 | 191 | 670.68 | 95.19 | 921.10 | 131.37 | -250.41 | -569.94 | 69.11 | 14.79 | 0.68 | 15.37 | 0.72 | 0.57 | -1.34 | 2.49 | -436 | SE |
| [9] | 346 | 272 | 690.80 | 76.99 | 767.49 | 97.44 | -76.70 | -318.89 | 165.50 | 14.38 | 0.53 | 15.32 | 0.61 | 0.94 | -0.61 | 2.49 | -82 | SE |
| [10] | 89 | 64 | 966.12 | 194.42 | 1431.79 | 284.30 | -465.66 | -1144.36 | 213.03 | 16.52 | 1.14 | 15.99 | 1.10 | -0.53 | -3.54 | 2.49 | 881 | SW |
| [11] | 344 | 242 | 709.53 | 93.84 | 830.63 | 122.91 | -121.09 | -409.79 | 167.60 | 15.02 | 0.61 | 15.94 | 0.68 | 0.92 | -0.80 | 2.64 | -132 | SE |
| [12] | 91 | 94 | 861.47 | 228.03 | 1058.14 | 249.62 | -196.67 | -813.33 | 419.99 | 13.92 1 | 1.22 | 14.23 9 | 1.20 7 | 0.31 7 | -2.913 | 3.54 7 | -620 | SE |

ICER: incremental cost-effectiveness ratio, CI: confidence interval, NE: north-east, NW: north-west, SE (under ICER quadrant): south-east, SE: standard error, SW: south-west

[1] Sensitivity analysis: Complete case analysis. Adjusted for age category, comorbidity and duration of illness.

[2] Sensitivity analysis: Concurrent randomisation analysis population. Adjusted for age category, comorbidity and duration of illness.

[3] Subgroup analysis: Aged 50-64 years. Adjusted for duration of illness.

[4] Subgroup analysis: Aged ≥65 years. Adjusted for comorbidity and duration of illness.

[5] Subgroup analysis: No comorbidity at baseline. Adjusted for duration of illness.

[6] Subgroup analysis: Had comorbidity at baseline. Adjusted for age category and duration of illness.

[7] Subgroup analysis: Duration of symptoms of <7 days before randomisation. Adjusted for age category and comorbidity.

[8] Subgroup analysis: Duration of symptoms of ≥7 days before randomisation. Adjusted for age category and comorbidity.

[9] Subgroup analysis: No symptoms rated as 'major' at baseline. Adjusted for age category, comorbidity and duration of illness.

[10] Subgroup analysis: At least one symptom rated as 'major' at baseline. Adjusted for age category, comorbidity and duration of illness.

[11] Subgroup analysis: Did not use inhaled corticosteroid steroid at randomisation/within 28 days follow-up. Adjusted for age category, comorbidity and duration of illness.

[12] Subgroup analysis: Used inhaled corticosteroid steroid at randomisation/within 28 days follow-up. Adjusted for age category, comorbidity and duration of illness.

## Table S30. Sensitivity and subgroup analyses of the cost-effectiveness analysis in terms of incremental cost per day saved until recovery: Usual care vs. Budesonide

|  | **Number** | | **Cost** | | | | | | | **Time-to-recovery** | | | | | | | **ICER** | **ICER quadrant** |
| --- | --- | --- | --- | --- | --- | --- | --- | --- | --- | --- | --- | --- | --- | --- | --- | --- | --- | --- |
|  | **Budesonide** | **Usual care** | **Budesonide** | | **Usual care** | | **Mean difference** | **Lower 95%CI** | **Upper 95%CI** | **Budesonide** | | **Usual care** | | **Mean difference** | **Lower 95%CI** | **Upper 95%CI** |  |  |
|  |  |  | **Mean** | **SE** | **Mean** | **SE** |  |  |  | **Mean** | **SE** | **Mean** | **SE** |  |  |  |  |  |
| [1] | 476 | 516 | 873.24 | 153.91 | 968.13 | 93.87 | -94.89 | -333.83 | 144.05 | 14.67 | 0.78 | 16.41 | 0.44 | 1.74 | 0.48 | 2.99 | -55 | SE |
| [2] | 787 | 838 | 766.31 | 58.17 | 918.12 | 70.00 | -151.80 | -330.31 | 26.71 | 14.52 | 0.35 | 16.35 | 0.34 | 1.83 | 0.89 | 2.77 | -83 | SE |
| [3] | 286 | 460 | 784.28 | 93.96 | 762.26 | 72.61 | 22.02 | -210.44 | 254.48 | 15.10 | 0.56 | 16.57 | 0.46 | 1.48 | 0.08 | 2.87 | 15 | NE |
| [4] | 501 | 609 | 756.06 | 74.72 | 977.58 | 88.15 | -221.52 | -448.67 | 5.62 | 14.14 | 0.44 | 15.66 | 0.41 | 1.52 | 0.35 | 2.69 | -146 | SE |
| [5] | 159 | 196 | 608.03 | 111.18 | 676.04 | 134.03 | -68.00 | -407.67 | 271.66 | 13.48 | 0.76 | 15.08 | 0.71 | 1.60 | -0.42 | 3.62 | -42 | SE |
| [6] | 628 | 873 | 806.39 | 67.58 | 931.83 | 64.97 | -125.44 | -310.32 | 59.44 | 14.75 | 0.39 | 16.26 | 0.33 | 1.51 | 0.53 | 2.49 | -83 | SE |
| [7] | 357 | 493 | 958.19 | 103.81 | 885.96 | 85.62 | 72.23 | -190.94 | 335.40 | 15.17 | 0.51 | 15.86 | 0.43 | 0.69 | -0.60 | 1.98 | 105 | NE |
| [8] | 430 | 576 | 607.02 | 63.75 | 884.05 | 80.73 | -277.03 | -478.64 | -75.42 | 13.93 | 0.47 | 16.20 | 0.42 | 2.27 | 1.05 | 3.49 | -122 | SE |
| [9] | 559 | 797 | 727.54 | 68.09 | 727.49 | 61.25 | 0.05 | -179.00 | 179.11 | 13.93 | 0.41 | 15.44 | 0.35 | 1.51 | 0.46 | 2.56 | 0.04 | NE |
| [10] | 228 | 272 | 861.38 | 111.51 | 1346.26 | 147.02 | -484.87 | -848.18 | -121.56 | 15.91 | 0.63 | 17.84 | 0.59 | 1.93 | 0.27 | 3.58 | -252 | SE |
| [11] | 731 | 977 | 763.59 | 60.93 | 870.40 | 64.19 | -106.81 | -277.77 | 64.15 | 14.49 | 0.37 | 16.04 | 0.33 | 1.54 | 0.61 | 2.48 | -69 | SE |
| [12] | 56 | 92 | 821.61 | 300.38 | 1044.06 | 300.82 | -222.45 | -961.09 | 516.20 | 14.49 | 2.33 | 16.07 | 1.36 | 1.59 | -2.89 | 6.06 | -140 | SE |

ICER: incremental cost-effectiveness ratio, CI: confidence interval, NE: north-east, NW: north-west, SE (under ICER quadrant): south-east, SE: standard error, SW: south-west

[1] Sensitivity analysis: Complete case analysis. Adjusted for age category, comorbidity, duration of illness and vaccination status.

[2] Sensitivity analysis: Concurrent randomisation analysis population. Adjusted for age category, comorbidity, duration of illness and vaccination status.

[3] Subgroup analysis: Aged 50-64 years. Adjusted for duration of illness and vaccination status.

[4] Subgroup analysis: Aged ≥65 years. Adjusted for comorbidity, duration of illness and vaccination status.

[5] Subgroup analysis: No comorbidity at baseline. Adjusted for duration of illness and vaccination status.

[6] Subgroup analysis: Had comorbidity at baseline. Adjusted for comorbidity, duration of illness and vaccination status.

[7] Subgroup analysis: Duration of symptoms of <7 days before randomisation. Adjusted for age category, comorbidity and vaccination status.

[8] Subgroup analysis: Duration of symptoms of ≥7 days before randomisation. Adjusted for age category, comorbidity and vaccination status.

[9] Subgroup analysis: No symptoms rated as 'major' at baseline. Adjusted for age category, comorbidity, duration of illness and vaccination status.

[10] Subgroup analysis: At least one symptom rated as 'major' at baseline. Adjusted for age category, comorbidity, duration of illness and vaccination status.

[11] Subgroup analysis: Did not use inhaled corticosteroid steroid at randomisation/within 28 days follow-up. Adjusted for age category, comorbidity, duration of illness and vaccination status.

[12] Subgroup analysis: Used inhaled corticosteroid steroid at randomisation/within 28 days follow-up. Adjusted for age category, comorbidity, duration of illness and vaccination status.

## Table S31. Sensitivity and subgroup analyses of the cost-effectiveness analysis in terms of incremental cost per day saved until recovery: Usual care vs. Colchicine

|  | **Number** | | **Cost** | | | | | | | **Time-to-recovery** | | | | | | | **ICER** | **ICER quadrant** |
| --- | --- | --- | --- | --- | --- | --- | --- | --- | --- | --- | --- | --- | --- | --- | --- | --- | --- | --- |
|  | **Colchicine** | **Usual care** | **Colchicine** | | **Usual care** | | **Mean difference** | **Lower 95%CI** | **Upper 95%CI** | **Colchicine** | | **Usual care** | | **Mean difference** | **Lower 95%CI** | **Upper 95%CI** |  |  |
|  |  |  | **Mean** | **SE** | **Mean** | **SE** |  |  |  | **Mean** | **SE** | **Mean** | **SE** |  |  |  |  |  |
| [1] | 156 | 590 | 558.14 | 153.86 | 897.70 | 81.91 | -339.56 | -594.70 | -84.42 | 16.03 | 0.95 | 16.45 | 0.43 | 0.42 | -1.23 | 2.07 | -812 | SE |
| [2] | 156 | 133 | 561.83 | 104.11 | 341.40 | 51.16 | 220.42 | -6.55 | 447.39 | 16.41 | 0.72 | 16.58 | 0.87 | 0.17 | -2.00 | 2.33 | 1,331 | NE |
| [3] | 132 | 528 | 574.44 | 115.72 | 698.21 | 64.04 | -123.77 | -384.62 | 137.09 | 15.61 | 0.75 | 16.54 | 0.44 | 0.93 | -0.74 | 2.61 | -133 | SE |
| [4] | 24 | 617 | 492.44 | 213.10 | 981.12 | 86.89 | -488.68 | -938.41 | -38.96 | 18.91 | 1.86 | 15.7 5 | 0.41 | -3.16 | -6.89 | 0.57 | 155 | SW |
| [5] | 3 | 198 |  |  |  |  |  |  |  |  |  |  |  |  |  |  |  |  |
| [6] | 153 | 947 | 572.20 | 105.46 | 888.54 | 61.07 | -316.34 | -553.41 | -79.28 | 16.18 | 0.70 | 16.33 | 0.33 | 0.15 | -1.34 | 1.65 | -2,053 | SE |
| [7] | 98 | 548 | 566.60 | 145.18 | 836.23 | 77.76 | -269.63 | -590.62 | 51.35 | 15.45 | 0.85 | 15.94 | 0.42 | 0.50 | -1.34 | 2.33 | -540 | SE |
| [8] | 58 | 597 | 553.76 | 141.63 | 863.91 | 79.20 | -310.15 | -627.02 | 6.73 | 17.28 | 1.20 | 16.25 | 0.42 | -1.03 | -3.51 | 1.46 | 302 | SW |
| [9] | 86 | 829 | 430.14 | 122.56 | 717.64 | 58.64 | -287.49 | -555.97 | -19.02 | 15.10 | 0.90 | 15.57 | 0.36 | 0.46 | -1.42 | 2.34 | -624 | SE |
| [10] | 70 | 316 | 723.61 | 172.69 | 1199.6 4 | 128.21 | -476.03 | -898.46 | -53.60 | 17.34 | 1.06 | 17.60 | 0.57 | 0.26 | -2.06 | 2.58 | -1,829 | SE |
| [11] | 126 | 973 | 378.29 | 79.39 | 836.35 | 63.35 | -458.06 | -655.55 | -260.58 | 15.57 | 0.80 | 16.23 | 0.34 | 0.66 | -1.03 | 2.36 | -692 | SE |
| [12] | 30 | 172 | 1274.81 | 399.18 | 935.78 | 201.86 | 339.04 | -558.74 | 1236.81 | 17.85 | 1.56 | 15.35 | 1.15 | -2.50 | -6.29 | 1.30 | -136  (dominated) | NW |

ICER: incremental cost-effectiveness ratio, CI: confidence interval, NE: north-east, NW: north-west, SE (under ICER quadrant): south-east, SE: standard error, SW: south-west

[1] Sensitivity analysis: Complete case analysis. Adjusted for age category, comorbidity, duration of illness and vaccination status.

[2] Sensitivity analysis: Concurrent randomisation analysis population. Adjusted for age category, comorbidity, duration of illness and vaccination status.

[3] Subgroup analysis: Aged 18-64 years. Adjusted for duration of illness and vaccination status.

[4] Subgroup analysis: Aged ≥65 years. Adjusted for comorbidity, duration of illness and vaccination status.

[5] Subgroup analysis: No comorbidity at baseline - numbers were too small for computation.

[6] Subgroup analysis: Had comorbidity at baseline. Adjusted for age category, duration of illness and vaccination status.

[7] Subgroup analysis: Duration of symptoms of <7 days before randomisation. Adjusted for age category, comorbidity and vaccination status.

[8] Subgroup analysis: Duration of symptoms of ≥7 days before randomisation. Adjusted for age category, comorbidity and vaccination status.

[9] Subgroup analysis: No symptoms rated as 'major' at baseline. Adjusted for age category, comorbidity, duration of illness and vaccination status.

[10] Subgroup analysis: At least one symptom rated as 'major' at baseline. Adjusted for age category, comorbidity, duration of illness and vaccination status.

[11] Subgroup analysis: Did not use inhaled corticosteroid steroid at randomisation/within 28 days follow-up. Adjusted for age category, comorbidity, duration of illness and vaccination status.

[12] Subgroup analysis: Used inhaled corticosteroid steroid at randomisation/within 28 days follow-up. Adjusted for age category, comorbidity, duration of illness and vaccination status.

## Table S32. Sensitivity and subgroup analyses of the cost-effectiveness analysis in terms of incremental cost per day saved until recovery: Usual care vs. Favipiravir

|  | **Number** | | **Cost** | | | | | | | **Time-to-recovery** | | | | | | | **ICER** | **ICER quadrant** |
| --- | --- | --- | --- | --- | --- | --- | --- | --- | --- | --- | --- | --- | --- | --- | --- | --- | --- | --- |
|  | **Favipiravir** | **Usual care** | **Favipiravir** | | **Usual care** | | **Mean difference** | **Lower 95%CI** | **Upper 95%CI** | **Favipiravir** | | **Usual care** | | **Mean difference** | **Lower 95%CI** | **Upper 95%CI** |  |  |
|  |  |  | **Mean** | **SE** | **Mean** | **SE** |  |  |  | **Mean** | **SE** | **Mean** | **SE** |  |  |  |  |  |
| [1] | 1829 | 2704 | 451.95 | 37.10 | 493.68 | 22.75 | -41.74 | -99.14 | 15.67 | 14.77 | 0.34 | 16.70 | 0.19 | 1.93 | 1.37 | 2.48 | -22 | SE |
| [2] | 1829 | 1668 | 452.76 | 18.62 | 385.06 | 16.99 | 67.71 | 18.22 | 117.20 | 15.02 | 0.21 | 16.80 | 0.22 | 1.77 | 1.18 | 2.36 | 38 | NE |
| [3] | 1524 | 2286 | 449.44 | 20.69 | 437.20 | 18.15 | 12.23 | -41.85 | 66.32 | 15.19 | 0.23 | 17.05 | 0.19 | 1.86 | 1.29 | 2.43 | 7 | NE |
| [4] | 305 | 970 | 469.38 | 46.46 | 787.05 | 58.52 | -317.67 | -463.79 | -171.55 | 13.83 | 0.50 | 15.73 | 0.32 | 1.90 | 0.75 | 3.04 | -168 | SE |
| [5] | 601 | 890 | 218.55 | 17.04 | 279.01 | 31.84 | -60.46 | -131.21 | 10.30 | 13.88 | 0.37 | 15.57 | 0.31 | 1.68 | 0.75 | 2.61 | -36 | SE |
| [6] | 1228 | 2366 | 567.39 | 25.68 | 640.14 | 27.19 | -72.75 | -145.88 | 0.38 | 15.45 | 0.25 | 17.08 | 0.20 | 1.63 | 1.01 | 2.25 | -45 | SE |
| [7] | 1347 | 2087 | 416.60 | 17.45 | 477.29 | 24.38 | -60.69 | -119.30 | -2.08 | 14.52 | 0.24 | 16.09 | 0.20 | 1.57 | 0.97 | 2.18 | -39 | SE |
| [8] | 482 | 1169 | 553.83 | 51.24 | 655.93 | 42.83 | -102.10 | -231.90 | 27.71 | 16.11 | 0.43 | 17.62 | 0.30 | 1.51 | 0.51 | 2.50 | -68 | SE |
| [9] | 1193 | 2234 | 398.26 | 22.95 | 490.37 | 24.61 | -92.12 | -158.21 | -26.03 | 13.81 | 0.25 | 15.87 | 0.20 | 2.06 | 1.44 | 2.67 | -45 | SE |
| [10] | 636 | 1022 | 555.01 | 32.64 | 653.03 | 44.31 | -98.02 | -205.51 | 9.47 | 17.05 | 0.35 | 18.45 | 0.30 | 1.40 | 0.53 | 2.27 | -70 | SE |
| [11] | 1477 | 2638 | 329.54 | 20.05 | 396.56 | 22.76 | -67.01 | -126.48 | -7.54 | 14.62 | 0.23 | 16.36 | 0.19 | 1.75 | 1.17 | 2.32 | -38 | SE |
| [12] | 352 | 618 | 967.45 | 36.34 | 1141.33 | 73.45 | -173.88 | -334.40 | -13.36 | 16.28 | 0.48 | 17.89 | 0.40 | 1.61 | 0.43 | 2.79 | -108 | SE |

ICER: incremental cost-effectiveness ratio, CI: confidence interval, NE: north-east, NW: north-west, SE (under ICER quadrant): south-east, SE: standard error, SW: south-west

[1] Sensitivity analysis: Complete case analysis. Adjusted for age category, comorbidity, duration of illness and vaccination status.

[2] Sensitivity analysis: Concurrent randomisation analysis population. Adjusted for age category, comorbidity, duration of illness and vaccination status.

[3] Subgroup analysis: Aged 18-64 years. Adjusted for comorbidity, duration of illness and vaccination status.

[4] Subgroup analysis: Aged ≥65 years. Adjusted for comorbidity, duration of illness and vaccination status.

[5] Subgroup analysis: No comorbidity at baseline. Adjusted for age category, duration of illness and vaccination status.

[6] Subgroup analysis: Had comorbidity at baseline. Adjusted for age category, duration of illness and vaccination status.

[7] Subgroup analysis: Duration of symptoms of <7 days before randomisation. Adjusted for age category, comorbidity and vaccination status.

[8] Subgroup analysis: Duration of symptoms of ≥7 days before randomisation. Adjusted for age category, comorbidity and vaccination status.

[9] Subgroup analysis: No symptoms rated as 'major' at baseline. Adjusted for age category, comorbidity, duration of illness and vaccination status.

[10] Subgroup analysis: At least one symptom rated as 'major' at baseline. Adjusted for age category, comorbidity, duration of illness and vaccination status.

[11] Subgroup analysis: Did not use inhaled corticosteroid steroid at randomisation/within 28 days follow-up. Adjusted for age category, comorbidity, duration of illness and vaccination status.

[12] Subgroup analysis: Used inhaled corticosteroid steroid at randomisation/within 28 days follow-up. Adjusted for age category, comorbidity, duration of illness and vaccination status.

## Table S33. Sensitivity and subgroup analyses of the cost-effectiveness analysis in terms of incremental cost per day saved until recovery: Usual care vs. Ivermectin

|  | **Number** | | **Cost** | | | | | | | **Time-to-recovery** | | | | | | | **ICER** | **ICER quadrant** |
| --- | --- | --- | --- | --- | --- | --- | --- | --- | --- | --- | --- | --- | --- | --- | --- | --- | --- | --- |
|  | **Ivermectin** | **Usual care** | **Ivermectin** | | **Usual care** | | **Mean difference** | **Lower 95%CI** | **Upper 95%CI** | **Ivermectin** | | **Usual care** | | **Mean difference** | **Lower 95%CI** | **Upper 95%CI** |  |  |
|  |  |  | **Mean** | **SE** | **Mean** | **SE** |  |  |  | **Mean** | **SE** | **Mean** | **SE** |  |  |  |  |  |
| [1] | 2157 | 2704 | 1002.83 | 35.36 | 490.41 | 22.69 | 512.42 | 459.29 | 565.55 | 15.59 | 0.33 | 16.66 | 0.19 | 1.07 | 0.54 | 1.60 | 480 | NE |
| [2] | 2157 | 1806 | 1003.58 | 26.43 | 387.25 | 15.72 | 616.34 | 574.70 | 657.97 | 15.66 | 0.36 | 16.78 | 0.22 | 1.11 | 0.54 | 1.68 | 553 | NE |
| [3] | 1811 | 2286 | 1001.02 | 15.32 | 437.20 | 17.97 | 563.81 | 517.67 | 609.95 | 15.86 | 0.21 | 17.05 | 0.20 | 1.19 | 0.64 | 1.75 | 473 | NE |
| [4] | 346 | 970 | 1013.76 | 46.54 | 787.05 | 59.84 | 226.71 | 77.41 | 376.01 | 15.32 | 0.51 | 15.66 | 0.32 | 0.34 | -0.82 | 1.49 | 672 | NE |
| [5] | 697 | 890 | 761.02 | 18.79 | 279.01 | 31.77 | 482.01 | 409.91 | 554.12 | 14.27 | 0.34 | 15.61 | 0.31 | 1.34 | 0.46 | 2.22 | 360 | NE |
| [6] | 1460 | 2366 | 1118.61 | 19.26 | 640.14 | 27.33 | 478.47 | 412.60 | 544.34 | 16.45 | 0.24 | 17.02 | 0.20 | 0.57 | -0.03 | 1.17 | 838 | NE |
| [7] | 1556 | 2087 | 964.61 | 15.52 | 477.29 | 24.12 | 487.32 | 430.59 | 544.05 | 15.32 | 0.23 | 16.08 | 0.20 | 0.76 | 0.17 | 1.34 | 642 | NE |
| [8] | 601 | 1169 | 1102.61 | 35.35 | 655.93 | 43.28 | 446.68 | 336.17 | 557.18 | 16.82 | 0.39 | 17.60 | 0.29 | 0.78 | -0.17 | 1.73 | 574 | NE |
| [9] | 1418 | 2234 | 981.68 | 17.31 | 490.37 | 24.28 | 491.31 | 432.92 | 549.70 | 14.75 | 0.25 | 15.82 | 0.20 | 1.07 | 0.46 | 1.68 | 457 | NE |
| [10] | 739 | 1022 | 1044.08 | 27.59 | 653.03 | 43.88 | 391.06 | 289.9 7 | 492.15 | 17.66 | 0.33 | 18.49 | 0.30 | 0.83 | -0.01 | 1.68 | 469 | NE |
| [11] | 1737 | 2621 | 869.71 | 13.90 | 379.47 | 22.70 | 490.24 | 438.11 | 542.38 | 15.50 | 0.22 | 16.34 | 0.19 | 0.84 | 0.28 | 1.40 | 586 | NE |
| [12] | 420 | 635 | 1554.55 | 39.82 | 1195.09 | 80.05 | 359.47 | 183.57 | 535.36 | 16.71 | 0.46 | 17.87 | 0.40 | 1.17 | 0.01 | 2.32 | 309 | NE |

ICER: incremental cost-effectiveness ratio, CI: confidence interval, NE: north-east, NW: north-west, SE (under ICER quadrant): south-east, SE: standard error, SW: south-west

[1] Sensitivity analysis: Complete case analysis. Adjusted for age category, comorbidity, duration of illness and vaccination status.

[2] Sensitivity analysis: Concurrent randomisation analysis population. Adjusted for age category, comorbidity, duration of illness and vaccination status.

[3] Subgroup analysis: Aged 18-64 years. Adjusted for comorbidity, duration of illness and vaccination status.

[4] Subgroup analysis: Aged ≥65 years. Adjusted for comorbidity, duration of illness and vaccination status.

[5] Subgroup analysis: No comorbidity at baseline. Adjusted for age category, duration of illness and vaccination status.

[6] Subgroup analysis: Had comorbidity at baseline. Adjusted for age category, duration of illness and vaccination status.

[7] Subgroup analysis: Duration of symptoms of <7 days before randomisation. Adjusted for age category, comorbidity and vaccination status.

[8] Subgroup analysis: Duration of symptoms of ≥7 days before randomisation. Adjusted for age category, comorbidity and vaccination status.

[9] Subgroup analysis: No symptoms rated as 'major' at baseline. Adjusted for age category, comorbidity, duration of illness and vaccination status.

[10] Subgroup analysis: At least one symptom rated as 'major' at baseline. Adjusted for age category, comorbidity, duration of illness and vaccination status.

[11] Subgroup analysis: Did not use inhaled corticosteroid steroid at randomisation/within 28 days follow-up. Adjusted for age category, comorbidity, duration of illness and vaccination status.

[12] Subgroup analysis: Used inhaled corticosteroid steroid at randomisation/within 28 days follow-up. Adjusted for age category, comorbidity, duration of illness and vaccination status.

**Trial Title: P**latform **R**andomised tr**I**al of treatme**N**ts in the **C**ommunity for ep**I**demic and

**P**andemic i**L**ln**E**sses

**Internal Reference Number / Short title:** PRINCIPLE

**Ethics Ref:** 20/SC/0158

**IRAS Project ID:** 281958

**EudraCT Number**: 2020-001209-22

**Date and Version No**: 03 Jan 2025 Version 1.0

# HEALTH ECONOMIC ANALYSIS PLAN

| Author  (Trial Health Economist) | May Ee Png  Email: may.png@phc.ox.ac.uk |
| --- | --- |
| Reviewer  (Lead Health Economist) | Stavros Petrou  Email: stavros.petrou@phc.ox.ac.uk |
| Reviewer  (Lead Trial Statistician) | Ly-Mee Yu  Email: ly-mee.yu@phc.ox.ac.uk |
| Approver  (Chief Investigator) | Christopher Butler  Nuffield Department of Primary Care Health Sciences  Radcliffe Observatory Quarter  Woodstock Rd  Oxford  OX2 6GG  Email: christopher.butler@phc.ox.ac.uk |

**TABLE OF CONTENTS**

[1.	Background and rational	3](#_Toc177404707)

[1.1 Purpose of the health economics analysis plan	3](#_Toc177404708)

[1.2 Background	3](#_Toc177404709)

[1.3 Aims and Objectives	3](#_Toc177404710)

[2.	Trial design	3](#_Toc177404711)

[2.1 Inclusion criteria	4](#_Toc177404712)

[2.2 Exclusion criteria	5](#_Toc177404713)

[3.	Economic approach	7](#_Toc177404714)

[3.1 Aims and objectives	7](#_Toc177404715)

[3.2 Overview of economic analysis	7](#_Toc177404716)

[4.	Data collection and management	7](#_Toc177404717)

[4.1 Measurement of resource use data	7](#_Toc177404718)

[4.2 Valuation of resource use data	8](#_Toc177404719)

[4.3 Measurement of outcome	8](#_Toc177404720)

[4.4 Data cleaning	8](#_Toc177404721)

[4.5 Missing data	8](#_Toc177404722)

[5.	Data analysis	8](#_Toc177404723)

[5.1 Uncertainty analysis	9](#_Toc177404724)

[5.2 Sensitivity analysis	9](#_Toc177404725)

[5.3 Subgroup analysis	9](#_Toc177404726)

[6.	Reporting of results	10](#_Toc177404727)

[7.	References	10](#_Toc177404728)

[8.	Appendix A: Amendment History	11](#_Toc177404729)

[9.	Appendix B: Abbreviations	12](#_Toc177404730)

1. **Background and rational**

**1.1 Purpose of the health economics analysis plan**

The purpose of this health economic analysis plan (HEAP) is to describe the analysis and reporting procedure intended for the economic analyses to be undertaken. The analysis plan is designed to ensure that there is no conflict with the protocol and associated statistical analysis plan (SAP) and it should be read in conjunction with them. This HEAP largely follows the guidance as suggested by Thorn et al.^1^. This document has been written based on information contained in the trial protocol version 14.0, dated 8 August 2022 and SAP version 5.0, dated 14 December 2021.

**1.2 Background**

SARS-CoV-2, which causes COVID-19, has infected more than 676 million people and claimed 14.9 million lives between 2020 and 2021.^2,3^ In the UK, persisting symptoms that arise from COVID-19 and last at least four weeks after acute infection have adversely affected the day-to-day activities of 1.5 million people, with 20% being "limited a lot” in their day-to-day activities.^4^ There is an urgent need to identify effective intervention(s) among the potential COVID-19 treatments that are available for rapid pragmatic evaluation and for use in the community, particularly among those who are at higher risk of complications, such as those aged 50 years and over with comorbidity and those aged 65 years and over.

**1.3 Aims and Objectives**

**Main Trial:** The primary aim is to assess the effectiveness of treatments in reducing time to feeling recovered and the need for hospital admission (or mortality) among participants with possible COVID-19 in the community and who are at higher risk of a complicated illness course within 28-day of randomisation.

2. **Trial design**

PRINCIPLE is an open-label, multi-arm, prospective, platform, adaptive, randomised controlled clinical trial in the community care. Participants were randomised to one study arm using equal allocation ratios corresponding to the number of eligible arms in the trial. Trial arms included:

| Intervention arms | Date first participant randomised | Date last participant randomised to be included in the analysis |
| --- | --- | --- |
| Usual care plus hydroxychloroquine, 200mg twice daily for 3 days | 2 Apr 2020 | 30 Nov 2020 |
| Usual care plus azithromycin, 500mg once daily for 3 days | 23 May 2020 | 30 Nov 2020 |
| Usual care plus doxycycline, 200mg on day 1 followed by 100mg daily for 6 days | 24 Jul 2020 | 14 Dec 2020 |
| Usual care plus the inhaled corticosteroid, budesonide, 400mcg daily (as two puffs bd) for 14 days | 1 Dec 2020 | 31 Mar 2021 |
| Usual care plus colchicine, 500 micrograms (μg) once daily for 14 days | 4 Mar 2021 | 26 May 2021 |
| Usual care plus favipiravir 1800mg twice a day on day one, and then 800mg twice a day for four days | 8 Apr 2021 | 1 July 2022 |
| Usual care plus ivermectin, one dose each day for 3 days at 300μg/kg body weight | 23 Jun 2021 | 1 July 2022 |

**Comparator arm**: Usual Care, defined as the currently recommended treatment delivered by responsible clinicians, follows current National Health Service (NHS) care provision.

**2.1 Inclusion criteria**

The inclusion criteria relevant to all intervention arms include:

- Not hospitalised.
- Participant or their legal representative, is willing and able to give informed consent for participation in the study and is willing to comply with all trial procedures.

The inclusion criteria specific to the intervention arm are listed in the table below.

| Intervention arms | Inclusion criteria |
| --- | --- |
| Usual care plus azithromycin | - Aged ≥65 years, or 50–64 years with comorbidities*. - Had ongoing symptoms from PCR-confirmed COVID-19 or suspected COVID-19 (in accordance with the UK NHS definition of high temperature and/or new, continuous cough and/or change in sense of smell/ taste) that had started within the previous 14 days. |
| Usual care plus doxycycline |  |
| Usual care plus budesonide |  |
| Usual care plus colchicine | - Aged ≥65 years, or 18–64 years with comorbidities* or shortness of breath. - Had ongoing symptoms from PCR-confirmed COVID-19 or suspected COVID-19 (in accordance with the UK NHS definition of high temperature and/or new, continuous cough and/or change in sense of smell/ taste) that had started within the previous 14 days. |
| Usual care plus favipiravir | - Aged ≥65 years, or 18–64 years with comorbidities* or shortness of breath. - Had ongoing symptoms from PCR-confirmed COVID-19 or suspected COVID-19 (in accordance with the UK NHS definition of high temperature and/or new, continuous cough and/or change in sense of smell/ taste) that had started within the previous 14 days. - From 29th July 2021, all patients aged 18 years or over with a positive SARS-CoV-2 test (lateral flow or PCR) and who were within 14 days of the onset of ongoing symptoms consistent with COVID-19 were eligible for inclusion. Requirement for comorbidity and shortness of breath was removed. - Women of childbearing potential (premenopausal female that is anatomically and physiologically capable of becoming pregnant**), willing to take a highly sensitive pregnancy test and reports a negative test result**. |
| Usual care plus ivermectin | - Aged ≥65 years, or 18–64 years with comorbidities* or shortness of breath. - Had ongoing symptoms from PCR-confirmed COVID-19 or suspected COVID-19 (in accordance with the UK NHS definition of high temperature and/or new, continuous cough and/or change in sense of smell/ taste) that had started within the previous 14 days. - From 29th July 2021, all patients aged 18 years or over with a positive SARS-CoV-2 test (lateral flow or PCR) and who were within 14 days of the onset of ongoing symptoms consistent with COVID-19 were eligible for inclusion. Requirement for comorbidity and shortness of breath was removed. |

*Comorbidities required for eligibility were known weakened immune system due to a serious illness or medication (e.g. chemotherapy); known heart disease or a diagnosis of high blood pressure; known asthma or lung disease; known diabetes; known mild hepatic impairment; or known stroke or neurological problems; self-report obesity or body mass index ≥35 kg/m^2^.

** As recorded by the participant on the screening form and confirmed on Day 1 by a call between clinician and participant.

**2.2 Exclusion criteria**

The exclusion criteria relevant to all intervention arms consist of:

- Participant currently admitted in hospital.
- Previous randomisation to an arm in the PRINCIPLE trial.
- Previous randomisation to another CTIMP for COVID-19 therapeutics, e.g. PANORAMIC, during the patient’s current, qualifying COVID-19 diagnosis.
- Almost recovered (general condition much improved and COVID-19 symptoms now mild or almost absent).
- In the judgement of the recruiting clinician was deemed ineligible.
- Already receiving antibiotics for an acute condition.
- Currently taking the intervention.
- Previous adverse reaction to intervention.
- Had a contraindication to the intervention as identified in the summary of product characteristics and British National Formulary.
- Known or suspected pregnancy.
- Breastfeeding.

The exclusion criteria specific to the intervention arm are listed in the table below.

| Intervention arms | Exclusion criteria |
| --- | --- |
| Usual care plus azithromycin | - Previous reaction to other macrolides or ketolides. - Known severe hepatic impairment. - Known severe renal impairment. - Known myasthenia gravis. - Known congenital or documented QT prolongation. - Known allergy to soya or peanut due to the risk of hypersensitivity reactions. - Taking hydroxychloroquine or chloroquine, sotalol, amiodarone, ciclosporin, digoxin, bromocriptine, cabergoline, ergotamine, ergometrine, methysergide or any ergot derivatives. |
| Usual care plus doxycycline | - Previous reaction to other tetracyclines. - Known myasthenia gravis. - Systemic lupus erythematosus. - Sucrose intolerance (i.e. rare hereditary problems of fructose intolerance, glucose galactose malabsorption or sucrose-isomaltase insufficiency). - Taking ciclosporin, retinoids (acitretin, alitretinoin, isotretinoin, tretinoin), methotrexate, ergotamine, methoxyflurane, lithium. |
| Usual care plus budesonide | - Already taking inhaled or systemic corticosteroids. - Unable to use an inhaler. |
| Usual care plus colchicine | - Hypersensitivity to the active substance or to any of the excipients (Lactose, Pregelatinised Maize Starch, Stearic Acid, Purified Talc, Purified Water, Ethanol 96%). - Inflammatory bowel disease or chronic diarrhoea. - Known blood dyscrasias. - Known severe renal impairment or requiring dialysis. - Known severe hepatic impairment. - Taking colchicine, clarithromycin, erythromycin, ketoconazole, itraconazole, voriconazole, HIV protease inhibitors (e.g. ritonavir, atazanavir), cobicistat, verapamil, diltiazem, cyclosporin, quinidine, disulfiram. - Consumption of grapefruit. - Women of childbearing potential (premenopausal female that is anatomically and physiologically capable of becoming pregnant*) and not prepared to use highly effective contraception** for the 28-day duration of follow up in the study. |
| Usual care plus favipiravir | - Gout. - Severe liver disease. - Women of childbearing potential (premenopausal female that is anatomically and physiologically capable of becoming pregnant*) or men with a partner of childbearing potential not willing to use highly effective contraception** for the 28-day duration of the trial. |
| Usual care plus ivermectin | - Allergy to any of its excipients. - Ever having travelled to countries endemic for Loa loa. - Bleeding diatheses. - Severe liver disease. - Grapefruit consumption. - Taking quinidine, amiodarone, diltiazem, spironolactone, verapamil, clarithromycin, erythromycin, itraconazole, ketoconazole, cyclosporine, tacrolimus, sirolimus, indinavir, ritonavir, cobicistat, warfarin. - Women of childbearing potential (premenopausal female that is anatomically and physiologically capable of becoming pregnant*) and not prepared to use highly effective contraception** for the 28-day duration of follow up in the study. |

* As recorded by the participant on the screening form and confirmed on Day 3 telephone call for colchicine and ivermectin arms, and Day 1 telephone call for favipiravir arm.

**Highly effective methods have typical-use failure rates of less than 1% and include male or female sterilisation and long-acting reversible contraceptive (LARC) methods (intrauterine devices and implants). A couple using another method of contraception, such as a combined hormonal method, progestogen only pill or injection, are eligible if they are willing to use an additional barrier method (e.g. male condom) for the 28-day duration of follow-up in the trial. Note: a barrier method on its own is not sufficient.

3. **Economic approach**

**3.1 Aims and objectives**

The primary objective of the health economic evaluation is to estimate the cost-effectiveness of all the trial interventions within the PRINCIPLE trial except for hydroxychloroquine over a 12-month time horizon. Hydroxychloroquine is excluded from the cost-effectiveness analysis because the participants were not vaccinated and we do not know what proportion had COVID-19 as we were not able to test people at that time for COVID-19.

**3.2 Overview of economic analysis**

Within-trial cost-effectiveness analyses of all trial interventions within the PRINCIPLE trial, except hydroxychloroquine, will be performed using individual participant-level data from PRINCIPLE after the clinical trial analysis has concluded. Based on trial evidence, incremental cost-effectiveness ratios will be calculated by taking a ratio of the difference in the mean costs and the co-primary outcomes, i.e. mean hospitalisation or death avoided and mean day saved until recovery. This trial is conducted in the UK, which has an NHS, providing publicly funded healthcare, primarily free of charge at the point of use. Participants were recruited through general practice, health service helplines, COVID-19 ‘hot hubs’ and directly through the trial website. The primary economic analysis will be from the NHS and personal social services (PSS) perspective and will compare the costs and consequences of each arm over 28 days after randomisation for hospitalisation or death avoided and time to recovery. Sensitivity and subgroup analyses will also be performed.

4. **Data collection and management**

**4.1 Measurement of resource use data**

Resource use data were collected using a daily diary completed over the first 28 days post-randomisation and trial questionnaires given to participants at three-, six-months and 12-months post randomisation for self-completion. Contacts with health services were complemented with general practitioner (GP) notes review where available through Oxford Royal College of General Practitioners Research Surveillance Centre (RCGP RSC) or Admitted Patient Care and Critical Care data from the NHS Digital. The data sources were considered in the following hierarchy: (1) Participant reported (daily diaries, calls, hospitalisation case report form), (2) Notes review, (3) data from NHS digital, as per the data hierarchy stated in the SAP.

The frequency of use of primary care (i.e. GP) and secondary care services (i.e. hospital admission, emergency care, and hospital outpatient clinic) due to symptoms associated with COVID-19 were recorded in the daily diaries and trial participant completed questionnaires. Time off work due to COVID-19 symptoms was also captured in the trial participant completed questionnaires.

**4.2 Valuation of resource use data**

All resource use will be valued in monetary terms using the latest and most appropriate UK unit costs or participant valuations estimated at the time of analysis. Adjustments will be made for inflation using the Personal Social Services Research Unit (PSSRU) hospital & community health services index^5^ where applicable. Unit cost of trial treatments and other medications will be obtained from the Prescription Cost Analysis^6^ and literature if it is not available in the Prescription Cost Analysis. The cost of dispensing the medications will not be included because it is considered to be approximately equal between the comparator and interventions arms. NHS reference costs^7^ will be employed to value hospital resource use (e.g. inpatient visits that include day cases and longer stays [i.e. elective and non-elective admissions], emergency department visits and outpatient attendances) while the PSSRU Unit Costs of Health and Social Care^5^ will be used to value the primary care services. The daily gender-specific median wage, obtained from the Office for National Statistics, will be used in the computation of the economic value of time taken off work by the participants due to COVID-19^8^.

**4.3 Measurement of outcome**

The trial has two co-primary clinical outcomes that we will include in the cost-effectiveness analysis: time taken to self-reported recovery and the number of hospitalisations or deaths avoided at 28 days post-randomisation. An additional hospitalisation or deaths avoided at 12 months post-randomisation will be included in the sensitivity analysis. Measurements were recorded using the daily diaries over the first 28 days post randomisation and trial questionnaires at baseline. Additionally, trial questionnaires measured hospitalisation or deaths avoided at three-months, six-months and 12-months post randomisation. Relevant measurements from medical notes review, RSC data and NHS Digital data were also collected. Time to recovery is defined as the first instance that a participant reports feeling recovered. Hospitalisation is defined as an overnight non-elective stay in hospital while death refer to all-cause mortality.

**4.4 Data cleaning**

Face validity tests will be conducted on data (e.g. to identify misspelt text) and checked against the source documents. Records of resource use across different time-points will also be cross-checked to ensure that there is no duplication. Corrections made will be documented in the statistical code.

**4.5 Missing data**

Before carrying out the within trial analysis, the trial data will be checked for any missing data. Where possible, the reasons for missing data will be ascertained, using the methodology described by Faria et al^9^, and reported. The nature and pattern of ‘missingness’ will be carefully considered; in particular, whether data can be treated as missing at random (MAR) and multiple imputation methods may be used if the data is MAR. Costs will be imputed at the total cost level per time-point and outcomes will be imputed at each time-point if multiple imputation was to be applied.

5. **Data analysis**

The population in the base case analysis of each intervention (known as ‘primary analysis population’) will be all eligible SARS-CoV-2-positive participants randomly assigned to the studied intervention, usual care, and other interventions, from the start of the platform trial until the studied intervention group was closed.

The data analysis will include within-trial analyses using imputed costs and outcomes data that will include all randomised participants in the intervention arm versus usual care treatment arm, in accordance to the “intention to treat” (ITT) principle, taking a 28-days’ time horizon for the base case analysis and 12-months’ time horizon for the sensitivity analysis.

Utilisation of resource use items will be summarised by trial allocation group and follow-up period and differences between groups will be analysed using t-tests for continuous variables and Pearson chi-squared (χ^2^) test for categorical variables. Means and standard errors for values of each cost category will be estimated by treatment allocation and follow-up period. Mean differences in costs and outcomes between the treatment arms will be estimated using t-tests and the bootstrap 95% confidence interval that will be computed based on 10,000 replications. This bootstrap will use Monte Carlo simulations to resample many datasets based on the original data. Costs and benefits will not be discounted since the follow-up is only 12 months.

Cost and time to recovery will be combined to calculate incremental cost-effectiveness ratios (ICERs) expressed in terms of incremental cost per day saved until recovery. Likewise, cost and hospitalisation or death avoided will be combined to calculate ICERs expressed in terms of incremental cost per hospitalisation or death avoided. All analyses will be carried out using appropriate analytical software such as STATA, R or Microsoft Excel. The relevant package and version number will be recorded in the health economics report.

**5.1 Uncertainty analysis**

A nonparametric bootstrapping approach will be used to determine the level of sampling uncertainty surrounding the mean ICER by generating 10,000 estimates of incremental costs and benefits. We will review the literature for any revealed preference and stated preference on the economic value placed on the primary outcomes (i.e. avoiding COVID-19 hospitalisations or deaths and time to recovery from COVID-19) and use it to inform selection of cost-effectiveness thresholds that examine decision uncertainty.

**5.2 Sensitivity analysis**

Four sensitivity analyses will be undertaken to explore uncertainties surrounding key parameters in the economic evaluation as described in the following. First, the study perspective will be extended to a societal perspective which includes indirect costs (e.g. valuation of time off work and lost productivity). Second, complete case analysis will be used to assess the impact of missing data on the ICERs. Third, ICER expressed in terms of incremental cost per hospitalisation or death avoided over the 12 months will be computed. Last, a concurrent randomisation analysis consisting of all eligible SARS-CoV-2-positive participants will be conducted based on participants who were randomised to the usual care arm during the same time frame when the intervention was actively randomised and who were eligible for randomisation to the intervention.

**5.3 Subgroup analysis**

Subgroup analysis will be done as per the subgroup analysis planned in the SAP. It will be dependent on number of participants per category and if necessary, categories may be combined to avoid small cell sizes.

- Age group (<65/≥65)
- Presence of comorbidity at baseline (yes/no)
- Duration of symptoms prior to randomisation (<7 days vs. ≥7 days)
- Severity of symptoms at baseline (at least one symptom rated major vs no symptoms rated major)
- Using an inhaled corticosteroid steroid (ICS) at randomisation or during 28 days of follow-up (yes/no). Participant report will be taken as the primary source and only if missing will prescription of ICS in the two months prior to randomisation or during follow up from notes review or NHS Digital be considered.

6. **Reporting of results**

CHEERS II guidelines^10^ will be followed when reporting the health economic evaluation, in a format appropriate to stakeholders and policy makers. Any deviation from HEAP will be described and justified in the final published report.

7. **References**

1	Thorn J, Davies C, Brookes S, Noble S, Dritsaki M, Gray E, *et al.* Content of Health Economics Analysis Plans (HEAPs) for trial-based economic evaluations: expert Delphi consensus survey. *Value in Health* 2021;**24**:539–47.

2	Center for Systems Science and Engineering (CSSE) at Johns Hopkins University (JHU). *COVID-19 dashboard*. 2023. URL: https://www.arcgis.com/apps/dashboards/bda7594740fd40299423467b48e9ecf6 (Accessed 20 April 2023).

3	World Health Organization. *14.9 million excess deaths associated with the COVID-19 pandemic in 2020 and 2021*. 2022. URL: https://www.who.int/news/item/05-05-2022-14.9-million-excess-deaths-were-associated-with-the-covid-19-pandemic-in-2020-and-2021 (Accessed 19 June 2023).

4	Office for National Statistics. *Prevalence of ongoing symptoms following coronavirus (COVID-19) infection in the UK: 30 March 2023*. 2023. URL: https://www.ons.gov.uk/peoplepopulationandcommunity/healthandsocialcare/conditionsanddiseases/bulletins/prevalenceofongoingsymptomsfollowingcoronaviruscovid19infectionintheuk/30march2023 (Accessed 30 October 2023).

5	Curtis L, Beecham J, Burns A. *Unit Costs of Health and Social Care*. 2021. URL: https://www.pssru.ac.uk/research/354/ (Accessed 23 April 2021).

6	Business Services Authority. *Prescription Cost Analysis (PCA) data*. 2021. URL: https://www.nhsbsa.nhs.uk/prescription-data/dispensing-data/prescription-cost-analysis-pca-data (Accessed 23 April 2021).

7	National Health Service. *National Cost Collection for the NHS*. 2022. URL: https://www.england.nhs.uk/national-cost-collection (Accessed 12 February 2022).

8	Office for National Statistics. *Earnings and Working Hours*. 2022. URL: https://www.ons.gov.uk/employmentandlabourmarket/peopleinwork/earningsandworkinghours (Accessed 12 February 2022).

9	Faria R, Gomes M, Epstein D, White IR. A Guide to Handling Missing Data in Cost-Effectiveness Analysis Conducted Within Randomised Controlled Trials. *Pharmacoeconomics* 2014;**32**:1157–70. https://doi.org/10.1007/s40273-014-0193-3.

10	Husereau D, Drummond M, Augustovski F, de Bekker-Grob E, Briggs A, Carswell C, *et al.* Consolidated health economic evaluation reporting standards (CHEERS) 2022 explanation and elaboration: a report of the ISPOR CHEERS II Good Practices Task Force. *Value in Health* 2022;**25**:10–31.

8. **Appendix A: Amendment History**

Lists details of all HEAP amendments whenever a new version of the HEAP is produced.

| HEAP version no., Issue date | Protocol version no., Issue date | Name of person who made the change(s) | Date changed | Significant change(s) from previous version together with reasons |
| --- | --- | --- | --- | --- |
|  |  |  |  |  |
|  |  |  |  |  |
|  |  |  |  |  |
|  |  |  |  |  |
|  |  |  |  |  |
|  |  |  |  |  |

9. **Appendix B: Abbreviations**

| CHEERS | Consolidated Health Economic Evaluation Reporting Standards |
| --- | --- |
| GP | General Practitioner |
| HEAP | Health Economic Analysis Plan |
| ICER | Incremental Cost-Effectiveness Ratio |
| ICS | Inhaled Corticosteroid Steroid |
| ITT | Intention to Treat |
| LARC | Long-Acting Reversible Contraceptive |
| MAR | Missing at Random |
| NHS | National Health Service |
| PCR | Polymerase Chain Reaction |
| PSS | Personal Social Service |
| PSSRU | Personal Social Services Research Unit |
| RCGP RSC | Royal College of General Practitioners Research Surveillance Centre |
| SAP | Statistical Analysis Plan |
